# Plasma and cerebrospinal fluid proteomic signatures of acutely sleep-deprived humans: an exploratory study

**DOI:** 10.1093/sleepadvances/zpad047

**Published:** 2023-11-14

**Authors:** Ana Vaquer-Alicea, Jinsheng Yu, Haiyan Liu, Brendan P Lucey

**Affiliations:** Department of Neurology, Washington University School of Medicine, St Louis, MO, USA; Department of Genetics, Washington University School of Medicine, St Louis, MO, USA; Department of Neurology, Washington University School of Medicine, St Louis, MO, USA; Department of Neurology, Washington University School of Medicine, St Louis, MO, USA

**Keywords:** sleep deprivation, mRNA and protein expression, sleep and the brain

## Abstract

**Study Objectives:**

Acute sleep deprivation affects both central and peripheral biological processes. Prior research has mainly focused on specific proteins or biological pathways that are dysregulated in the setting of sustained wakefulness. This exploratory study aimed to provide a comprehensive view of the biological processes and proteins impacted by acute sleep deprivation in both plasma and cerebrospinal fluid (CSF).

**Methods:**

We collected plasma and CSF from human participants during one night of sleep deprivation and controlled normal sleep conditions. One thousand and three hundred proteins were measured at hour 0 and hour 24 using a high-scale aptamer-based proteomics platform (SOMAscan) and a systematic biological database tool (Metascape) was used to reveal altered biological pathways.

**Results:**

Acute sleep deprivation decreased the number of upregulated and downregulated biological pathways and proteins in plasma but increased upregulated and downregulated biological pathways and proteins in CSF. Predominantly affected proteins and pathways were associated with immune response, inflammation, phosphorylation, membrane signaling, cell-cell adhesion, and extracellular matrix organization.

**Conclusions:**

The identified modifications across biofluids add to evidence that acute sleep deprivation has important impacts on biological pathways and proteins that can negatively affect human health. As a hypothesis-driving study, these findings may help with the exploration of novel mechanisms that mediate sleep loss and associated conditions, drive the discovery of new sleep loss biomarkers, and ultimately aid in the identification of new targets for intervention to human diseases.

## Introduction

Sleep is a critical and complex process that plays a vital role in maintaining homeostasis, regulating metabolism, supporting immunity, and promoting development. It is driven by a homeostatic process that controls the need for sleep and is synchronized to circadian rhythms that respond to light and dark cues in the environment [1–6]. Unfortunately, approximately one-third of adults experience short or insufficient sleep, defined as less than 7 hours of sleep in a 24-hour period. Lifestyle and external factors, such as excessive media consumption and shift work, also contribute to this growing problem. Insufficient sleep, even for just a few days, has significant adverse effects on health and cognition. These effects can include obesity [7], inflammation [7–9], type 2 diabetes [10], muscle mass loss [11], and autoimmunity [12]. Sleep deprivation is also associated with significant negative economic, social, and academic outcomes [13]. As a result, insufficient sleep is now recognized as a public health issue and understanding how sleep deprivation mediates disease is increasingly recognized as a modifiable disease risk factor.

Previous studies in humans and other animal models have shown that acute sleep deprivation is associated with detrimental central and systemic effects. For instance, acute sleep deprivation can have behavioral effects such as impaired attention and working memory [14, 15], increased anxiety levels [16], and increased desire for high-caloric foods [17]. Acute sleep deprivation increases hippocampal protein expression [18], phosphorylation of the brain proteome [19], production/release of proteins (amyloid-beta, tau) associated with Alzheimer’s disease (AD) [20, 21], and microglial activity and amyloid deposition [22] while also decreasing cerebrospinal fluid (CSF)-to-blood clearance of amyloid-beta and tau [23]. It has also been associated with systemic effects such as increased inflammatory markers [16], vascular dysfunction [24], cortisol levels [25], altered epigenetic and transcriptional profile of core circadian clock genes [26], reduced muscle protein synthesis and anabolic hormone environment [27]. These studies have focused on particular proteins and biological processes, and very few use paired tissue and/or biofluid samples. Additionally, the availability of new high-throughput technologies over time has allowed increasing levels of characterization of tissue proteomics that have yet to be used in the context of sleep deprivation.

To address the need for a more comprehensive examination of protein expression and biological processes affected by acute sleep deprivation in humans, we employed a within-participant designed study to characterize the proteomic landscape in previously collected paired samples of plasma and CSF from human participants at baseline and then after either 24 hours of sleep deprivation or control normal sleep. Samples were processed through SOMAscan, a high-scale aptamer proteomic platform that measures 1300 proteins in total, and Metascape, a systematic biological database tool for pathway and process enrichment analysis. The aim of this study was to characterize the effects of sleep deprivation on expression of assayed proteins and biological pathways in the plasma and CSF of human participants to provide insights into potential mechanisms by which sleep disturbances can lead to adverse health outcomes.

## Methods

### Participant characteristics

Participant characteristics and study protocol are shown in Table 1. Plasma and CSF samples were collected as part of studies investigating the effect of sleep on AD biomarkers [20, 21, 23, 28]. We analyzed plasma and CSF samples from five cognitively unimpaired participants (two males and three females) who were recruited from either longitudinal studies at the Knight Alzheimer’s Disease Research Center or a research volunteer registry (Volunteer for Health) at Washington University in St. Louis. Participants were 30–60 years old and underwent normal sleep control and sleep deprivation conditions (to control for inter-individual differences) approximately 4–6 months apart to allow for adequate healing post lumbar catheter placement. As this was a within-participant study, there were no group differences for sex, race, and BMI. Differences in age between groups are due to the period between interventions. Participants were cognitively unimpaired (mini-mental state examination ≥27 [29] or clinical dementia rating 0 [30]), and with no clinical sleep disorders or neurological issues. The study protocol was approved by the Washington University institutional review board in the General Clinical Research Center advisory committee. All participants consented to the study and were compensated for their participation.

**Table 1.** Participant Characteristics

	Control (*N* = 5)	Sleep-deprived (*N* = 5)
Age, years Mean (SD)	48.12 (10.18)	48.02 (10.3)
Sex: M/F	2/3	2/3
Race: C/AA	2/3	2/3
MMSE Mean (SD)	28.8 (0.84)	28.8 (0.17)
Overnight TST (min) Mean (SD)	410.2 (52.7)	27.9 (27.1)
Overnight SE (%)	73.6 (9.8)	5.1 (4.9)

TST: total sleep time; SE: sleep efficiency; MMSE: mini-mental state examination; M: male; F: female; C: Caucasian; AA: African-American; SD: standard deviation.

### Biofluid collection and processing

Participants were admitted for an acclimation night at the Clinical Translational Research Unit and a lumbar catheter was placed the next day at approximately 07:00 for longitudinal CSF sampling. Intravenous lines were simultaneously placed for longitudinal collection of blood. Sleep–wake activity was monitored through polysomnography for the duration of the experiment. Starting at 0700, participants had 6 mL of CSF and 6 mL of blood drawn every 2 hours for 36 hours. However, for the purposes of our study, we only used samples from hours 0 and hour 24 to control for time-of-day effects.

All participants were kept awake until 2100 after which time they were allowed to sleep if undergoing the normal sleep control condition or remained awake if undergoing the sleep deprivation condition. During the sleep-deprived condition, participants were kept awake by nursing staff and did not receive stimulants. Participants were monitored with polysomnography and video to ensure they were awake. Participants were awake at 0700 on day 2. Lumbar catheter was removed on day 2 at 1900, after which participants laid flat for 12 hours. Overall, they received meals at 0900, 1300, and 1800 with snacks at 1100, 1500, and 2000. Blood samples were centrifuged to extract plasma. CSF and plasma samples were made into 0.5 mL aliquots and stored at−80^o^C. These samples were thawed and re-frozen twice before our use.

### Proteomic profiling and data processing and validation

Proteins were measured at hours 24 and 0 (07:00) using SOMAscan and compared the differences between timepoints for both plasma and CSF. Specifically, we used the SOMAscan assay 1.3K kit, which is a high-scale aptamer-based proteomic platform that measures relative concentrations of about 1300 proteins. All blood and CSF samples were analyzed per manufacturer suggestions. Briefly, we divided 50 µL of sample across three contiguous wells of a SOMAscan assay plate and diluted at 20% for CSF and at three different concentrations (40%, 1%, and 0.005%) for plasma to account for expected endogenous abundance of the proteins of interest. Samples were hybridized into Agilent Surescan MicroArray slides and then scanned at a 5-µm resolution to detect CY3 fluorescence. Gridding and image analysis were done using Agilent Feature Extraction v10.7.3.1. Raw signals were standardized according to SOMAscan standardization procedures as described elsewhere [31, 32].

From among the SOMAscan identified proteins with greater than 2-fold change, two proteins (one CSF and one plasma) were selected for protein expression validation by enzyme-linked immunosorbent assays (ELISA) (Supplementary Figure S1). ELISAs were performed to measure Cystatin F in plasma (Thermo-Fisher) and albumin in CSF (Abcam), according to manufacturer suggestions.

### Protein gene list and bioinformatic analyses

Upregulated and downregulated protein gene lists based on the SOMAscan data were processed through the web interface Metascape [33] for the gene ontology, biological function, and pathway analyses. The Metascape utilizes the well-adopted hypergeometric test to identify statistically enriched ontology terms. Significant terms were defined as those with a minimum count of three genes and an enrichment factor more than 1.5 (i.e. the ratio between the observed counts and the counts expected by chance) at *p* < 0.05 or Benjamini–Hochberg false discovery rate corrected *p* < 0.05. The Metascape also uses Kappa similarity algorithm to absorb most redundancies of biological terms into representative clusters. Clusters were named based on the most statistically significant term within the cluster. Analysis in Metascape provided the top upregulated and downregulated gene ontology clusters with their representative enriched terms for illustration and discussion.

To capture the relationships between terms, we selected the top 15 enriched terms from each of the 20 top cluster terms and rendered them as a network plot visualized with Cytoscape. The terms with the best *p*-values had no more than 15 terms per cluster and no more than 250 terms in total. Circle node color is paired with the enriched terms that represent cluster identity (text written in the same color in the figure) and node size is proportional to the number of input genes included in the selected terms. Terms with a Kappa similarity score > 0.3 are linked by a branch whose thickness represents the similarity score. Finally, we used STRING and BioGrid to reveal enriched protein-protein physical interactions. Protein interaction networks containing between 3 and 500 proteins were included and analyzed through the MCODE algorithm to identify densely connected network components. The three best-scoring terms by *p*-value were retained as a functional description of the corresponding components.

### Statistics

Standardized signal data from the SOMAscan were quantile-normalized and then proceeded to differential expression analysis with the R package limma [34]. Comparison of SOMAscan protein expression value between T24 versus T0 was done through a patient-paired T-test with the Bayesian statistics for microarray studies. The Limma software generates multiple test correction-adjusted *p*-values with Benjamini and Hochberg false discovery rate procedure, while providing nominal *p*-values. Since pathway genes tend to be regulated in groups, restricted multiple test correction-adjusted *p*-values would cost higher false negative rates. Thus, differentially expressed proteins used for pathway enrichment analysis with Metascape were defined as those with *p* < 0.05 in this study.

## Results

### Plasma

#### Acute sleep deprivation decreased the number of upregulated and downregulated biological pathways in plasma.

**Figure 1.**
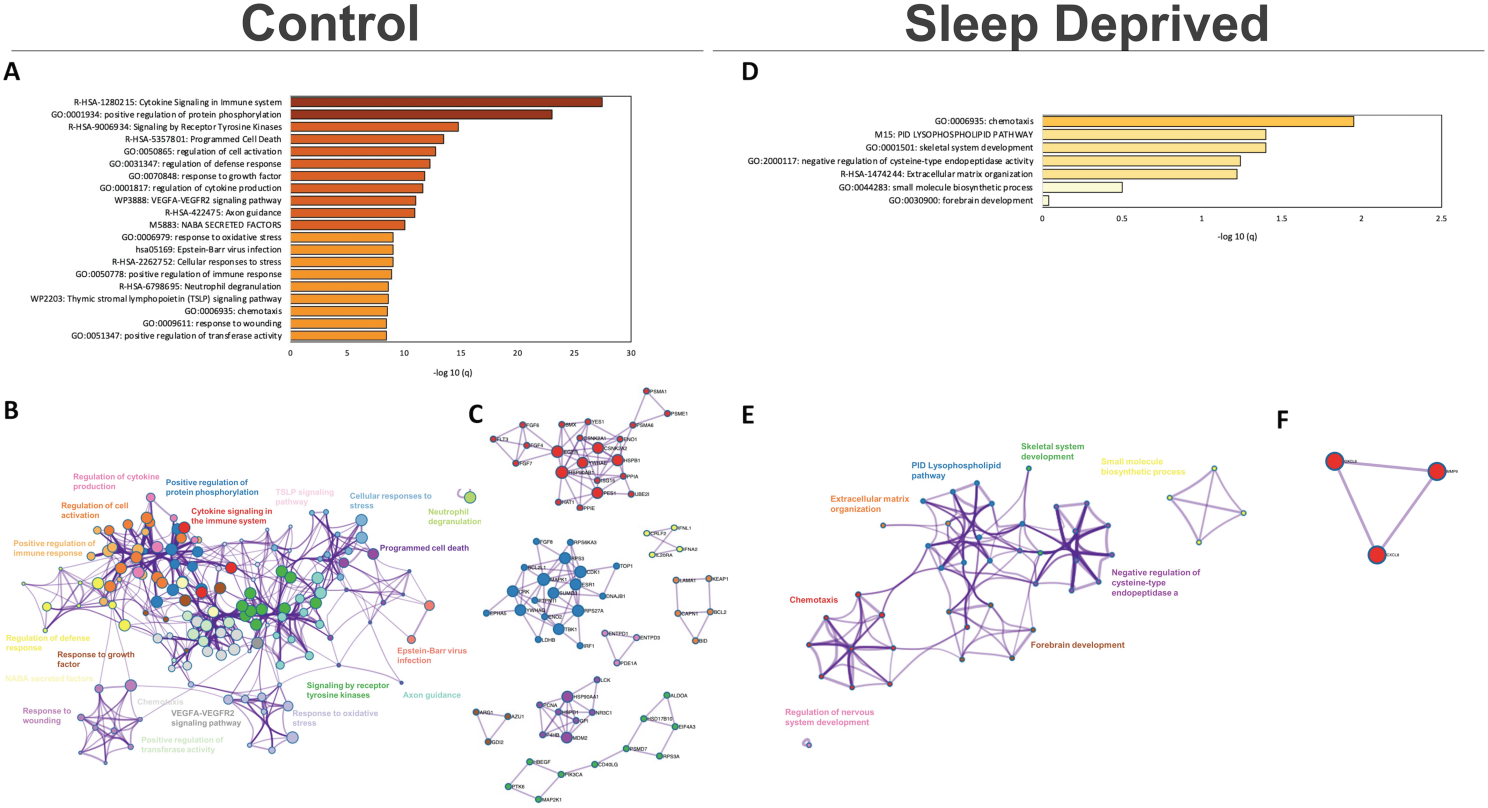
Profile of upregulated biological terms and protein interaction networks in Plasma samples of control and sleep-deprived participants. Bar graph for the top 20 gene ontology terms with their representative enriched term, colored by *q*-value (A,D). Network plot where each node represents the above-enriched terms and is colored by cluster ID where nodes that share the same cluster ID are typically close to each other (B,E). Protein-protein interaction enrichment analysis for the main protein interaction networks (C/F).

To give biological context to the consequences of acute sleep deprivation, we identified upregulated and downregulated biological pathways after control and sleep-deprived conditions. Pathway and process enrichment analysis through Metascape comparing plasma samples from hour 24 to hour 0 found upregulation of biological terms in enriched clusters (Figure 1A, Supplementary Figure S2A for top 100 enriched term clusters, and Supplementary Figure S3A for details on count and *p* and *q*-values) associated to immune response, signaling pathways, promotion of phosphorylation, cell proliferation and connectivity programmed cell death when participants were under normal sleep control conditions. Figure 1B shows cluster similarity. Protein-protein interaction enrichment analysis identified significant protein interaction networks associated with cytokine signaling, interleukin signaling, positive regulation of protein phosphorylation, and tyrosine kinase receptor signaling (Figure 1C, see Supplementary Figure S3B for *p*-value details).

In contrast, the profile of upregulated term clusters of plasma samples from participants after acute sleep deprivation was different, less diverse, less significantly upregulated, and with a smaller number of significant terms associated with the clusters (Figure 1D, see Supplementary Figure S2B for top 100 enriched clusters, and Supplementary Figure S4A for details on count and *p* and *q*-values). Biological terms in upregulated clusters included skeletal system development, extracellular membrane (ECM) organization, and small molecule biosynthetic process. Only the cluster with *chemotaxis* as an enriched term overlapped with control samples (see Supplementary Figure S2, A and B) and had the most significant protein-protein interactions network identified (Figure 1F; see Supplementary Figure S4B for *p*-value details).

**Figure 2.**
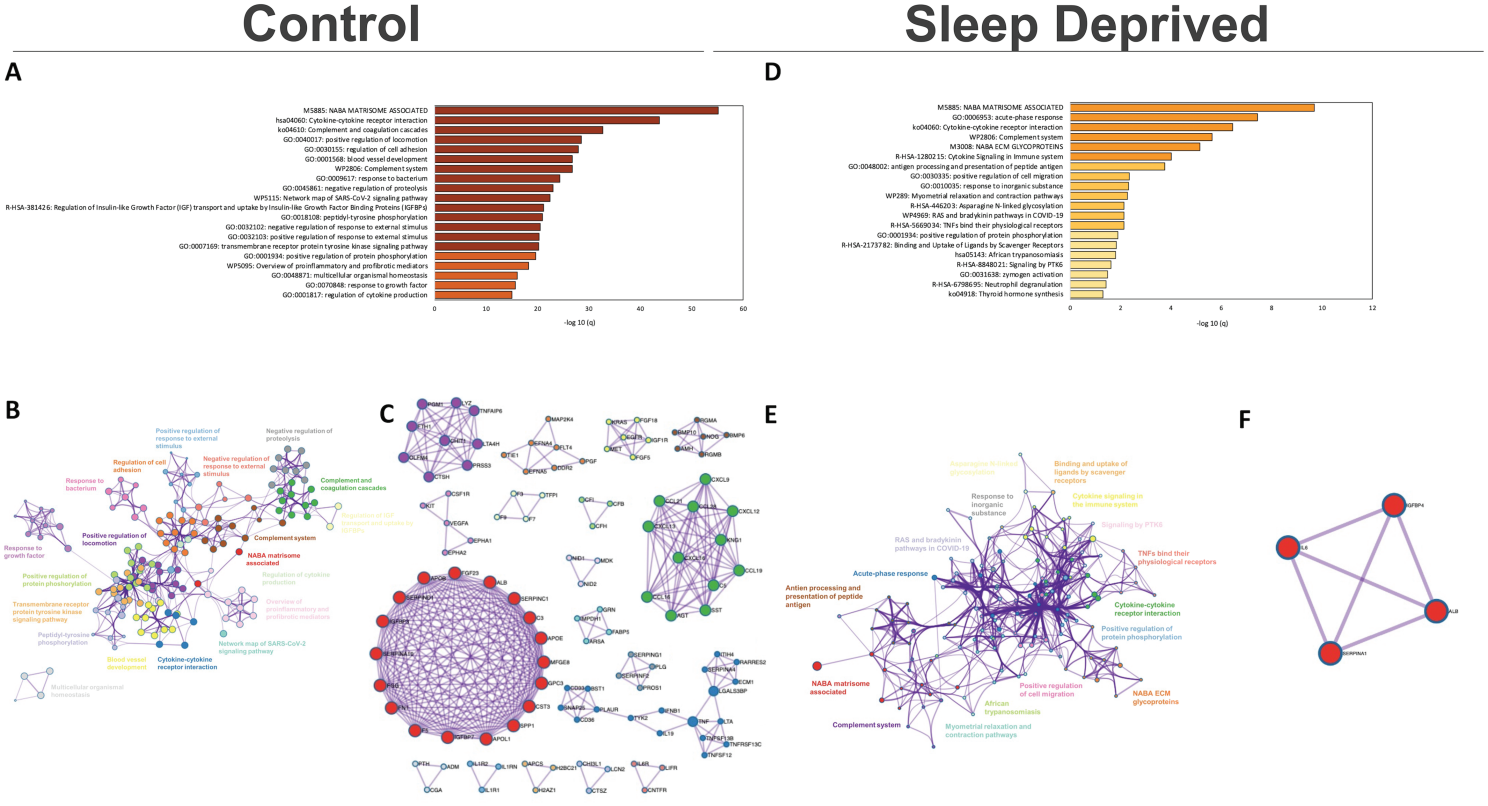
Profile of downregulated biological terms and protein interaction networks in Plasma samples of control and sleep-deprived participants. Bar graph for the top 20 gene ontology terms with their representative enriched term, colored by *q*-value (A,D). Network plot where each node represents the above-enriched terms and is colored by cluster ID where nodes that share the same cluster ID are typically close to each other (B,E). Protein-protein interaction enrichment analysis identifies the main protein interaction networks (C/F).

When comparing hour 24 to hour 0 under normal sleep control conditions, statistically significant downregulated biological terms in enriched clusters associated with NABA matrisome (which consists of ECM proteins and associated proteins), cytokine signaling, complement and coagulation cascades, pro-inflammatory mediators, positive regulation of protein phosphorylation, among others (Figure 2A, see Supplementary Figure S5A for top 100 enriched clusters, and Supplementary Figure S6A for details of count and *p* and *q*-values). Most of the enriched terms in these clusters retained close similarity (Figure 2B). Protein-protein interaction analyses revealed downregulation of densely interconnected protein networks associated with these same biological themes (Figure 2C; see Supplementary Figure S6B for *p*-value details). Following acute sleep deprivation, plasma samples had a reduced profile of downregulated biological themes compared to control. While there was some overlap with control samples for the same pathways mentioned above, sleep loss also caused downregulation of biological clusters associated with the following terms: acute phase response, NABA ECM glycoproteins, positive regulation of cell migration, antigen processing and presentation of peptide antigen, zymogen activation, among others (Figure 2D; see Supplementary Figure S5B for top 100 enriched clusters, and Supplementary Figure S7A for details of count and *p* and *q*-values). The network plot visualization shows selected terms retained similarity with terms of other clusters although there were less enriched terms in the clusters (Figure 2E; see Supplementary Figures S6A and 7A for count comparison). There was a dramatic disruption of protein interaction networks with the only interaction network significantly downregulated being the NABA matrisome associated proteins (Figure 2F; Supplementary Figure S7B).

A subset of terms changed from upregulated to downregulated and vice versa after sleep deprivation (Supplementary Table S1).

#### Acute sleep deprivation effects on plasma protein expression.

**Figure 3.**
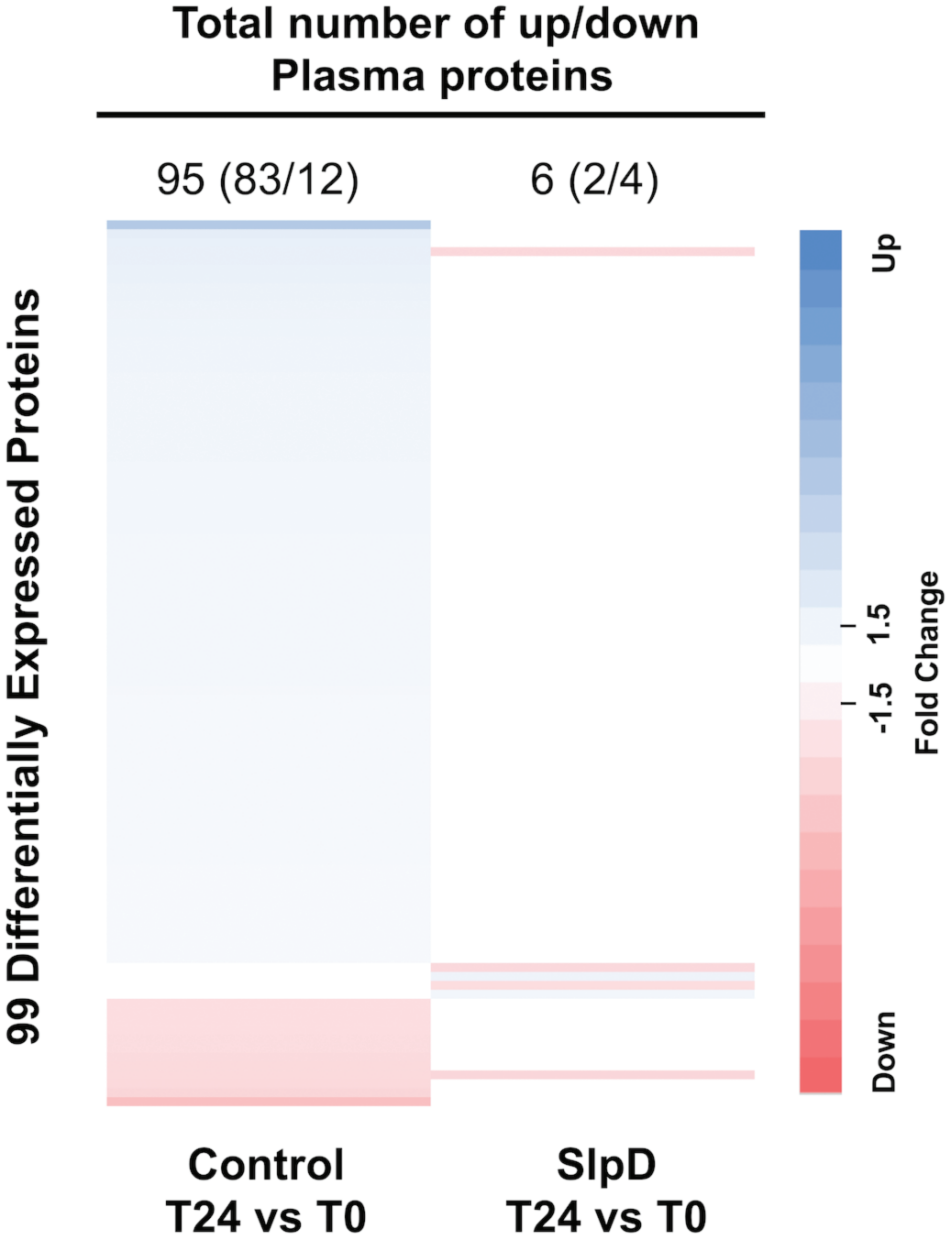
Differentially expressed protein in Plasma samples of control and sleep-deprived participants. Heatmap of differentially expressed proteins in plasma during control and sleep-deprived (SlpD) conditions at T24 versus T0. Proteins are organized by descending value of fold change in control samples. Protein fold change is colored by directionality of ≥1.5-fold change as follows: blue = upregulated, red = downregulated, and white = less than 1.5-fold change in either direction or no significant change. The color gradient represents magnitude of fold change.

There were a total of 486 proteins significantly altered in plasma between control and sleep-deprived conditions. When participants completed the normal sleep control condition, there were a total of 461 differentially expressed proteins (174 upregulated and 287 downregulated) at hour 24 compared to hour 0 (see Supplementary Figure S8). Ninety-five (83 upregulated and 12 downregulated) showed a significant ≥1.5-fold change (Figure 3, see Supplementary Figure S9 for protein, fold change, and *p*-value data). When the same participants underwent the acute overnight sleep deprivation condition, there were only 72 altered proteins (18 were upregulated and 54 downregulated) (see Supplementary Figure S8). Six total proteins (two upregulated and four downregulated) demonstrated a significant ≥1.5-fold change (Figure 3, see Supplementary Figure S9 for protein, fold change, and *p*-value data). Although 99 proteins between the two conditions had > 1.5-fold change, none had significant *p*-values after adjustment for multiple comparisons. All proteins with any fold change and unadjusted *p*-values < 0.05 are in Supplementary Figure S8 and all proteins with > 1.5-fold change and unadjusted *p*-values < 0.05 are shown in Supplementary Figure S9.

Notably, four newly altered proteins (not previously up or downregulated) with fold change ≥1.5 were observed following sleep deprivation: netrin receptor five (UNC5D) and Matrix Metalloproteinase 9 (MMP-9) were upregulated while thyroid stimulating hormone and Cathepsin D were downregulated. Additionally, sleep deprivation reversed protein expression directionality for Ubiquitin (RSP27A), from upregulated in control samples to downregulated following sleep deprivation.

### Cerebrospinal fluid

#### Acute sleep deprivation increased the number of upregulated and downregulated biological pathways in CSF.

**Figure 4.**
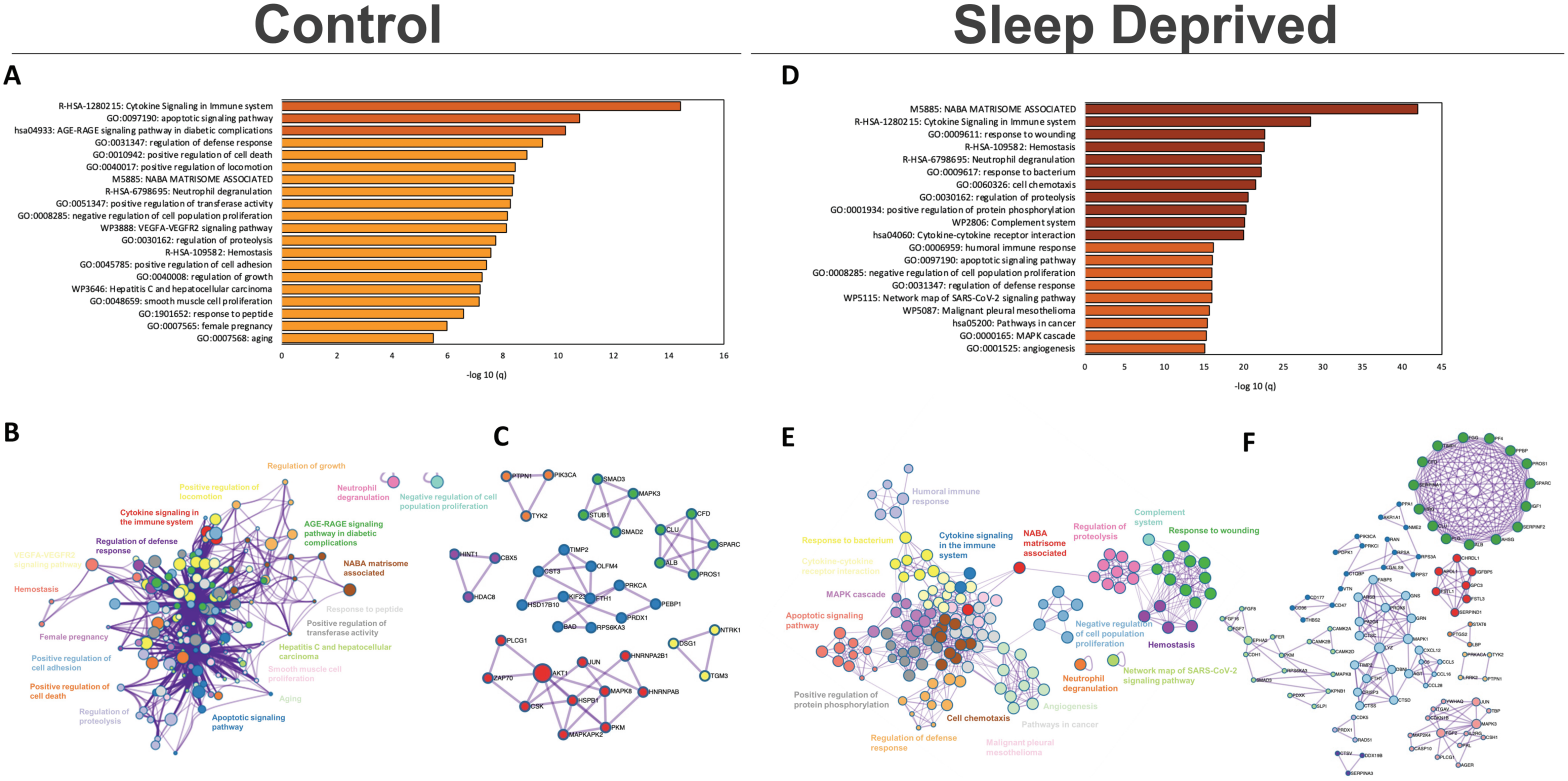
Profile of upregulated biological terms and protein interaction networks in CSF samples of control and sleep-deprived participants. Bar graph for the top 20 gene ontology terms with their representative enriched term, colored by *q*-value (A,D). Network plot where each node represents the above-enriched terms and is colored by cluster ID where nodes that share the same cluster ID are typically close to each other (B,E). Protein-protein interaction enrichment analysis, main protein interaction networks (C/F).

Functional enrichment analysis of CSF samples collected during normal sleep control conditions showed a significant upregulation of several biological terms in enriched clusters that were associated with cytokine signaling in the immune system, apoptotic signaling pathway and positive regulation of cell death, VEGFA-VEGFR signaling pathway, negative regulation of cell proliferation, extracellular membrane, regulation of locomotion, cell adhesion (Figure 4A; see Supplementary Figure S10A for top 100 enriched clusters and Supplementary Figure S11A for details on count and *p* and *q*-values). Terms had close similarity except for two clusters (Figure 4B). Biological terms within clusters of cytokine signaling, apoptotic signaling pathway, and AGE-RAGE signaling pathway had the most significant protein-protein interactions network identified (Figure 4C; see Supplementary Figure S11B for *p*-value details). When participants were sleep-deprived, there was overlap of enriched clusters upregulated during normal sleep control conditions; however, the counts within clusters were higher (Figure 4, D and E, see Supplementary Figure S10B for top 100 enriched clusters, see Supplementary Figure S12A for details on count and *p* and *q*-values). Some of the themes found only in sleep-deprived samples included positive regulation of protein phosphorylation, MAPK cascade, among others. Overall, CSF samples from sleep-deprived participants had a greater number of protein interaction networks, with some of them being part of NABA matrisome, negative regulation of cell population proliferation, response to wounding, cytokine signaling in the immune system, regulation of defense response, neutrophil degranulation, angiogenesis (Figure 4F, see Supplementary Figure S12B for *p*-value details).

**Figure 5.**
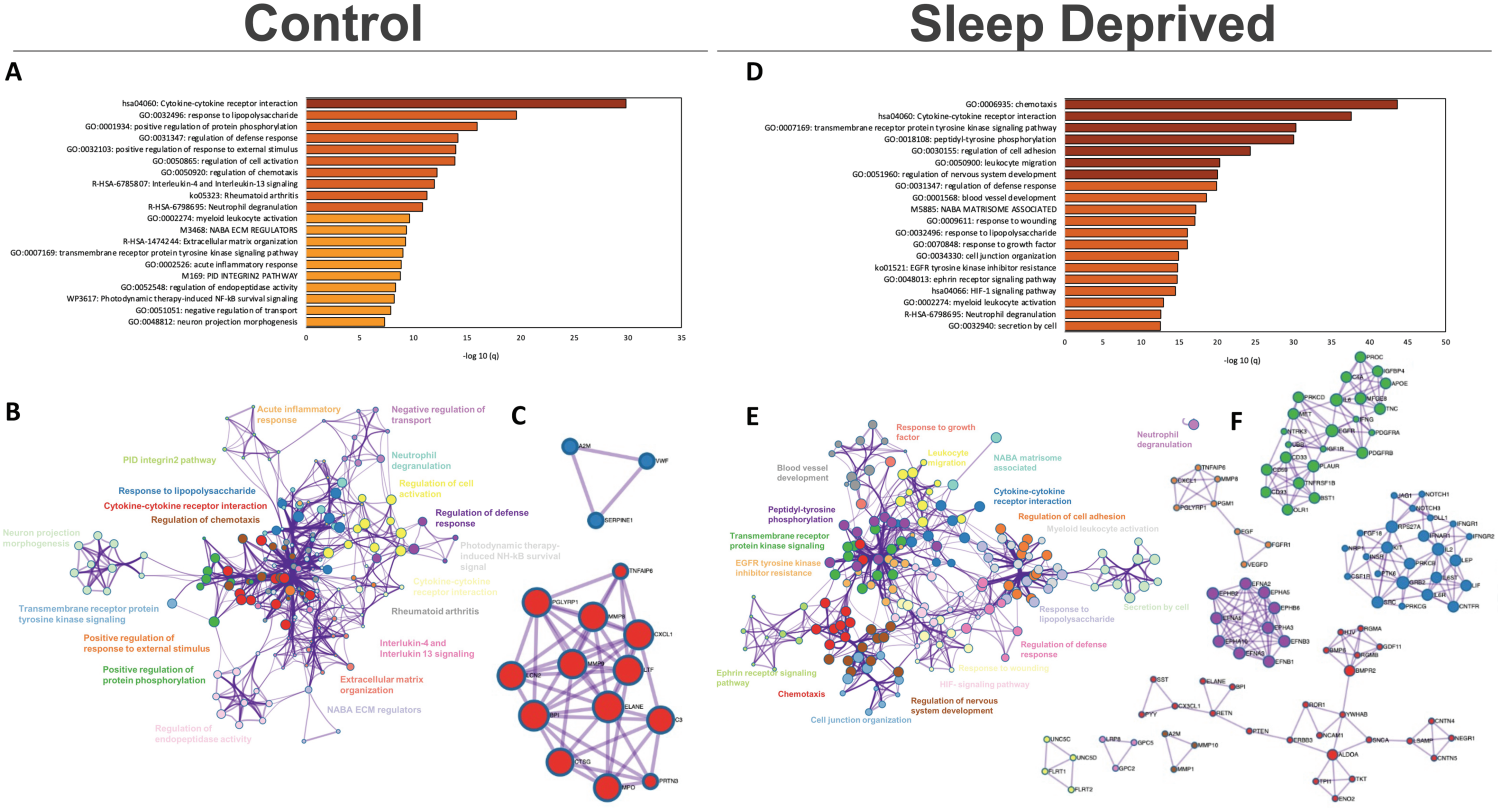
Profile of downregulated biological terms and protein interaction networks in CSF samples of control and sleep-deprived participants. Bar graph for the top 20 gene ontology terms with their representative enriched term, colored by *q*-value (A,D). Network plot where each node represents the above-enriched terms and is colored by cluster ID where nodes that share the same cluster ID are typically close to each other (B,E). Protein-protein interaction enrichment analysis, main protein interaction networks (C/F).

Top downregulated biological terms (Figure 5A; see Supplementary Figure S13A for top 100 enriched clusters and see Supplementary Figure S14A for count and *p* and *q*-values) with close relationship (Figure 5B) under normal sleep control included cytokine-cytokine receptor interaction, response to lipopolysaccharide, chemotaxis, NFKb, cell activation, interleukin pathways and extracellular membrane pathways, and acute inflammatory response. The most significant protein-protein interaction networks were observed within the cytokine-cytokine receptor interaction and response to lipopolysaccharide themes (Figure 5C, Supplementary Figure S14B for *p*-value details). After sleep deprivation, we observed some overlap of enriched clusters with normal sleep control. However, overlapping and distinct biological terms were significantly more downregulated (Figure 5D, see Supplementary Figure S13B for top 100 enriched clusters, see Supplementary Figure S15A for count and *p* and *q*-values) and retained a degree of similarity (Figure 5E) and included regulation of nervous system development, ephrin receptor signaling pathway, regulation of cell adhesion, and response to wounding. There was a greater number of protein-protein interaction networks downregulated following sleep deprivation, and included networks associated with the biological terms of peptidyl-tyrosine phosphorylation (particularly Ephrin signaling), cytokine-cytokine receptor interaction (particularly JAK-STAT signaling pathways), chemotaxis, transmembrane receptor protein kinase signaling, peptidyl-tyrosine phosphorylation, leukocyte migration, and regulation of nervous system development (Figure 5F; see Supplementary Figure S15B for *p*-value details).

A subset of terms changed from upregulated to downregulated and vice versa after sleep deprivation (Supplementary Table S2).

#### Acute sleep deprivation increased differential protein expression in CSF.

**Figure 6.**
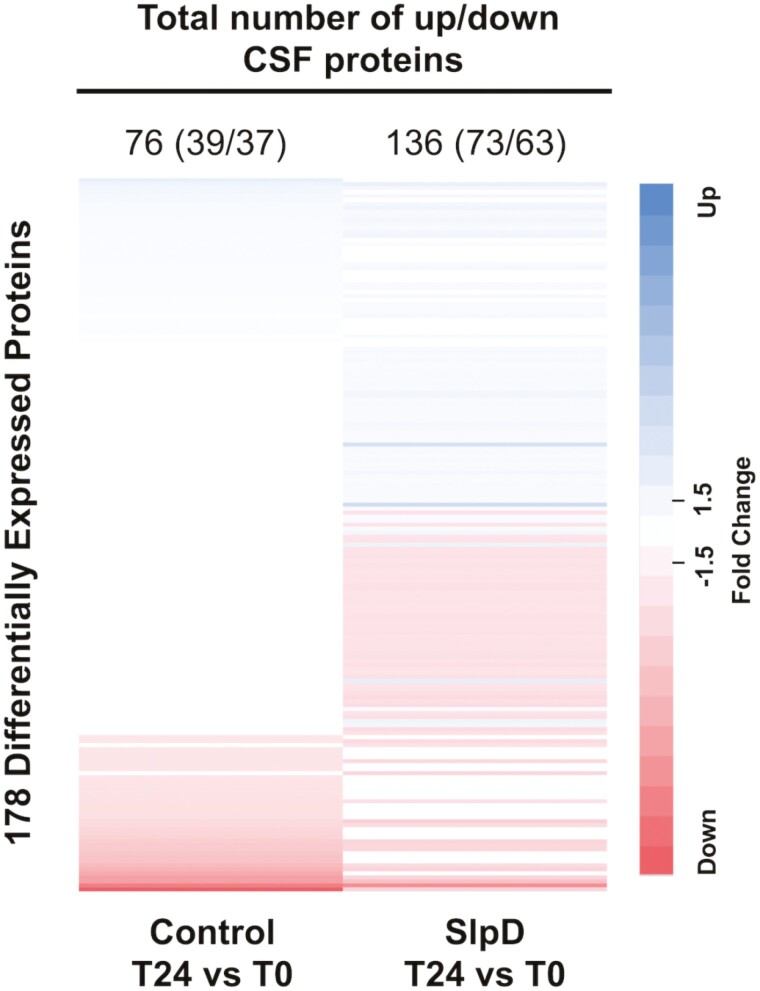
Differentially expressed protein in CSF samples of control and sleep-deprived participants. Heatmap of differentially expressed proteins in CSF during control and sleep-deprived conditions (SlpD) at T24 versus T0. Proteins are organized by descending value of fold change in control samples. Protein fold change is colored by directionality of ≥1.5-fold change as follows: blue = upregulated, red = downregulated, and white = less than 1.5-fold change in either direction or no significant change. The color gradient represents magnitude of fold change.

There were a total of 705 proteins significantly altered in CSF between control and sleep-deprived conditions. Under normal sleep control conditions, participants had a total of 289 differentially expressed proteins (178 upregulated and 111 downregulated) at hour 24 compared to hour 0 (Supplementary Figure S16). Of these differentially expressed proteins, 76 total proteins (39 upregulated and 37 downregulated) demonstrated a significant fold change ≥1.5 (Figure 6, see Supplementary Figure S17 for protein, fold change, and *p*-value data). Following sleep deprivation, there were 571 differentially expressed proteins (310 were upregulated and 261 downregulated) (Supplementary Figure S16) with 136 proteins (73 upregulated and 63 downregulated) demonstrating a significant fold change ≥1.5 (Figure 6, see Supplementary Figure S17 for protein, fold change, and *p*-value data). In total, 178 proteins had ≥1.5-fold change between the two conditions. These numbers include proteins that had a fold change with significant unadjusted and adjusted *p* values.

Notably, 106 proteins (57 upregulated and 49 downregulated) had a significant fold change ≥1.5 and we observed that many of the proteins that were newly altered by sleep deprivation were associated with inflammatory markers and signaling molecules (particularly complement and ephrin receptor signaling) (see Supplementary Table S3).

## Discussion

In this exploratory study, we used parallel biofluid sampling in combination with a high throughput proteomic evaluation to identify the distinct proteomic signatures of plasma and CSF in participants under control (normal) and acutely sleep-deprived conditions. Acute sleep loss dysregulated proteins and pathways implicated in immune response, inflammation, phosphorylation, membrane signaling, cell-cell adhesion, and extracellular membrane organization. We acknowledge that the number of participants is low, the results are limited to 1300 proteins analyzed, and the experiment is not designed to determine causality of changes from sleep deprivation. However, our data can support further large-scale proteomic-based investigations, and can be a resource for studies looking into sleep loss-associated biomarkers and novel pathways that could mediate risk of conditions adversely associated with sustained wakefulness. Ultimately, this data can help guide and support further research to identify new targets for intervention to prevent or delay human diseases such as AD.

### Plasma

Prior researchers have observed tissue-specific reductions in particular sets of plasma genes and proteins following acute sleep deprivation, such as reduction in skeletal muscle protein synthesis [27] and plasma metabolites [35]. Möller-Levet et al. [36] showed that 1 week of sleep restriction led to a global decrease in the number of plasma circadian genes and gene expression amplitude. Here, from the 1300 proteins analyzed through SOMAscan, we observed a reduced number of upregulated and downregulated proteins following a single night of total sleep deprivation when compared to a normal control night. However, none of these proteins were significant after the -values were adjusted for multiple comparisons.

There are several reasons why differential protein expression in plasma was not significant after adjustment. One reason is the study’s small sample size. An alternative but less likely possibility is that the initial and markedly significant changes due to sleep deprivation are first reflected in the brain and CSF and then, due to downstream effects, changes in plasma are seen. Another consideration is that the proteins we analyzed were not affected by acute sleep deprivation at the timepoint analyzed. Overall, the decrease in differential protein expression following sleep deprivation was reflected in the biological process analyses that revealed a significant decrease in the number of upregulated and downregulated enriched biological pathways even after adjustment for multiple comparisons.

Although definitive conclusions cannot be made with the plasma individual protein data, some of the observed protein changes overlapped with changes in biological pathways therefore this data could still inform the design of larger studies. For instance, the pathways “chemotaxis” and “negative regulation of cysteine-type endopeptidase activity” were upregulated during sleep loss. Within these pathways, the protein matrix metalloproteinase 9 (MMP-9), which participates in the degradation of extracellular matrix, was present in the only upregulated protein-protein network and was one of the only two proteins upregulated > 1.5-fold in our samples. MMP-9 is elevated in OSA patients and correlates with OSA severity [37, 38] and associated risk of cardiovascular disease [38, 39]. It has also been associated with vascular remodeling [40, 41], left ventricle remodeling [42], heart failure [43, 44], severity of ischemic stroke [45, 46], and stroke-associated blood-brain barrier breakdown [47]. Additionally, UNC5D protein expression was also upregulated following sleep deprivation. UNC5D is a netrin receptor involved in inflammation, cell adhesion, cell migration, and cell survival, that has been associated with sleep disturbance, sleep chronotype, and sleep apnea [48–50]. While not associated with the observed upregulated biological terms, this protein may also represent a marker of sleep deprivation that could merit evaluation for involvement in sleep loss pathophysiology and clinical pathologies associated with sleep deprivation.

Following acute sleep deprivation, we also saw a decrease in pathways associated with extracellular membrane, protein phosphorylation, inflammatory mediators, zymogen activation, and cytokine and complement cascades. These findings are consistent with prior investigations in animal models, as well as human proteome and transcriptome studies which have also shown an association between sleep disruption and these biological pathways [36, 51–53]. Notably, cathepsin-D was downregulated following sleep deprivation and is also part of the downregulated cluster “antigen processing and presentation of peptide antigen.” This lysosomal enzyme is associated with aging [54], and cardiovascular disease [55, 56]. It is also associated with neurodegenerative conditions [57], and is particularly decreased in patients with Parkinson’s disease [58] and AD [59].

### Cerebrospinal fluid

Although prior studies investigated the normal human CSF proteome [60], this study assessed the effect of acute sleep deprivation on at least 1300 CSF proteins as surrogate of human brain-associated protein alterations following sustained wakefulness. Prior animal research has shown that sleep deprivation causes differential abundance of transcriptome and proteome, targeting select biological processes in a brain region-specific manner [61–63]. Within the proteins measured by SOMAscan, we observed that acute sleep deprivation led to an increase in the number of upregulated and downregulated proteins in the CSF when compared to normal control sleep, and many of these proteins were not previously seen altered in the control night. Many of these protein changes overlapped with changes in biological pathways as discussed below. This study is unable to establish if the CSF proteomic changes we observed directly reflect changes in the brain parenchyma or are due to other reasons such as decreased CSF clearance following sleep deprivation [64], and further research is needed to explore this relationship. Future studies are also needed to examine the implications of these protein changes following acute sleep deprivation (i.e. whether changes are involved with maintaining wakefulness, increasing functionality following sleep deprivation [65], or increasing sleep drive [66]).

Our pathway analyses in Metascape revealed that acute sleep deprivation altered biological pathways associated with inflammation and cell signaling, among others. The biological themes detected were mostly newly altered and the overall biological fields aligned with prior research [12, 61, 67]. Among the newly upregulated themes following sleep deprivation was the increase in MAPK cascades which are known to mediate cell division, differentiation, inflammation, and cell death, and whose upregulation has been associated with cerebrovascular accidents [68], multiple sclerosis [69], and neurodegenerative diseases [70, 71]. Thus, upregulation of MAPK pathway in CSF of sleep-deprived participants could have detrimental effects on baseline physiology and increase the risk or exacerbate important medical conditions. Additionally, we observed an upregulation of the positive regulation of protein phosphorylation theme, which was downregulated in control samples. Sleep deprivation can cause increased protein phosphorylation that dissipates with sleep [19, 72] which could be relevant to neurodegenerative conditions such as AD [73] where, for example, a sleep-deprived state can drive phosphorylation of tau and increase risk of AD development [21]. Alterations in sleep–wake cycle and circadian genes can lead to a loss of phosphorylation cycles, which are typically needed for housekeeping processes such as transport and scaffolding [74, 75]. Therefore, the observed increase in the positive regulation of protein phosphorylation theme could imply critical effects of sleep deprivation on cellular function during health and disease.

Interestingly, within the positive regulation of protein phosphorylation theme, the serine/threonine-protein kinase WNK3 was upregulated more than 1.5-fold. WNK3 was recently implicated in circadian rhythm regulation in association with the canonical clock protein Per-1 in the SCN of rats [76], an important role given prior research suggests that regulation of circadian rhythm in the setting of sustained wakefulness could promote sleep pressure and recovery sleep [76, 77]. We also observed an upregulation of the formation of fibrin clot theme, and within this theme fibrinogen and other inflammatory markers were upregulated more than 1.5-fold, keeping with prior evidence associating poor sleep and neuroinflammation [8, 78, 79]. This increase in neuroinflammation from poor sleep could be associated with other pathologies such as blood-brain barrier (BBB) disruption [80], cognitive impairment, and neurodegeneration [8, 22, 78, 81]. These protein changes could support mechanisms by which lack of sleep increases risk of the above-mentioned conditions.

This study revealed several downregulated themes in CSF mainly associated with cell signaling and cell adhesion such as ephrin receptor signaling pathway, JAK-STAT signaling pathways, protein tyrosine kinase signaling, and cytokine receptor interaction. Within the downregulated ephrin receptor signaling pathway, several Ephrin proteins were decreased more than 1.5-fold compared with control. Ephrin proteins have been implicated in key neural roles such as cell communication, memory formation, synaptic transmission, and plasticity, as well as neurodegenerative diseases such as AD (e.g. EphB2 and Eph A4) [82]. There was also a decrease in receptors such as BDNF/NT3 and while BDNF has been associated with sleep regulation [83], decreases in BDNF and BDNF/NT3 receptors have been associated with neurodegenerative conditions [84–86]. Our analyses also showed a downregulation of themes associated with regulation of cell adhesion, cell junction organization, and leukocyte migration. This was further supported by decreases in proteins associated with these themes. For instance, Contactin-4, Layilin, Amphoterin-induced protein 2, Endothelial cell-selective adhesion, and Neural cell adhesion molecule L1 have been associated with BBB and early dysregulation could jeopardize BBB integrity which has been previously observed in animal brains after chronic sleep loss [87], REM sleep restriction [88], and linked to neuroinflammation [80].

A significant strength of this study is the parallel sampling of plasma, an easily accessible fluid, and CSF, a fluid that needs a more invasive mode of sampling. This method allows simultaneous inspection and comparison of central and systemic experimental effects that have been previously used in the context of neurological diseases such as AD [89], multiple sclerosis [90], and amyotrophic lateral sclerosis [91] with the aim of identifying surrogate markers of disease in plasma to promote less invasive testing. The implications of the opposite effects of acute sleep deprivation on biological pathways in plasma and CSF are unclear. These changes could be due to differences in tissue response to sleep deprivation, decreased clearance of CSF proteins following sleep deprivation [23], or the biofluids could be influencing each other through mechanisms such as compromised BBB integrity [92]. Analyzing samples across timepoints could help clarify this relationship. Further studies are also needed to determine the underlying mechanisms of these protein changes, (i.e. loss of circadian expression, decreased transcription or translation, epigenomic changes, increased targeted protein degradation), and which of these changes are secondary to sustained wakefulness or a consequence of it.

## Conclusion

This paired analysis provides an overview of biological pathways and proteomic dysregulation in plasma and CSF due to acute sleep deprivation. Our findings are hypothesis-generating, supporting further investigation into normal sleep and sleep deprivation pathophysiology. Further studies are needed to determine if these changes are sustained, clinically relevant, and if they could represent promising targets for future sleep research.

## Limitations

The study conclusions are limited due to the sample size of only five participants, rendering it underpowered to make definitive claims about the observed changes. Our methodological approaches may also limit our findings in that SOMAscan platform analyzes 1300 proteins and the Metascape library evaluates protein gene lists within the confines of previously documented biological terms that have been added to the library. However, as an exploratory study, our data supports prior findings in human and animal models and can in turn support larger proteomic studies. It is unclear whether enforced sleep deprivation shares mechanistic pathophysiology with spontaneous wakefulness in conditions such as insomnia, and hypersomnia. However, our findings suggest potential detrimental effects that may arise from insufficient sleep and sleep loss. Furthermore, our findings reflect acute sleep deprivation changes without accounting for brain region-specific or peripheral tissue-specific changes. Further studies are needed to determine if these changes persist, as prior studies have shown that the proteome of acutely sleep-deprived participants is distinguishable from chronically deprived [93], and recovery sleep can revert some of the changes observed in acute sleep deprivation [61, 94].

## Supplementary Material

zpad047_suppl_Supplementary_Figures_1-17_Tables_1-3

## Data Availability

The data associated with this study was deposited to the NCBI GEO database with accession number GSE244817.
