# Supplementary material for: Plasma and cerebrospinal fluid proteomic signatures of acutely sleep-deprived humans: an exploratory study
Source: Sleep Adv. 2023 Nov 14;4(1):zpad047. doi: 10.1093/sleepadvances/zpad047 (PMC10691441; doi:10.1093/sleepadvances/zpad047)
Supplement: zpad047_suppl_Supplementary_Figures_1-17_Tables_1-3 [file zpad047_suppl_supplementary_figures_1-17_tables_1-3.pdf]

SUPPLEMENTARY MATERIALS

Plasma and CSF Proteomic Signatures of Acutely Sleep-Deprived Humans: An Exploratory Study

Ana Vaquer-Alicea, MD, PhD[1]; Jinsheng Yu, MD, PhD[2]; Haiyan Liu, MD, MS[1]; Brendan P. Lucey, MD, MSCI[1*]

[1]Department of Neurology, Washington University School of Medicine, St Louis, MO USA; [2]Department of Genetics, Washington University School of Medicine, St Louis, MO USA

*Corresponding author:

Brendan P. Lucey

Washington University School of Medicine

Campus Box 8111

660 South Euclid Avenue

St Louis, MO USA 63108

Office: 314-747-3805

Fax: 314-747-3813

Email: luceyb@wustl.edu

Supplementary Table 1: Biological themes of plasma samples that switched expression directionality between control and sleep deprived subject samples

Switch from CTL down to SD up	Switch from CTL up to SD down
Regulation of nervous system development	Cytokine signaling in the immune system
	Positive regulation of protein phosphorylation
	Neutrophil degranulation
	Aging
	Natural killer cell mediated cytotoxicity

CTL= control; SD= sleep deprived

Supplementary Table 2: Biological themes of CSF samples that switched expression directionality between control and sleep deprived subject samples

Switch from CTL down to SD up	Switch from CTL up to SD down
Positive regulation of protein phosphorylation	Positive regulation of cell death
Interlukin 4 and Interlukin 13 signaling	Spinal cord injury
reg of IGF transport and uptake by IGFBP	IL-18 signaling pathway
PID P53 downstream pathway	Senescence and autophagy in cancer
Formation of fibrin clot	Regulation of vesicle mediated transport
	Cell junction organization
	VEGFA-VEGFR2 signaling pathway

CTL= control; SD= sleep deprived

Supplementary Table 3: CSF proteins newly altered by acute sleep deprivation with >1.5 fold change

Protein	Fold Change	adj. p-value
D-dimer	4.635581	0.002957
Fibrinogen gamma chain	4.091564	0.003271
Fibrinogen	2.812167	0.008946
Pulmonary surfactant-associated protein D	2.618119	0.034517
Neutrophil-activating peptide 2	2.201515	0.000208
GTP-binding nuclear protein Ran	2.078548	0.002059
Melanoma-derived growth regulatory protein	2.034388	1.54E-05
Connective tissue-activating peptide III	2.008190	0.000292
Sonic hedgehog protein	2.007332	0.001844
CD177 antigen	2.006553	5.5E-06
Pyruvate kinase PKM	1.998396	0.005194
Aflatoxin B1 aldehyde reductase member 2	1.974716	0.00471
Creatine kinase M-type:Creatine kinase B-type heterodimer	1.922789	0.031133
Matrilysin	1.918662	4.66E-05
N-acetyl-D-glucosamine kinase	1.872845	0.006252
Glypican-3	1.863807	0.000138
Tyrosine-protein kinase CSK	1.733274	0.029078
Mitogen-activated protein kinase 3	1.714734	0.011005
Eukaryotic translation initiation factor 4H	1.706500	0.007892
Importin subunit beta-1	1.690970	0.018986
Dermatopontin	1.685898	5.5E-06
Protein NOV homolog	1.663953	1.54E-05
Lithostathine-1-alpha	1.660651	2.14E-05
C-C motif chemokine 16	1.652432	2.46E-05
Proteasome activator complex subunit 1	1.625039	0.001571
Serine/threonine-protein kinase WNK3	1.618687	0.014072
Trypsin-2	1.616767	0.001808
Trypsin-1	1.610519	0.003371
Platelet factor 4	1.602803	0.003723
Prolactin	1.597168	0.000731
Alpha-1-antitrypsin	1.591531	0.001807
Fibroblast growth factor 7	1.590499	0.001808
C-C motif chemokine 5	1.589510	0.000869
Granzyme A	1.587400	0.000199
Angiostatin	1.586988	0.000233
C-C motif chemokine 14	1.583711	0.00019
C-reactive protein	1.577503	0.000417
Tumor necrosis factor receptor superfamily member 17	1.577107	4.93E-06
Regenerating islet-derived protein 4	1.572970	9.58E-05
Cadherin-1	1.562415	2.46E-05
Immunoglobulin A	1.561147	0.001969
Antileukoproteinase	1.558895	2.14E-05
Peroxiredoxin-1	1.552833	0.02003
SUMO-conjugating enzyme UBC9	1.551006	0.003432
Vacuolar protein sorting-associated protein VTA1 homolog	1.547681	0.006223
cAMP-dependent protein kinase catalytic subunit alpha	1.544743	0.010611
C-C motif chemokine 15	1.527827	9.5E-06
Serum albumin	1.527672	0.000932
Inorganic pyrophosphatase	1.518742	0.033352
Nucleoside diphosphate kinase B	1.496598	0.022682
Cathepsin L2	1.491658	0.00627
Proliferation-associated protein 2G4	1.481324	0.024226
Alpha-2-HS-glycoprotein	1.478507	0.000454
C5a anaphylatoxin	1.471378	0.030775
Alpha-2-antiplasmin	1.468639	0.000875
Interleukin-1 receptor type 2	1.467616	0.0006
Eukaryotic translation initiation factor 5A-1	1.464241	0.024226
Kin of IRRE-like protein 3	-1.451620	0.006252
Interferon gamma	-1.465507	4.06E-05
Estradiol 17-beta-dehydrogenase 1	-1.469130	0.049831
Malate dehydrogenase, cytoplasmic	-1.471102	4.66E-05
Delta-like protein 1	-1.474977	0.000197
Syntaxin-1A	-1.481446	0.005715
Platelet-derived growth factor receptor alpha	-1.487146	0.000434
RGM domain family member B	-1.494987	5.5E-06
Lactadherin	-1.496522	0.00094
Bone morphogenetic protein receptor type-2	-1.508688	0.004325
Endothelial cell-selective adhesion molecule	-1.520300	9.66E-05
Ephrin type-B receptor 2	-1.528541	0.00013
Neural cell adhesion molecule L1	-1.528831	0.00013
Urokinase plasminogen activator surface receptor	-1.529156	4.66E-05
CD166 antigen	-1.530874	0.000269
PILR alpha-associated neural protein	-1.535675	0.000158
Basigin	-1.541431	0.000168
Tyrosine-protein kinase receptor TYRO3	-1.542172	0.000197
Amphoterin-induced protein 2	-1.544206	0.001831
NT-3 growth factor receptor	-1.547391	0.000124
Contactin-5	-1.558823	0.00669
Hepatocyte growth factor receptor	-1.564093	6.79E-05
Contactin-4	-1.586403	5.23E-05
OX-2 membrane glycoprotein	-1.588239	0.000197
Neural cell adhesion molecule 1, 120 kDa isoform	-1.591679	2.14E-05
Layilin	-1.594460	2.46E-05
Ephrin-A3	-1.602100	4.66E-05
Metalloproteinase inhibitor 3	-1.631293	9.5E-06
Glutathione S-transferase A3	-1.631930	0.031802
Ubiquitin+1, truncated mutation for UbB	-1.636731	2.14E-05
Histone H1.2	-1.638497	0.034659
Acidic leucine-rich nuclear phosphoprotein 32 family member B	-1.645387	0.001539
Low affinity immunoglobulin epsilon Fc receptor	-1.655725	1.54E-05
BDNF/NT-3 growth factors receptor	-1.660820	9.83E-05
Low-density lipoprotein receptor-related protein 8	-1.670986	1.13E-05
Chromogranin-A	-1.724173	0.000121
Ephrin-B3	-1.767751	4.45E-05
Ephrin type-A receptor 10	-1.861275	5.06E-05
Ephrin type-A receptor 5	-1.874977	2.46E-05
SLIT and NTRK-like protein 5	-1.910978	2.31E-05
GDNF family receptor alpha-2	-1.926497	2.14E-05
Hemojuvelin	-1.982324	2.46E-05
Intercellular adhesion molecule 5	-1.985274	2.07E-05
Ephrin type-B receptor 6	-2.193880	2.14E-05
Immunoglobulin superfamily containing leucine-rich repeat protein 2	-2.203472	2.14E-05
Netrin receptor UNC5D	-2.310046	4.93E-06
Neurexin-1-beta	-2.346457	2.14E-05
Neuroblastoma suppressor of tumorigenicity 1	-2.475061	2.17E-05
Complement C1r subcomponent	-2.636934	0.046541

A

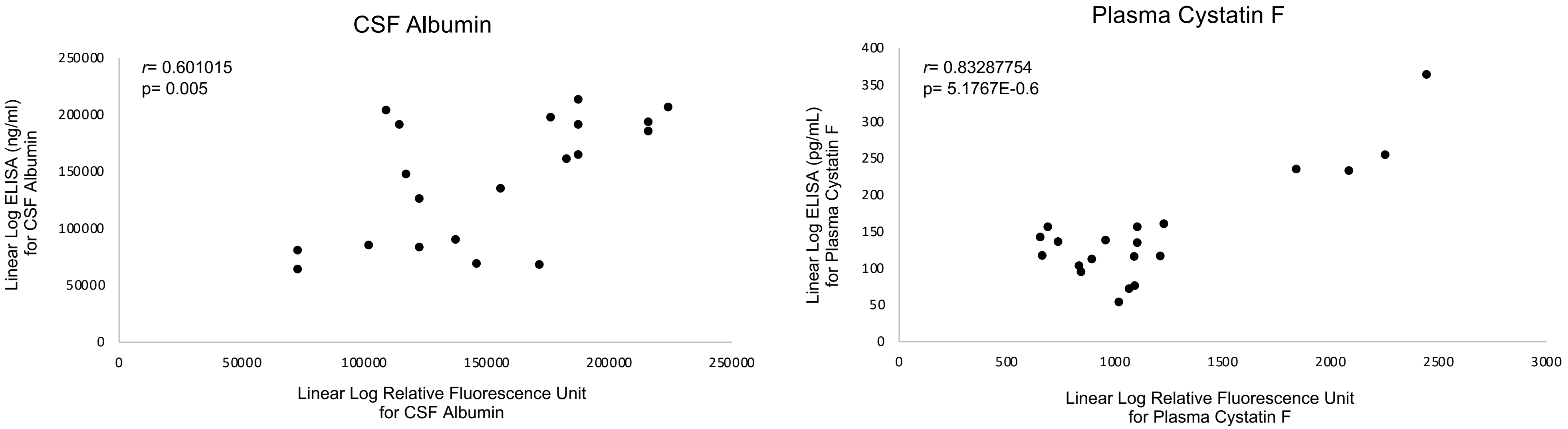

Supplementary Figure 1. SOMAscan protein expression accuracy validation data. Correlation between CSF Albumin and Plasma Cystatin F as measured by ELISA and SOMAscan. Samples are T0 and T24 of subjects in Control (N=5) and Sleep deprived conditions (N=5). All samples were run in duplicate. As shown on graphs, the Pearson correlation coefficients were r=0.799 (p<0.02) for CSF albumin, r=0.865 (p<0.001) for plasma Cystatin F (A).

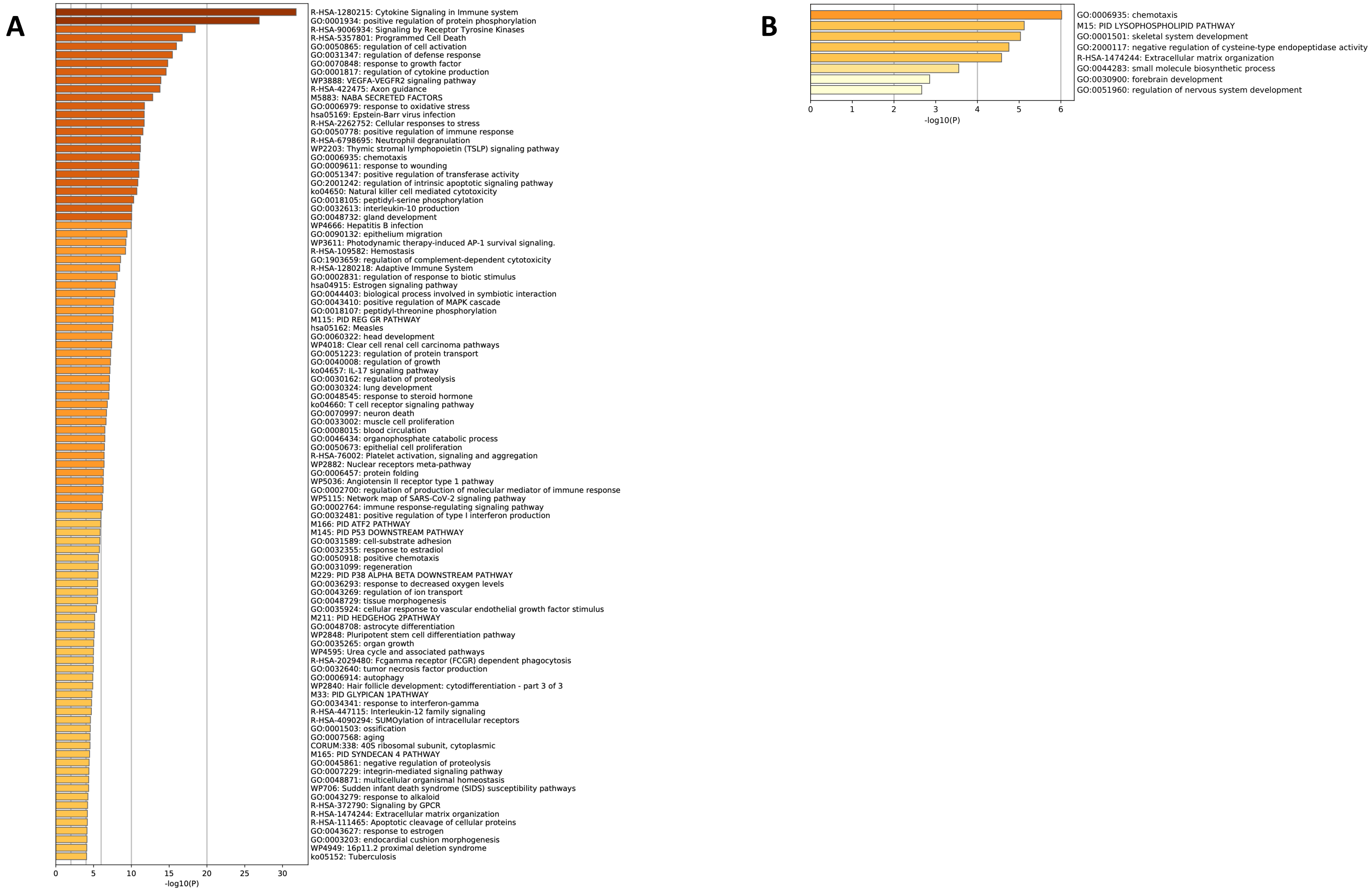

Supplementary Figure 2. Bar graph of top 100 upregulated enriched terms across input gene list in plasma samples of control (A) and acutely sleep deprived subjects (B) at hour 24 vs hour 0. Colored by p-value.

A

GO	Category	Description	Count	%	Log10(P)	Log10(q)
R-HSA-1280215	Reactome Gene Sets	Cytokine Signaling in Immune system	44	25.29	-31.80	-27.44
GO:0001934	GO Biological Processes	positive regulation of protein phosphorylation	40	22.99	-26.91	-23.03
R-HSA-9006934	Reactome Gene Sets	Signaling by Receptor Tyrosine Kinases	28	16.09	-18.46	-14.79
R-HSA-5357801	Reactome Gene Sets	Programmed Cell Death	19	10.92	-16.73	-13.49
GO:0050865	GO Biological Processes	regulation of cell activation	28	16.09	-15.97	-12.78
GO:0031347	GO Biological Processes	regulation of defense response	27	15.52	-15.43	-12.30
GO:0070848	GO Biological Processes	response to growth factor	28	16.09	-14.81	-11.83
GO:0001817	GO Biological Processes	regulation of cytokine production	29	16.67	-14.60	-11.65
WP3888	WikiPathways	VEGFA-VEGFR2 signaling pathway	22	12.64	-13.91	-11.04
R-HSA-422475	Reactome Gene Sets	Axon guidance	24	13.79	-13.79	-10.95
M5883	Canonical Pathways	NABA SECRETED FACTORS	19	10.92	-12.83	-10.09
GO:0006979	GO Biological Processes	response to oxidative stress	20	11.49	-11.73	-9.05
hsa05169	KEGG Pathway	Epstein-Barr virus infection	17	9.77	-11.70	-9.03
R-HSA-2262752	Reactome Gene Sets	Cellular responses to stress	25	14.37	-11.70	-9.03
GO:0050778	GO Biological Processes	positive regulation of immune response	22	12.64	-11.53	-8.89
R-HSA-6798695	Reactome Gene Sets	Neutrophil degranulation	20	11.49	-11.20	-8.60
WP2203	WikiPathways	Thymic stromal lymphopoietin (TSLP) signaling pathway	9	5.17	-11.20	-8.60
GO:0006935	GO Biological Processes	chemotaxis	22	12.64	-11.11	-8.53
GO:0009611	GO Biological Processes	response to wounding	21	12.07	-11.00	-8.45
GO:0051347	GO Biological Processes	positive regulation of transferase activity	21	12.07	-11.00	-8.45

B

Color	MCODE	GO	Description	Log10(P)
	MCODE_1	R-HSA-1257604	PIP3 activates AKT signaling	-14.8
	MCODE_1	R-HSA-9006925	Intracellular signaling by second messengers	-14.2
	MCODE_1	R-HSA-5683057	MAPK family signaling cascades	-12.1
	MCODE_2	R-HSA-5683057	MAPK family signaling cascades	-9.2
	MCODE_2	R-HSA-5654732	Negative regulation of FGFR3 signaling	-8.6
	MCODE_2	R-HSA-5654733	Negative regulation of FGFR4 signaling	-8.5
	MCODE_3	R-HSA-422475	Axon guidance	-6.3
	MCODE_3	R-HSA-1227986	Signaling by ERBB2	-6.3
	MCODE_3	R-HSA-9675108	Nervous system development	-6.2
	MCODE_4	GO:0048545	response to steroid hormone	-8.0
	MCODE_4	GO:0043281	regulation of cysteine-type endopeptidase activity involved in apoptotic process	-6.8
	MCODE_4	GO:2000116	regulation of cysteine-type endopeptidase activity	-6.6
	MCODE_5	GO:0010506	regulation of autophagy	-7.2
	MCODE_5	WP4658	Small cell lung cancer	-6.5
	MCODE_5	GO:0006914	autophagy	-6.3
	MCODE_6	ko04630	Jak-STAT signaling pathway	-9.2
	MCODE_6	GO:0032674	regulation of interleukin-5 production	-9.1
	MCODE_6	GO:0032634	interleukin-5 production	-9.1
	MCODE_7	R-HSA-6798695	Neutrophil degranulation	-5.4
	MCODE_8	GO:1901292	nucleoside phosphate catabolic process	-7.7
	MCODE_8	GO:0046434	organophosphate catabolic process	-6.9
	MCODE_8	ko00230	Purine metabolism	-6.7

Supplementary Figure 3. Metascape analyses of Plasma samples from control subjects hour 24 vs hour 0 showing upregulated biological pathways and protein interaction networks. Top 20 clusters with their representative enriched terms. "Count" is the number of provided genes within terms. "%" is the percentage of all of the user-provided genes that are found in the given ontology term. "Log10(P)" = p-value in log base 10. "Log10(q)" = multi-test adjusted p-value in log base 10 (A). MCODE components identified in the gene lists of the protein-protein interaction networks (B).

A

GO	Category	Description	Count	%	Log10(P)	Log10(q)
GO:0006935	GO Biological Processes	chemotaxis	6	33.33	-6.02	-1.95
M15	Canonical Pathways	PID LYSOPHOSPHOLIPID PATHWAY	3	16.67	-5.12	-1.40
GO:0001501	GO Biological Processes	skeletal system development	5	27.78	-5.03	-1.40
GO:2000117	GO Biological Processes	negative regulation of cysteine-type endopeptidase activity	3	16.67	-4.76	-1.24
R-HSA-1474244	Reactome Gene Sets	Extracellular matrix organization	4	22.22	-4.58	-1.22
GO:0044283	GO Biological Processes	small molecule biosynthetic process	4	22.22	-3.55	-0.50
GO:0030900	GO Biological Processes	forebrain development	3	16.67	-2.86	-0.04
GO:0051960	GO Biological Processes	regulation of nervous system development	3	16.67	-2.66	0.00

B

Color	MCODE	GO	Description	Log10(P)
	MCODE_1	ko04657	IL-17 signaling pathway	-7.6
	MCODE_1	hsa04657	IL-17 signaling pathway	-7.5
	MCODE_1	WP5095	Overview of proinflammatory and profibrotic mediators	-7.1

Supplementary Figure 4. Metascape analyses of Plasma samples from sleep deprived subjects hour 24 vs hour 0 showing upregulated biological pathways and protein interaction networks. Top 20 clusters with their representative enriched terms (A). MCODE components identified in the gene lists of the protein-protein interaction networks (B). See supplementary figure 3 caption for explanation of “count” “%”, “Log10(P)”, and “Log10(q)" .

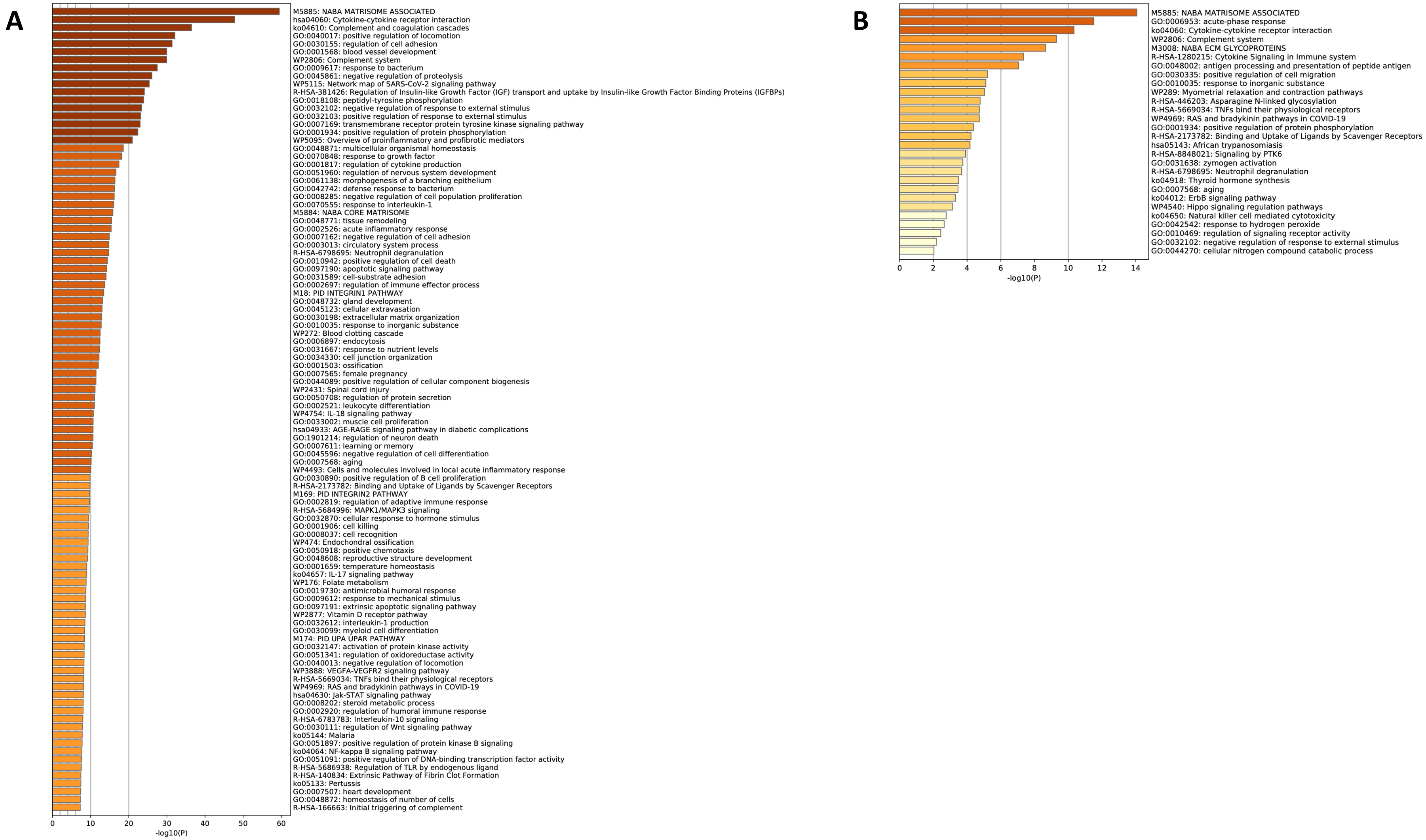

Supplementary Figure 5. Bar graph of top 100 downregulated enriched terms across input gene list in plasma samples of control (A) and acutely sleep deprived subjects (B) at hour 24 vs hour 0. Colored by p-value.

A

GO	Category	Description	Count	%	Log10(P)	Log10(q)
M5885	Canonical Pathways	NABA MATRISOME ASSOCIATED	79	28.21	-59.47	-55.11
hsa04060	KEGG Pathway	Cytokine-cytokine receptor interaction	52	18.57	-47.75	-43.69
ko04610	KEGG Pathway	Complement and coagulation cascades	28	10.00	-36.44	-32.68
GO:0040017	GO Biological Processes	positive regulation of locomotion	50	17.86	-32.07	-28.56
GO:0030155	GO Biological Processes	regulation of cell adhesion	54	19.29	-31.35	-27.94
GO:0001568	GO Biological Processes	blood vessel development	53	18.93	-29.95	-26.74
WP2806	WikiPathways	Complement system	26	9.29	-29.93	-26.74
GO:0009617	GO Biological Processes	response to bacterium	50	17.86	-27.46	-24.38
GO:0045861	GO Biological Processes	negative regulation of proteolysis	36	12.86	-26.05	-23.04
WP5115	WikiPathways	Network map of SARS-CoV-2 signaling pathway	30	10.71	-25.35	-22.41
R-HSA-381426	Reactome Gene Sets	Regulation of Insulin-like Growth Factor (IGF) transport and uptake by Insulin-like Growth Factor Binding Proteins (IGFBPs)	24	8.57	-24.09	-21.19
GO:0018108	GO Biological Processes	peptidyl-tyrosine phosphorylation	35	12.50	-23.90	-21.01
GO:0032102	GO Biological Processes	negative regulation of response to external stimulus	36	12.86	-23.34	-20.54
GO:0032103	GO Biological Processes	positive regulation of response to external stimulus	36	12.86	-23.10	-20.31
GO:0007169	GO Biological Processes	transmembrane receptor protein tyrosine kinase signaling pathway	42	15.00	-22.97	-20.21
GO:0001934	GO Biological Processes	positive regulation of protein phosphorylation	44	15.71	-22.38	-19.66
WP5095	WikiPathways	Overview of proinflammatory and profibrotic mediators	22	7.86	-20.92	-18.30
GO:0048871	GO Biological Processes	multicellular organismal homeostasis	34	12.14	-18.56	-16.09
GO:0070848	GO Biological Processes	response to growth factor	39	13.93	-18.12	-15.68
GO:0001817	GO Biological Processes	regulation of cytokine production	40	14.29	-17.43	-15.02

B

Color	MCODE	GO	Description	Log10(P)
	MCODE_1	R-HSA-8957275	Post-translational protein phosphorylation	-44.7
	MCODE_1	R-HSA-381426	Regulation of Insulin-like Growth Factor (IGF) transport and uptake by Insulin-like Growth Factor Binding Proteins (IGFBPs)	-43.5
	MCODE_1	GO:0007596	blood coagulation	-10.6
	MCODE_2	R-HSA-1280215	Cytokine Signaling in Immune system	-11.7
	MCODE_2	R-HSA-114608	Platelet degranulation	-10.0
	MCODE_2	R-HSA-76005	Response to elevated platelet cytosolic Ca2+	-9.9
	MCODE_3	R-HSA-375276	Peptide ligand-binding receptors	-26.3
	MCODE_3	R-HSA-418594	G alpha (i) signalling events	-23.8
	MCODE_3	R-HSA-373076	Class A/1 (Rhodopsin-like receptors)	-23.6
	MCODE_4	R-HSA-6798695	Neutrophil degranulation	-16.2
	MCODE_5	GO:0007169	transmembrane receptor protein tyrosine kinase signaling pathway	-9.3
	MCODE_5	hsa04010	MAPK signaling pathway	-8.6
	MCODE_5	GO:0018108	peptidyl-tyrosine phosphorylation	-8.2
	MCODE_6	ko05218	Melanoma	-15.9
	MCODE_6	hsa05218	Melanoma	-15.4
	MCODE_6	ko04015	Rap1 signaling pathway	-13.0
	MCODE_7	hsa04350	TGF-beta signaling pathway	-11.8
	MCODE_7	GO:0030509	BMP signaling pathway	-10.7
	MCODE_7	GO:0071773	cellular response to BMP stimulus	-10.6
	MCODE_8	GO:0018108	peptidyl-tyrosine phosphorylation	-9.5
	MCODE_8	GO:0018212	peptidyl-tyrosine modification	-9.5
	MCODE_8	GO:0033674	positive regulation of kinase activity	-9.1
	MCODE_9	GO:0042730	fibrinolysis	-12.4
	MCODE_9	GO:0030195	negative regulation of blood coagulation	-11.2
	MCODE_9	GO:1900047	negative regulation of hemostasis	-11.2
	MCODE_10	R-HSA-6798695	Neutrophil degranulation	-7.2
	MCODE_11	R-HSA-140834	Extrinsic Pathway of Fibrin Clot Formation	-15.8
	MCODE_11	R-HSA-140877	Formation of Fibrin Clot (Clotting Cascade)	-11.6
	MCODE_11	CORUM:3162	TF-FVIIa-FXa-TFPI complex	-11.5
	MCODE_12	R-HSA-6798695	Neutrophil degranulation	-5.4
	MCODE_13	GO:0070120	ciliary neurotrophic factor-mediated signaling pathway	-11.7
	MCODE_13	R-HSA-6783589	Interleukin-6 family signaling	-9.4
	MCODE_13	WP4538	Regulatory circuits of the STAT3 signaling pathway	-7.8
	MCODE_14	GO:2000659	regulation of interleukin-1-mediated signaling pathway	-10.6
	MCODE_14	GO:0070498	interleukin-1-mediated signaling pathway	-9.1
	MCODE_14	M110	PID IL1 PATHWAY	-8.9
	MCODE_15	R-HSA-977225	Amyloid fiber formation	-7.3
	MCODE_16	R-HSA-977606	Regulation of Complement cascade	-8.5
	MCODE_16	ko05150	Staphylococcus aureus infection	-8.2
	MCODE_16	R-HSA-166658	Complement cascade	-8.2
	MCODE_17	GO:0031589	cell-substrate adhesion	-5.8
	MCODE_18	R-HSA-9660821	ADORA2B mediated anti-inflammatory cytokines production	-7.1
	MCODE_18	R-HSA-418555	G alpha (s) signalling events	-7.0
	MCODE_18	R-HSA-9664433	Leishmania parasite growth and survival	-6.8

Supplementary Figure 6. Metascape analyses of Plasma samples from control subjects hour 24 vs hour 0 showing downregulated biological pathways and protein interaction networks. Top 20 clusters with their representative enriched terms (A). MCODE components identified in the gene lists of the protein-protein interaction networks (B). See supplementary figure 3 caption for explanation of “count” “%”, “Log10(P)”, and “Log10(q)" .

A

GO	Category	Description	Count	%	Log10(P)	Log10(q)
M5885	Canonical Pathways	NABA MATRISOME ASSOCIATED	17	31.48	-14.06	-9.70
GO:0006953	GO Biological Processes	acute-phase response	7	12.96	-11.50	-7.44
ko04060	KEGG Pathway	Cytokine-cytokine receptor interaction	10	18.52	-10.34	-6.45
WP2806	WikiPathways	Complement system	7	12.96	-9.29	-5.63
M3008	Canonical Pathways	NABA ECM GLYCOPROTEINS	8	14.81	-8.66	-5.15
R-HSA-1280215	Reactome Gene Sets	Cytokine Signaling in Immune system	11	20.37	-7.34	-4.02
GO:0048002	GO Biological Processes	antigen processing and presentation of peptide antigen	5	9.26	-7.05	-3.76
GO:0030335	GO Biological Processes	positive regulation of cell migration	8	14.81	-5.21	-2.35
GO:0010035	GO Biological Processes	response to inorganic substance	8	14.81	-5.11	-2.32
WP289	WikiPathways	Myometrial relaxation and contraction pathways	5	9.26	-5.03	-2.27
R-HSA-446203	Reactome Gene Sets	Asparagine N-linked glycosylation	6	11.11	-4.77	-2.13
WP4969	WikiPathways	RAS and bradykinin pathways in COVID-19	3	5.56	-4.72	-2.13
R-HSA-5669034	Reactome Gene Sets	TNFs bind their physiological receptors	3	5.56	-4.72	-2.13
GO:0001934	GO Biological Processes	positive regulation of protein phosphorylation	8	14.81	-4.37	-1.90
R-HSA-2173782	Reactome Gene Sets	Binding and Uptake of Ligands by Scavenger Receptors	3	5.56	-4.23	-1.83
hsa05143	KEGG Pathway	African trypanosomiasis	3	5.56	-4.17	-1.80
R-HSA-8848021	Reactome Gene Sets	Signaling by PTK6	3	5.56	-3.90	-1.62
GO:0031638	GO Biological Processes	zymogen activation	3	5.56	-3.74	-1.48
R-HSA-6798695	Reactome Gene Sets	Neutrophil degranulation	6	11.11	-3.68	-1.43
ko04918	KEGG Pathway	Thyroid hormone synthesis	3	5.56	-3.50	-1.30

B

Color	MCODE	GO	Description	Log10(P)
	MCODE_1	R-HSA-8957275	Post-translational protein phosphorylation	-9.8
	MCODE_1	R-HSA-381426	Regulation of Insulin-like Growth Factor (IGF) transport and uptake by Insulin-like Growth Factor Binding Proteins (IGFBPs)	-9.6

Supplementary Figure 7. Metascape analyses of Plasma samples from sleep deprived subjects hour 24 vs hour 0 showing downregulated biological pathways and protein interaction networks. Top 20 clusters with their representative enriched terms (A). MCODE components identified in the gene lists of the protein-protein interaction networks (B). See supplementary figure 3 caption for explanation of "count" "%", "Log10(P)", and "Log10(q)".

	Control T24 vs T0			SlpD T24 vs T0		
**Protein**	**Fold Change**	**p-value**	**adj. p-value**	**Fold Change**	**p-value**	**adj. p-value**
Hemoglobin	5.445114	**0.004877**	0.098123	1.446468	0.181927	0.709305
Carbonic anhydrase 3	2.427809	**0.013614**	0.099154	1.350113	0.165627	0.709305
NSFL1 cofactor p47	2.309426	**0.010738**	0.098211	1.362604	0.11378	0.709305
Ubiquitin	2.25569	**0.003656**	0.098123	-1.976762	**0.021223**	0.709305
Protein 4.1	2.238844	**0.01216**	0.098211	1.434799	0.129798	0.709305
Malate dehydrogenase, cytoplasmic	2.205248	**0.012347**	0.098211	1.342183	0.067137	0.709305
Rab GDP dissociation inhibitor beta	2.19454	**0.023876**	0.108271	1.362748	0.123128	0.709305
Serine/threonine-protein kinase WNK3	2.0887	**0.021077**	0.107437	1.368448	0.196629	0.709305
Aflatoxin B1 aldehyde reductase member 2	2.063057	**0.04164**	0.129073	1.24228	0.472793	0.796123
Fibroblast growth factor 8 isoform A	1.988496	**0.028502**	0.114408	1.260311	0.233698	0.709305
Heterogeneous nuclear ribonucleoprotein A/B	1.965525	**0.022776**	0.108271	1.262478	0.297451	0.713675
SUMO-conjugating enzyme UBC9	1.952166	**0.037528**	0.1256	1.290152	0.252124	0.709305
Interferon lambda-1	1.932702	**0.026794**	0.112071	1.233411	0.101151	0.709305
Desert hedgehog protein N-product	1.913464	**0.02758**	0.113137	1.258356	0.136943	0.709305
CD40 ligand	1.899174	**0.016128**	0.101247	1.178389	0.118898	0.709305
Proteasome subunit alpha type-6	1.883612	**0.04335**	0.130649	1.266797	0.164953	0.709305
DnaJ homolog subfamily B member 1	1.880522	**0.042602**	0.13	1.387991	0.079771	0.709305
Proteasome activator complex subunit 1	1.862498	**0.039688**	0.127353	1.17854	0.243535	0.709305
Fibroblast growth factor 6	1.855325	**0.027077**	0.112489	1.188767	0.156597	0.709305
Alcohol dehydrogenase [NADP(+)]	1.839176	**0.014726**	0.099154	1.288616	**0.04753**	0.709305
Ubiquitin-like protein ISG15	1.830138	**0.028668**	0.114408	1.194721	0.127738	0.709305
AT-rich interactive domain-containing protein 3A	1.829495	**0.035673**	0.122509	1.250914	0.140076	0.709305
Small ubiquitin-related modifier 3	1.812233	**0.04731**	0.137505	1.233458	0.178046	0.709305
Ubiquitin-fold modifier 1	1.81041	**0.022682**	0.108271	1.321884	0.188356	0.709305
Kremen protein 2	1.79908	**0.016864**	0.101247	1.168322	0.130803	0.709305
S-formylglutathione hydrolase	1.797249	**0.022839**	0.108271	1.237084	0.173098	0.709305
Dual specificity mitogen-activated protein kinase kinase 3	1.79604	**0.039651**	0.127353	1.174606	0.176571	0.709305
Apoptosis regulator Bcl-2	1.787859	**0.045651**	0.134479	1.254415	0.113108	0.709305
Glucocorticoid receptor	1.785956	**0.011334**	0.098211	1.030864	0.815447	0.933932
Eukaryotic initiation factor 4A-III	1.781463	**0.005838**	0.098123	1.088673	0.307732	0.713675
Angiotensin-converting enzyme 2	1.774739	**0.013266**	0.099136	1.126636	0.183145	0.709305
Ribosomal protein S6 kinase alpha-3	1.756809	**0.017197**	0.101247	1.268653	0.193805	0.709305
Vasoactive Intestinal Peptide	1.754076	**0.032559**	0.120297	1.214302	0.081634	0.709305
Casein kinase II 2-alpha':2-beta heterotetramer	1.751835	**0.013074**	0.098619	1.187842	0.145286	0.709305
Calcium/calmodulin-dependent protein kinase kinase 1	1.709649	**0.015994**	0.101247	1.057423	0.537682	0.83399
cGMP-dependent 3',5'-cyclic phosphodiesterase	1.699727	**0.006433**	0.098123	1.011299	0.863424	0.948903
Leukocyte surface antigen CD47	1.698734	**0.025914**	0.11038	1.180791	0.071812	0.709305
Fibroblast growth factor 7	1.694894	**0.024128**	0.108271	1.10653	0.325871	0.713675
Alpha-enolase	1.693679	**0.04842**	0.139489	1.180834	0.414721	0.767268
Peptidyl-prolyl cis-trans isomerase A	1.690459	**0.045233**	0.13355	1.285175	0.235877	0.709305
Heat shock protein HSP 90-alpha/beta	1.687027	**0.011424**	0.098211	1.180583	0.256205	0.709305
Peptidyl-prolyl cis-trans isomerase E	1.665334	**0.04773**	0.13811	1.204564	0.228276	0.709305
Arginase-1	1.661083	**0.007795**	0.098123	1.093947	0.245262	0.709305
Adapter molecule crk	1.66079	**0.008245**	0.098123	1.276064	**0.049399**	0.709305
Mesothelin	1.65571	**0.007389**	0.098123	1.129831	0.104171	0.709305
Heat shock protein beta-1	1.642786	**0.033655**	0.120328	1.207016	0.3926	0.759027
NKG2D ligand 3	1.64193	**0.029548**	0.116142	1.154202	0.142931	0.709305
14-3-3 protein epsilon	1.634393	**0.015458**	0.100862	1.03332	0.831699	0.941467
Receptor-type tyrosine-protein kinase FLT3	1.632408	**0.012542**	0.098211	1.166038	0.146424	0.709305
Mitogen-activated protein kinase 1	1.622529	**0.036474**	0.123749	1.171222	0.412202	0.767268
Tyrosine-protein phosphatase non-receptor type 11	1.622304	**0.033534**	0.120328	1.204084	0.194082	0.709305
Azurocidin	1.617306	**0.024226**	0.108271	1.119336	0.277944	0.711716
Vascular endothelial growth factor C	1.614752	**0.028633**	0.114408	1.139361	0.1062	0.709305
Pyridoxal kinase	1.611492	**0.003232**	0.098123	1.190557	**0.045891**	0.709305
Galectin-4	1.608304	**0.009265**	0.098211	1.112232	0.085653	0.709305
Histone acetyltransferase type B catalytic subunit	1.594279	**0.0334**	0.120328	1.117552	0.277402	0.711716
Methyl-CpG-binding domain protein 4	1.591293	**0.011372**	0.098211	1.107297	0.192016	0.709305
Glucose-6-phosphate isomerase	1.589937	**0.007889**	0.098123	1.128915	0.412087	0.767268
Leucine-rich repeat transmembrane protein FLRT1	1.588822	**0.04297**	0.130105	1.125978	0.18917	0.709305
Heat shock protein HSP 90-beta	1.588802	**0.010236**	0.098211	1.165514	0.232524	0.709305
Keratin, type I cytoskeletal 18	1.584276	**0.013559**	0.099154	1.118438	0.101478	0.709305
Ribosomal protein S6 kinase alpha-5	1.578419	**0.040941**	0.127697	1.107205	0.325003	0.713675
N-acetyl-D-glucosamine kinase	1.57685	**0.005909**	0.098123	1.161893	0.120747	0.709305
Calpain I	1.57311	**0.013915**	0.099154	1.232713	0.193779	0.709305
Macrophage migration inhibitory factor	1.56151	**0.025557**	0.11038	1.034912	0.602401	0.849393
L-lactate dehydrogenase B chain	1.558891	**0.008107**	0.098123	1.135386	**0.017295**	0.709305
Cytotoxic and regulatory T-cell molecule	1.557695	**0.022404**	0.108271	1.111079	0.14696	0.709305
Cyclin-dependent kinase 1:G2/mitotic-specific cyclin-B1 complex	1.554883	**0.014415**	0.099154	1.10342	0.089612	0.709305
Erythropoietin receptor	1.551583	**0.017798**	0.10228	1.088009	0.10598	0.709305
Kynureninase	1.539486	**0.003282**	0.098123	1.119747	**0.015803**	0.709305
Calcium/calmodulin-dependent 3',5'-cyclic nucleotide phosphodiesterase 1A	1.531621	**0.040799**	0.127697	1.134032	0.146024	0.709305
Fructose-bisphosphate aldolase A	1.530237	**0.007927**	0.098123	1.205241	0.093775	0.709305
Tumor-associated calcium signal transducer 2	1.52577	**0.012483**	0.098211	1.056701	0.229699	0.709305
Chloride intracellular channel protein 1	1.511716	**0.032268**	0.120297	1.187251	0.431423	0.777634
40S ribosomal protein S3a	1.510099	**0.045046**	0.1333	1.104266	0.525826	0.828815
Growth/differentiation factor 11/8	1.509886	**0.00015**	0.096991	1.140399	**0.024818**	0.709305
Xaa-Pro aminopeptidase 1	1.501625	**0.023259**	0.108271	1.19804	0.194341	0.709305
Thymic stromal lymphopoietin	1.493445	**0.021693**	0.108197	1.121224	0.099945	0.709305
DNA topoisomerase 1	1.490343	**0.044965**	0.1333	1.204414	0.479402	0.802076
Proteasome subunit alpha type-1	1.468599	**0.027325**	0.112489	1.055977	0.278709	0.711716
40S ribosomal protein S7	1.467145	**0.042439**	0.13	1.213432	0.44721	0.783794
Fibroblast growth factor 4	1.45799	**0.006399**	0.098123	1.088983	0.17731	0.709305
CD63 antigen	1.45092	**0.009393**	0.098211	1.030653	0.593038	0.847662
Tumor necrosis factor receptor superfamily member EDAR	1.445394	**0.037907**	0.1256	1.15784	0.189809	0.709305
Gamma-enolase	1.435646	**0.002662**	0.098123	1.184048	0.060773	0.709305
Serine/threonine-protein kinase TBK1	1.430757	**0.018624**	0.102987	1.035702	0.45189	0.787243
Follistatin	1.430263	**0.038398**	0.126197	1.080883	0.306564	0.713675
Signal transducer and activator of transcription 6	1.424949	**0.005617**	0.098123	1.078339	0.446549	0.783794
Cytokine receptor-like factor 2	1.420702	**0.011945**	0.098211	1.077381	0.151285	0.709305
Heparin-binding EGF-like growth factor	1.404475	**0.020016**	0.106459	1.084017	0.151879	0.709305
Interleukin-23 receptor	1.401729	**0.028904**	0.114651	1.058793	0.266004	0.711716
Interleukin-11	1.398085	**0.021902**	0.108264	1.022988	0.668773	0.880675
Interferon regulatory factor 1	1.396218	**0.007687**	0.098123	-1.03996	0.257254	0.709305
Platelet receptor Gi24	1.396026	**0.0332**	0.120328	1.005785	0.92775	0.970123
Lamin-B1	1.388613	**0.022194**	0.108271	1.045211	0.345498	0.722487
Serine/threonine-protein kinase receptor R3	1.3881	**0.018537**	0.102987	1.08071	0.080316	0.709305
Testican-1	1.387588	**0.020952**	0.107373	1.018487	0.773966	0.923142
Laminin	1.379889	**0.000969**	0.096991	1.05295	0.480975	0.80265
Chymase	1.377915	**0.037221**	0.125512	1.101656	0.624343	0.853279
alpha-Fetoprotein	1.376247	**0.00695**	0.098123	1.034261	0.346019	0.722487
Interleukin-4	1.371423	**0.012507**	0.098211	1.035756	0.400443	0.761746
Corticosteroid-binding globulin	1.364701	**0.030712**	0.117189	-1.025324	0.589852	0.847423
Ubiquitin-fold modifier-conjugating enzyme 1	1.346836	**0.028246**	0.114408	1.14491	0.220709	0.709305
NudC domain-containing protein 3	1.343086	**0.008021**	0.098123	1.120553	0.077001	0.709305
Pescadillo homolog	1.342624	**0.014214**	0.099154	1.071323	0.313654	0.713675
Ectonucleoside triphosphate diphosphohydrolase 3	1.338567	**0.008755**	0.098211	1.050048	0.203434	0.709305
Alanine aminotransferase 1	1.335927	**0.042947**	0.130105	1.018391	0.742776	0.912481
BH3-interacting domain death agonist	1.319148	**0.020981**	0.107373	1.026374	0.610071	0.849515
Calcineurin subunit B type 1	1.319036	**0.009962**	0.098211	1.022482	0.691904	0.892228
PIK3CA/PIK3R1	1.318937	**0.003381**	0.098123	1.105282	0.205036	0.709305
Calcium-dependent phospholipase A2	1.311907	**0.038271**	0.12612	1.063088	0.158247	0.709305
Protein lin-7 homolog B	1.310202	**0.011036**	0.098211	1.113163	**0.01326**	0.709305
40S ribosomal protein S3	1.299488	**0.007427**	0.098123	1.107948	0.56681	0.838021
Tumor necrosis factor receptor superfamily member 13B	1.298979	**0.038233**	0.12612	1.109031	0.081388	0.709305
Cathepsin D	1.291786	0.395739	0.498975	-1.885102	**0.001245**	0.378768
Inducible T-cell costimulator	1.283242	**0.02138**	0.107726	1.050746	0.5807	0.839227
C-C motif chemokine 20	1.282237	**0.014773**	0.099154	1.040579	0.667268	0.87958
Angiopoietin-4	1.281278	**0.007607**	0.098123	1.036931	0.248274	0.709305
Estrogen receptor	1.27902	**0.03472**	0.121343	-1.014135	0.770242	0.923018
B-cell receptor CD22	1.270031	**0.017894**	0.10228	1.054967	0.155582	0.709305
Tyrosine-protein kinase Yes	1.268462	**0.042668**	0.13	1.091456	0.091549	0.709305
Protein NOV homolog	1.263395	**0.014882**	0.099154	1.054344	0.137191	0.709305
C-C motif chemokine 27	1.260437	**0.000385**	0.096991	1.026088	0.615487	0.849515
Sialoadhesin	1.255226	**0.000817**	0.096991	1.068269	0.203654	0.709305
Epidermal growth factor receptor substrate 15-like 1	1.254124	**0.026148**	0.11038	1.059646	0.269502	0.711716

	Control T24 vs T0			SlpD T24 vs T0		
**Protein**	**Fold Change**	**p-value**	**adj. p-value**	**Fold Change**	**p-value**	**adj. p-value**
Group 10 secretory phospholipase A2	1.250843	**0.003284**	0.098123	1.022779	0.613331	0.849515
Killer cell immunoglobulin-like receptor 3DL2	1.24762	**0.00709**	0.098123	1.007028	0.844614	0.944592
60 kDa heat shock protein, mitochondrial	1.245719	**0.023217**	0.108271	1.177717	0.220756	0.709305
Cytoplasmic tyrosine-protein kinase BMX	1.244301	**0.025748**	0.11038	1.025747	0.391169	0.758906
Connective tissue growth factor	1.243122	**0.049766**	0.141173	-1.011479	0.860278	0.948903
3-hydroxyacyl-CoA dehydrogenase type-2	1.239892	**0.038956**	0.127095	1.127668	0.25449	0.709305
Ephrin type-A receptor 5	1.232171	**0.005435**	0.098123	1.035944	0.515397	0.825225
Killer cell immunoglobulin-like receptor 3DS1	1.224001	**0.012948**	0.098619	-1.021299	0.494467	0.810354
alpha-2-macroglobulin receptor-associated protein	1.223265	**0.007712**	0.098123	-1.034197	0.440867	0.783794
Aromatic-L-amino-acid decarboxylase	1.219647	**0.001467**	0.098123	1.072054	0.07816	0.709305
High affinity cAMP-specific 3',5'-cyclic phosphodiesterase 7A	1.20693	**0.014598**	0.099154	1.014705	0.642841	0.863074
Dickkopf-like protein 1	1.204866	**0.032662**	0.120297	-1.038474	0.539105	0.83399
Casein kinase II subunit alpha	1.20241	**0.020368**	0.106459	1.03242	0.553015	0.835896
Brevican core protein	1.192687	**0.035312**	0.122139	1.334924	**0.000706**	0.307242
Glia-derived nexin	1.192339	**0.029665**	0.116142	1.096331	0.513357	0.825225
Cysteine-rich secretory protein 3	1.187379	**0.007592**	0.098123	1.054943	0.619982	0.849871
Neurexophilin-1	1.186339	**0.019814**	0.106407	-1.030501	0.616261	0.849515
Interleukin-17 receptor A	1.17944	**0.016586**	0.101247	-1.026866	0.596082	0.848296
Drebrin-like protein	1.178578	**0.009106**	0.098211	-1.011208	0.913045	0.964952
Tyrosine-protein kinase Lck	1.175486	**0.01134**	0.098211	1.017085	0.580706	0.839227
Serum paraoxonase/arylesterase 1	1.173723	**0.021775**	0.108197	1.028175	0.459249	0.792352
Interleukin-20 receptor subunit alpha	1.172798	**0.000756**	0.096991	1.045525	0.279261	0.711716
Kin of IRRE-like protein 3	1.167868	**0.009722**	0.098211	1.007132	0.848067	0.945444
Dual specificity mitogen-activated protein kinase kinase 1	1.165512	**0.00567**	0.098123	1.040727	0.390601	0.758906
Transforming growth factor beta-2	1.16449	**0.024511**	0.10843	-1.000424	0.992875	1
14-3-3 protein theta	1.160009	**0.049974**	0.141173	1.024469	0.678526	0.886315
Killer cell lectin-like receptor subfamily F member 1	1.15543	**0.014191**	0.099154	-1.02694	0.458566	0.792352
A disintegrin and metalloproteinase with thrombospondin motifs 5	1.1549	**0.039267**	0.127353	-1.054338	0.343828	0.722487
Ectonucleoside triphosphate diphosphohydrolase 1	1.152528	**0.014594**	0.099154	1.007053	0.902174	0.964952
Contactin-1	1.152331	**0.027742**	0.113137	-1.020952	0.576324	0.838021
Bcl-2-like protein 1	1.147957	**0.003446**	0.098123	1.066876	0.129778	0.709305
Kallikrein-4	1.146939	**0.011727**	0.098211	1.013072	0.662649	0.877926
26S proteasome non-ATPase regulatory subunit 7	1.138285	**0.007971**	0.098123	-1.022077	0.519966	0.827854
Natural cytotoxicity triggering receptor 3	1.135176	**0.006874**	0.098123	-1.00572	0.899588	0.964952
Kelch-like ECH-associated protein 1	1.135014	**0.003508**	0.098123	-1.029987	0.492726	0.810354
Epidermal growth factor receptor variant III	1.134418	**0.023924**	0.108271	-1.032586	0.337772	0.716341
Interleukin-13	1.129441	**0.014168**	0.099154	1.000379	0.992122	1
Desmoglein-1	1.124276	**0.039917**	0.127496	-1.002962	0.95508	0.986024
Heme oxygenase 2	1.118785	**0.018521**	0.102987	1.008251	0.860124	0.948903
Interleukin-16	1.11774	**0.034947**	0.121615	1.069574	0.527884	0.828815
Protein disulfide-isomerase	1.113296	**0.008539**	0.098123	1.098076	**0.031522**	0.709305
Cytosolic non-specific dipeptidase	1.112109	**0.023108**	0.108271	1.033125	0.402178	0.761746
Plasmin	1.108198	**0.045872**	0.134524	-1.012828	0.750356	0.914577
Proliferating cell nuclear antigen	1.095857	**0.030501**	0.116726	1.005543	0.88168	0.955641
Ciliary neurotrophic factor	1.095547	**0.024347**	0.108275	1.002499	0.953886	0.986024
Protein-tyrosine kinase 6	1.095265	**0.041**	0.127697	-1.006498	0.914666	0.965727
Granzyme B	1.093744	**0.027283**	0.112489	1.028828	0.637191	0.860802
E3 ubiquitin-protein ligase Mdm2	1.090169	**0.01337**	0.099136	-1.003128	0.927337	0.970123
X-linked interleukin-1 receptor accessory protein-like 2	1.079633	**0.026057**	0.11038	-1.027326	0.612523	0.849515
Ephrin type-A receptor 10	1.076598	0.187679	0.296156	1.182942	**0.005557**	0.628677
Interferon alpha-2	1.066715	**0.039959**	0.127496	1.09267	0.061086	0.709305
Neurexin-1-beta	1.03231	0.740486	0.797208	1.205406	**0.016833**	0.709305
Netrin receptor UNC5D	1.022354	0.894137	0.919503	1.96852	**0.000591**	0.307242
Glucokinase regulatory protein	1.018208	0.784035	0.83592	-1.088119	**0.049642**	0.709305
Interleukin-8	1.002269	0.976092	0.982597	1.209328	**0.005106**	0.628677
Lymphocyte antigen 86	-1.009336	0.829517	0.869493	-1.09354	**0.044281**	0.709305
C-X-C motif chemokine 6	-1.020187	0.885819	0.914552	1.357367	**0.038435**	0.709305
Alpha-1-antitrypsin	-1.042262	0.638072	0.712914	-1.245972	**0.028506**	0.709305
Interleukin-6	-1.045055	0.782823	0.83531	-1.30285	**0.020149**	0.709305
Tumor necrosis factor receptor superfamily member 6B	-1.049242	0.275627	0.385523	-1.09932	**0.046086**	0.709305
Dipeptidyl peptidase 2	-1.061713	0.265011	0.377554	-1.135392	**0.019753**	0.709305
Leucine-rich repeat transmembrane neuronal protein 3	-1.081707	**0.046999**	0.136904	-1.046496	0.292691	0.713675
Tumor necrosis factor receptor superfamily member 13C	-1.085098	**0.017779**	0.10228	-1.066735	0.141058	0.709305
Apolipoprotein E (isoform E4)	-1.089878	**0.023742**	0.108271	-1.002124	0.949976	0.983123
Calreticulin	-1.089951	0.09471	0.190735	-1.114401	**0.036908**	0.709305
Bone morphogenetic protein receptor type-1A	-1.090775	0.540125	0.629342	1.400299	**0.005299**	0.628677
Insulin-like growth factor-binding protein 4	-1.095404	0.269533	0.380792	-1.160814	**0.025524**	0.709305
Protein kinase C gamma type	-1.096709	0.205297	0.315392	-1.174059	**0.049783**	0.709305
Tumor necrosis factor receptor superfamily member 10B	-1.098661	**0.024909**	0.109449	-1.056944	0.275693	0.711716
Prolactin receptor	-1.099781	**0.018105**	0.10228	-1.062322	0.21405	0.709305
Coagulation factor VII	-1.101977	**0.041313**	0.128366	-1.08201	0.094171	0.709305
Chorionic somatomammotropin hormone	-1.109647	**0.032416**	0.120297	-1.079773	0.249104	0.709305
ADP-ribosyl cyclase/cyclic ADP-ribose hydrolase 1	-1.110165	**0.028193**	0.114408	-1.066247	0.220238	0.709305
Betacellulin	-1.110466	0.07365	0.164961	-1.109246	**0.025191**	0.709305
Cytotoxic T-lymphocyte protein 4	-1.114869	**0.040445**	0.127697	1.00869	0.917434	0.967085
GRB2-related adapter protein 2	-1.116328	**0.03147**	0.118846	-1.078437	0.200263	0.709305
Transcription factor AP-1	-1.116562	**0.037491**	0.1256	-1.069427	0.291206	0.713675
CCAAT/enhancer-binding protein beta	-1.118549	**0.037653**	0.1256	-1.053099	0.441911	0.783794
Dual specificity tyrosine-phosphorylation-regulated kinase 3	-1.118749	**0.016814**	0.101247	-1.100676	0.07375	0.709305
Interleukin-1 receptor antagonist protein	-1.119361	**0.049329**	0.141173	-1.058005	0.320511	0.713675
NADPH--cytochrome P450 reductase	-1.119705	0.288996	0.399936	1.24282	**0.004839**	0.628677
Placenta growth factor	-1.121248	**0.046676**	0.136269	-1.054268	0.32831	0.713675
Tartrate-resistant acid phosphatase type 5	-1.121916	**0.034525**	0.121343	-1.105568	0.154703	0.709305
Tumor necrosis factor	-1.122273	**0.033581**	0.120328	-1.10011	0.114895	0.709305
Insulin-like growth factor-binding protein 6	-1.124511	0.057514	0.148625	-1.112587	**0.042172**	0.709305
Legumain	-1.127391	0.070226	0.161917	-1.161088	**0.04411**	0.709305
Alpha-2-antiplasmin	-1.128505	**0.040835**	0.127697	-1.062762	0.291234	0.713675
Interleukin-1 alpha	-1.130468	**0.045764**	0.134508	-1.093406	**0.04002**	0.709305
Fibroblast growth factor 18	-1.13715	**0.029725**	0.116142	-1.089677	0.114198	0.709305
Transmembrane glycoprotein NMB	-1.137551	0.15621	0.260349	-1.166247	**0.032335**	0.709305
Chitotriosidase-1	-1.138966	**0.039719**	0.127353	-1.030723	0.678116	0.886315
Insulin-like growth factor-binding protein 3	-1.139655	**0.049787**	0.141173	-1.104582	0.157545	0.709305
E3 ubiquitin-protein ligase RNF43	-1.141231	**0.044515**	0.132629	-1.081084	0.219544	0.709305
Arylsulfatase A	-1.144319	**0.040644**	0.127697	-1.050428	0.42786	0.776854
3-hydroxy-3-methylglutaryl-coenzyme A reductase	-1.145046	**0.005575**	0.098123	-1.090424	0.054685	0.709305
C-X-C motif chemokine 9	-1.145191	**0.040649**	0.127697	-1.052866	0.386298	0.755866
Vitamin K-dependent protein S	-1.146303	**0.046205**	0.135196	-1.080866	0.158804	0.709305
Growth arrest-specific protein 1	-1.147376	**0.025423**	0.11038	-1.116629	0.069863	0.709305
3-hydroxyanthranilate 3,4-dioxygenase	-1.147501	**0.037921**	0.1256	-1.142546	**0.012616**	0.709305
Complement C5	-1.148671	**0.022344**	0.108271	-1.089138	0.066528	0.709305
Layilin	-1.148777	**0.025025**	0.109589	-1.048809	0.474922	0.79765
Plasminogen	-1.149981	**0.02993**	0.116592	-1.059408	0.251784	0.709305
Macrophage colony-stimulating factor 1 receptor	-1.15004	**0.016425**	0.101247	-1.133322	**0.042099**	0.709305
cAMP-specific 3',5'-cyclic phosphodiesterase 4D	-1.150225	**0.017515**	0.101586	-1.046334	0.369244	0.740047
Netrin-1	-1.151959	**0.044435**	0.132629	-1.079815	0.233535	0.709305
Testican-2	-1.152293	**0.027673**	0.113137	-1.07561	0.236365	0.709305
P-selectin	-1.155624	**0.037712**	0.1256	1.007936	0.84746	0.945444
Ferritin	-1.157318	**0.043296**	0.130649	-1.126088	0.068003	0.709305
Platelet-derived growth factor C	-1.158366	**0.049436**	0.141173	-1.07485	0.2924	0.713675
Phosphoglucomutase-1	-1.160938	**0.026268**	0.11038	-1.080315	0.184705	0.709305
Vascular endothelial growth factor A, isoform 121	-1.163025	**0.034776**	0.121343	-1.015102	0.796322	0.931039
Tumor necrosis factor ligand superfamily member 9	-1.163351	**0.028579**	0.114408	-1.042884	0.482884	0.802756
Sex hormone-binding globulin	-1.164615	**0.033013**	0.120328	-1.045893	0.413642	0.767268
R-spondin-4	-1.164851	**0.012794**	0.098211	-1.025655	0.563787	0.837279
Low affinity immunoglobulin gamma Fc region receptor II-a	-1.165661	**0.023693**	0.108271	-1.052621	0.42481	0.774937
Tumor necrosis factor receptor superfamily member 10A	-1.166169	**0.034053**	0.120538	-1.151172	**0.034711**	0.709305
Discoidin domain-containing receptor 2	-1.167379	**0.005519**	0.098123	-1.112306	0.061957	0.709305
Angiogenin	-1.169129	**0.031465**	0.118846	-1.0314	0.626266	0.853999
Interleukin-5	-1.169495	**0.013883**	0.099154	-1.054545	0.397626	0.760394
Cadherin-6	-1.169711	**0.026305**	0.11038	-1.181654	**0.016588**	0.709305
Mitogen-activated protein kinase 11	-1.170538	**0.01859**	0.102987	-1.072403	0.308594	0.713675
Fatty acid-binding protein, epidermal	-1.171394	**0.019655**	0.105991	-1.071849	0.222862	0.709305
Hepatitis A virus cellular receptor 2	-1.171788	**0.036003**	0.123286	-1.02955	0.714254	0.900581
Cystatin-F	-1.171852	**0.029599**	0.116142	1.126906	**0.03421**	0.709305
Complement factor I	-1.172	**0.036508**	0.123749	1.014714	0.689932	0.891951

	Control T24 vs T0			SlpD T24 vs T0		
**Protein**	**Fold Change**	**p-value**	**adj. p-value**	**Fold Change**	**p-value**	**adj. p-value**
Angiotensinogen	-1.172243	**0.034083**	0.120538	-1.039609	0.554016	0.835896
Heparin cofactor 2	-1.172495	**0.015889**	0.101247	-1.070043	0.206191	0.709305
Down syndrome cell adhesion molecule	-1.173675	**0.025557**	0.11038	-1.070336	0.219163	0.709305
Lymphotoxin alpha2:beta1	-1.176575	**0.012629**	0.098211	1.001309	0.987875	1
Synaptosomal-associated protein 25	-1.17663	**0.015911**	0.101247	-1.093483	0.268144	0.711716
Tumor necrosis factor receptor superfamily member 4	-1.176902	**0.011658**	0.098211	-1.122582	**0.045207**	0.709305
Abelson tyrosine-protein kinase 2	-1.178703	**0.044435**	0.132629	-1.040841	0.569556	0.838021
Thyroxine-binding globulin	-1.179539	**0.049316**	0.141173	-1.013101	0.74327	0.912481
Beta-2-microglobulin	-1.182882	**0.004047**	0.098123	-1.106143	0.051874	0.709305
Fibroblast growth factor 23	-1.183216	**0.045009**	0.1333	-1.012002	0.878827	0.955341
D-dimer	-1.183737	**0.010356**	0.098211	-1.110256	0.053862	0.709305
Hepatocyte growth factor activator	-1.183854	**0.007163**	0.098123	-1.093028	0.098568	0.709305
Tumor necrosis factor receptor superfamily member 17	-1.184622	**0.030312**	0.116726	-1.057163	0.353155	0.724253
Thioredoxin domain-containing protein 12	-1.186346	**0.018536**	0.102987	-1.113708	0.117738	0.709305
Carboxypeptidase B2	-1.186981	**0.008585**	0.098123	-1.073557	0.144803	0.709305
Fetuin-B	-1.187561	**0.010696**	0.098211	1.008115	0.867415	0.948903
Thyroid Stimulating Hormone	-1.187705	0.184881	0.293112	-1.524207	**0.005781**	0.628677
Cystatin-C	-1.188794	**0.008326**	0.098123	-1.026995	0.670599	0.881614
Hepatocyte growth factor-like protein	-1.189129	**0.005406**	0.098123	-1.104147	0.109818	0.709305
Programmed cell death 1 ligand 1	-1.189735	**0.042736**	0.13	-1.088779	0.239798	0.709305
Reticulon-4 receptor	-1.190907	**0.023353**	0.108271	-1.087837	0.224603	0.709305
Interleukin-1 receptor type 2	-1.1923	**0.033566**	0.120328	-1.095666	0.147121	0.709305
Mast/stem cell growth factor receptor Kit	-1.193047	**0.042649**	0.13	-1.14905	0.055636	0.709305
Cadherin-5	-1.193452	**0.035378**	0.122139	-1.098836	0.159726	0.709305
Fibrinogen gamma chain	-1.193801	**0.016591**	0.101247	-1.019544	0.634278	0.858644
Bcl-2-like protein 2	-1.194391	**0.039278**	0.127353	-1.087368	0.170814	0.709305
beta-nerve growth factor	-1.195741	**0.030053**	0.116722	-1.088478	0.229255	0.709305
Immunoglobulin M	-1.196975	**0.009663**	0.098211	-1.093537	0.096009	0.709305
Immunoglobulin A	-1.197261	**0.015976**	0.101247	-1.097079	0.077118	0.709305
Complement factor H	-1.197502	**0.012789**	0.098211	-1.030073	0.257983	0.709305
Vascular endothelial growth factor receptor 2	-1.197957	**0.014968**	0.099154	-1.070918	0.268407	0.711716
Vascular endothelial growth factor A	-1.198082	**0.01532**	0.100468	-1.051005	0.536585	0.83399
CD5 antigen-like	-1.199184	**0.003343**	0.098123	-1.121295	**0.031644**	0.709305
Brain-specific serine protease 4	-1.199296	**0.032724**	0.120297	1.02294	0.778281	0.923179
Repulsive guidance molecule A	-1.199464	**0.02388**	0.108271	-1.089454	0.189127	0.709305
Tyrosine-protein kinase receptor Tie-1, soluble	-1.19999	**0.040208**	0.127697	-1.093565	0.194826	0.709305
Vascular cell adhesion protein 1	-1.200844	**0.02244**	0.108271	-1.062069	0.261038	0.711177
SPARC-related modular calcium-binding protein 1	-1.201014	**0.023558**	0.108271	-1.144246	**0.042626**	0.709305
Coagulation factor IXab	-1.201662	**0.010695**	0.098211	-1.083961	0.147208	0.709305
Endoplasmic reticulum resident protein 29	-1.201692	**0.023162**	0.108271	-1.06298	0.312022	0.713675
Lysosomal protective protein	-1.201835	0.12033	0.215411	-1.192035	**0.034769**	0.709305
Interleukin-6 receptor subunit alpha	-1.202052	**0.036252**	0.123523	-1.085599	0.167026	0.709305
Neuregulin-4	-1.202474	**0.015203**	0.1002	-1.092635	0.250974	0.709305
Glypican-3	-1.203119	**0.021597**	0.108197	-1.029578	0.702592	0.897146
Fibroblast growth factor 5	-1.204005	**0.008435**	0.098123	-1.09223	0.093827	0.709305
Heparan-sulfate 6-O-sulfotransferase 1	-1.204736	**0.026915**	0.112218	-1.069151	0.342155	0.72018
Contactin-2	-1.205807	**0.042112**	0.13	-1.028678	0.642574	0.863074
Interleukin-17 receptor D	-1.205848	**0.014241**	0.099154	-1.070526	0.276711	0.711716
Trypsin-3	-1.206847	**0.017483**	0.101586	-1.08973	0.209138	0.709305
Collectin-12	-1.207047	**0.040601**	0.127697	-1.050515	0.560682	0.837279
C5a anaphylatoxin	-1.207216	**0.017124**	0.101247	-1.057446	0.394434	0.760394
Ectodysplasin-A, secreted form	-1.208924	**0.036088**	0.123286	-1.045096	0.529775	0.828815
Cytoskeleton-associated protein 2	-1.209114	**0.011179**	0.098211	-1.080943	0.276742	0.711716
Cathepsin F	-1.209338	**0.049653**	0.141173	-1.219715	**0.027679**	0.709305
OX-2 membrane glycoprotein	-1.210458	**0.038488**	0.126197	-1.038181	0.64172	0.863074
Endothelial cell-selective adhesion molecule	-1.210926	**0.017293**	0.101247	-1.084632	0.271065	0.711716
Cathepsin Z	-1.211578	**0.042233**	0.13	-1.104234	0.24077	0.709305
Serum albumin	-1.212158	**0.02521**	0.11003	-1.110193	**0.049444**	0.709305
CD166 antigen	-1.214079	**0.025667**	0.11038	-1.098736	0.112542	0.709305
Angiostatin	-1.214726	**0.02124**	0.107437	-1.099763	0.172125	0.709305
Lysozyme C	-1.214752	**0.043679**	0.131036	-1.020279	0.72587	0.903611
R-spondin-3	-1.21496	**0.033242**	0.120328	-1.078127	0.230368	0.709305
Tumor necrosis factor receptor superfamily member 19	-1.215639	**0.02214**	0.108271	-1.099305	0.222196	0.709305
Antithrombin-III	-1.21578	**0.014897**	0.099154	1.011706	0.726351	0.903611
C-C motif chemokine 19	-1.215901	**0.048097**	0.138865	-1.066899	0.4575	0.791826
Adrenomedullin	-1.216869	**0.001849**	0.098123	-1.114041	**0.044571**	0.709305
Complement component C9	-1.217056	**0.008647**	0.098123	-1.011018	0.814826	0.933932
Small nuclear ribonucleoprotein F	-1.217616	**0.00357**	0.098123	-1.137633	**0.039497**	0.709305
Advanced glycosylation end product-specific receptor, soluble	-1.217634	**0.00608**	0.098123	-1.001146	0.984476	0.998545
Transforming growth factor beta receptor type 3	-1.217636	**0.037201**	0.125512	-1.092483	0.190396	0.709305
C-C motif chemokine 21	-1.218668	**0.02071**	0.107247	-1.10763	0.102613	0.709305
Neurogenic locus notch homolog protein 1	-1.219438	**0.019039**	0.103524	-1.136229	0.071817	0.709305
Pigment epithelium-derived factor	-1.21959	**0.030212**	0.116726	-1.062387	0.123572	0.709305
Low-density lipoprotein receptor-related protein 1B	-1.220136	**0.031636**	0.118977	-1.035636	0.313215	0.713675
Mannan-binding lectin serine protease 1	-1.221053	**0.03265**	0.120297	-1.121757	0.190395	0.709305
Toll-like receptor 4:Lymphocyte antigen 96 complex	-1.221859	**0.03151**	0.118846	-1.067326	0.381477	0.750871
Dynactin subunit 2	-1.222574	**0.003608**	0.098123	-1.180274	**0.01132**	0.709305
Cathepsin L2	-1.222649	**0.034652**	0.121343	-1.065045	0.520184	0.827854
A disintegrin and metalloproteinase with thrombospondin motifs 13	-1.224195	**0.016204**	0.101247	-1.143583	0.078468	0.709305
Coagulation Factor V	-1.224661	**0.034385**	0.121277	1.033225	0.467401	0.794636
Cadherin-3	-1.22551	**0.037793**	0.1256	-1.128266	0.180029	0.709305
Vascular endothelial growth factor receptor 3	-1.227327	**0.009703**	0.098211	-1.108939	0.094105	0.709305
Integrin alpha-IIb: beta-3 complex	-1.227568	**0.012048**	0.098211	-1.14251	0.069852	0.709305
Extracellular matrix protein 1	-1.227708	**0.026138**	0.11038	-1.077997	0.184554	0.709305
RGM domain family member B	-1.227927	**0.015719**	0.101247	-1.0541	0.480149	0.802297
E-selectin	-1.230377	**0.013881**	0.099154	-1.093508	0.18042	0.709305
Coagulation factor IX	-1.231931	**0.000469**	0.096991	-1.115706	0.074125	0.709305
C-C motif chemokine 28	-1.234223	**0.009213**	0.098211	-1.008191	0.905619	0.964952
Non-receptor tyrosine-protein kinase TYK2	-1.23459	**0.047636**	0.13811	-1.348914	**0.018834**	0.709305
Tumor necrosis factor receptor superfamily member 14	-1.234926	**0.006397**	0.098123	-1.117583	0.092656	0.709305
Hepatocyte growth factor receptor	-1.236735	**0.00992**	0.098211	-1.128297	0.076585	0.709305
Complement C1q subcomponent	-1.237833	**0.035538**	0.122368	1.168939	0.281414	0.711716
Epidermal growth factor receptor	-1.240504	**0.024393**	0.108275	-1.08605	0.231667	0.709305
Platelet glycoprotein 4	-1.241027	**0.040233**	0.127697	-1.041811	0.60598	0.849515
Appetite-regulating hormone	-1.244916	**0.009025**	0.098211	-1.079673	0.285454	0.71279
Granulins	-1.245313	**0.011398**	0.098211	-1.118569	0.109444	0.709305
Insulin-like growth factor-binding protein 2	-1.245425	**0.024223**	0.108271	-1.101166	0.24135	0.709305
WAP, kazal, immunoglobulin, kunitz and NTR domain-containing protein 1	-1.245928	**0.006904**	0.098123	-1.094872	0.164342	0.709305
Tumor necrosis factor ligand superfamily member 13B	-1.246363	**0.020483**	0.106496	-1.183533	**0.017366**	0.709305
3-hydroxyisobutyrate dehydrogenase, mitochondrial	-1.246372	**0.010966**	0.098211	-1.102213	0.258176	0.709305
Stromal cell-derived factor 1	-1.247023	**0.003835**	0.098123	-1.103343	0.145969	0.709305
Mediator of RNA polymerase II transcription subunit 1	-1.247699	**0.016612**	0.101247	-1.052059	0.338678	0.716341
Neutrophil gelatinase-associated lipocalin	-1.247995	**0.017196**	0.101247	-1.049711	0.244078	0.709305
Granzyme A	-1.248121	**0.020388**	0.106459	-1.111213	0.124148	0.709305
Interferon beta	-1.248357	**0.008335**	0.098123	-1.130946	**0.025012**	0.709305
Apolipoprotein E (isoform E3)	-1.248374	**0.014108**	0.099154	-1.028736	0.522871	0.828815
Mannose-binding protein C	-1.248517	**0.003572**	0.098123	-1.18181	**0.007593**	0.709305
Dickkopf-related protein 3	-1.251875	**0.031814**	0.119301	1.002226	0.97441	0.992666
Collectin-11	-1.252265	**0.010823**	0.098211	-1.126814	0.158282	0.709305
Sialic acid-binding Ig-like lectin 9	-1.253201	**0.024018**	0.108271	-1.118626	0.162845	0.709305
Collagenase 3	-1.254623	**0.019951**	0.106459	-1.176611	0.146016	0.709305
Interleukin-17 receptor C	-1.257482	**0.018081**	0.10228	-1.127257	0.132851	0.709305
Bone morphogenetic protein 10	-1.258623	**0.003716**	0.098123	-1.109278	0.067051	0.709305
Galectin-3-binding protein	-1.25983	**0.00827**	0.098123	-1.06949	0.280742	0.711716
Semaphorin-3E	-1.26052	**0.008022**	0.098123	-1.084464	0.387818	0.757639
Noggin	-1.261772	**0.020394**	0.106459	-1.102504	0.20297	0.709305
Protein Z-dependent protease inhibitor	-1.262019	**0.010199**	0.098211	-1.149021	0.057811	0.709305
ADP-ribosyl cyclase/cyclic ADP-ribose hydrolase 2	-1.262147	**0.035206**	0.122139	-1.113998	0.216395	0.709305
Interleukin-17 receptor B	-1.262148	**0.011531**	0.098211	-1.119063	0.157554	0.709305
Afamin	-1.262503	**0.006567**	0.098123	-1.072556	0.177372	0.709305
Retinoic acid receptor responder protein 2	-1.263604	**0.006658**	0.098123	-1.106438	0.15193	0.709305
72 kDa type IV collagenase	-1.264007	**0.016314**	0.101247	-1.147465	0.082282	0.709305
Chromogranin-A	-1.266723	**0.005252**	0.098123	-1.191664	0.09884	0.709305
Plasma protease C1 inhibitor	-1.266953	**0.013017**	0.098619	-1.09458	0.177803	0.709305
Inter-alpha-trypsin inhibitor heavy chain H4	-1.267822	**0.012578**	0.098211	-1.098973	**0.013081**	0.709305

	Control T24 vs T0			SlpD T24 vs T0		
**Protein**	**Fold Change**	**p-value**	**adj. p-value**	**Fold Change**	**p-value**	**adj. p-value**
Midkine	-1.2687	**0.042679**	0.13	-1.106306	0.319386	0.713675
Cystatin-M	-1.270401	**0.003405**	0.098123	-1.134733	0.089978	0.709305
Osteomodulin	-1.271753	**0.021186**	0.107437	-1.056908	0.448654	0.783794
Endostatin	-1.274077	**0.021805**	0.108197	-1.06437	0.239313	0.709305
Thrombospondin-2	-1.275343	**0.039634**	0.127353	1.159038	0.175374	0.709305
Plasma kallikrein	-1.276338	**0.009621**	0.098211	-1.094152	0.070933	0.709305
Trefoil factor 3	-1.277207	**0.002721**	0.098123	-1.037907	0.630261	0.85587
Cathepsin H	-1.277543	**0.024753**	0.109132	-1.034132	0.718849	0.900882
Ephrin-A4	-1.278773	**0.011971**	0.098211	-1.104281	0.237987	0.709305
Complement factor B	-1.278944	**0.005768**	0.098123	-1.043099	0.315346	0.713675
Serum amyloid P-component	-1.27922	**0.002919**	0.098123	-1.054916	0.309482	0.713675
Ciliary neurotrophic factor receptor subunit alpha	-1.279297	**0.024143**	0.108271	-1.08907	0.330314	0.713675
Leukotriene A-4 hydrolase	-1.279621	**0.024051**	0.108271	-1.189061	0.065114	0.709305
Kunitz-type protease inhibitor 1	-1.279712	**0.031395**	0.118846	-1.097567	0.338308	0.716341
Interleukin-19	-1.280043	**0.005494**	0.098123	-1.146822	0.055322	0.709305
Apolipoprotein L1	-1.280343	**0.006189**	0.098123	-1.067201	0.306648	0.713675
Somatostatin-28	-1.280915	**0.016959**	0.101247	-1.106642	0.115985	0.709305
Cytochrome P450 3A4	-1.281197	**0.007635**	0.098123	-1.120494	**0.030075**	0.709305
Urokinase plasminogen activator surface receptor	-1.28141	**0.012719**	0.098211	-1.049066	0.554998	0.835896
Amphoterin-induced protein 2	-1.281962	**0.029001**	0.114686	-1.117269	0.21076	0.709305
Persulfide dioxygenase ETHE1, mitochondrial	-1.283581	**0.001443**	0.098123	-1.164853	0.071042	0.709305
Carbohydrate sulfotransferase 15	-1.283644	**0.008625**	0.098123	-1.089871	0.216989	0.709305
Wnt inhibitory factor 1	-1.284294	**0.010312**	0.098211	-1.075627	0.397221	0.760394
Interleukin-2	-1.284728	**0.026059**	0.11038	-1.095518	0.437417	0.78303
Endothelial cell-specific molecule 1	-1.285598	**0.00134**	0.098123	-1.094298	0.149996	0.709305
Fibronectin Fragment 4	-1.287633	**0.032936**	0.120328	1.0014	0.988542	1
C-type lectin domain family 4 member M	-1.288506	**0.007834**	0.098123	-1.10783	**0.045124**	0.709305
Tyrosine-protein phosphatase non-receptor type substrate 1	-1.288793	**0.017301**	0.101247	-1.132579	0.186693	0.709305
Kininogen-1	-1.289852	**0.005552**	0.098123	-1.175646	**0.002248**	0.489037
Cation-independent mannose-6-phosphate receptor	-1.289947	**0.018807**	0.103223	-1.142351	0.070909	0.709305
Tissue factor pathway inhibitor	-1.290707	**0.015616**	0.101247	-1.121678	0.087029	0.709305
Ephrin-A5	-1.292124	**0.020097**	0.106459	-1.1129	0.273383	0.711716
Insulin-like growth factor 1 receptor	-1.292134	**0.032993**	0.120328	-1.059383	0.543633	0.83399
Ephrin type-B receptor 2	-1.293592	**0.012608**	0.098211	-1.071495	0.416853	0.767268
Low affinity immunoglobulin epsilon Fc receptor	-1.29399	**0.003188**	0.098123	-1.091012	0.165151	0.709305
Quinone oxidoreductase-like protein 1	-1.294038	**0.008265**	0.098123	-1.230605	**0.037973**	0.709305
gp41 C34 peptide, HIV	-1.294829	**0.012559**	0.098211	-1.170384	0.091803	0.709305
Leucine-rich repeat transmembrane protein FLRT3	-1.295221	**0.032408**	0.120297	-1.084208	0.429428	0.77726
Thymidine kinase, cytosolic	-1.296047	**0.03392**	0.120538	-1.107397	0.11966	0.709305
Aspartate aminotransferase, cytoplasmic	-1.297111	**0.007478**	0.098123	-1.11837	0.099056	0.709305
Tumor necrosis factor ligand superfamily member 12	-1.299547	**0.007743**	0.098123	-1.104487	0.185837	0.709305
Macrophage mannose receptor 1	-1.300045	**0.004449**	0.098123	-1.12133	0.144054	0.709305
Insulin-like growth factor-binding protein 7	-1.301535	**0.033804**	0.120531	-1.11488	0.160268	0.709305
Carboxypeptidase E	-1.302028	**0.004468**	0.098123	-1.105875	0.159618	0.709305
Tissue Factor	-1.302694	**0.022168**	0.108271	-1.163667	0.108363	0.709305
GTPase KRas	-1.303101	**0.003208**	0.098123	-1.094568	0.176321	0.709305
Transforming growth factor-beta-induced protein ig-h3	-1.30394	**0.028415**	0.114408	-1.128458	0.081962	0.709305
Diablo homolog, mitochondrial	-1.303998	**0.018877**	0.103223	-1.158818	0.099635	0.709305
Ephrin-B2	-1.305841	**0.030454**	0.116726	-1.066743	0.521139	0.828363
Dual specificity mitogen-activated protein kinase kinase 4	-1.307068	**0.027243**	0.112489	-1.169092	**0.002954**	0.550622
Natural cytotoxicity triggering receptor 1	-1.307395	**0.008298**	0.098123	-1.139626	0.072414	0.709305
Protein S100-A9	-1.30893	**0.028854**	0.114651	-1.078256	0.720095	0.900882
Leucine-rich repeats and immunoglobulin-like domains protein 3	-1.309081	**0.010706**	0.098211	-1.11086	0.306597	0.713675
Myeloid cell surface antigen CD33	-1.309593	**0.016643**	0.101247	-1.110231	0.284857	0.71279
Nidogen-2	-1.314335	**0.000874**	0.096991	-1.222063	**0.007644**	0.709305
C-C motif chemokine 16	-1.318921	**0.00404**	0.098123	-1.062723	0.44679	0.783794
Kallistatin	-1.320073	**0.004212**	0.098123	-1.112337	**0.039958**	0.709305
Alpha-(1,3)-fucosyltransferase 5	-1.324265	**0.018024**	0.10228	-1.12872	0.207241	0.709305
Eotaxin	-1.325074	**0.014676**	0.099154	-1.061435	0.505964	0.823077
Nidogen-1	-1.326703	**0.002702**	0.098123	-1.185697	**0.014829**	0.709305
GDNF family receptor alpha-1	-1.327455	**0.013368**	0.099136	-1.091699	0.283287	0.71279
Insulin receptor	-1.328709	**0.010018**	0.098211	-1.075164	0.418415	0.769058
Bone morphogenetic protein 6	-1.329167	**0.020914**	0.107373	-1.040045	0.665088	0.87926
Leucine-rich repeat transmembrane protein FLRT2	-1.330095	**0.019615**	0.105991	-1.097864	0.312468	0.713675
Human Chorionic Gonadotropin	-1.330835	**0.039643**	0.127353	-1.085479	0.438359	0.783156
Ectonucleotide pyrophosphatase/phosphodiesterase family member 7	-1.335079	**0.005066**	0.098123	-1.113339	0.231104	0.709305
Parathyroid hormone	-1.342763	**0.030432**	0.116726	-1.279525	0.164578	0.709305
Apolipoprotein M	-1.344005	**0.010739**	0.098211	-1.161215	0.083385	0.709305
Superoxide dismutase [Mn], mitochondrial	-1.345917	**0.005869**	0.098123	-1.105837	0.10388	0.709305
Muellerian-inhibiting factor	-1.348867	**0.006356**	0.098123	-1.192917	**0.027281**	0.709305
Ephrin type-A receptor 2	-1.357023	**0.011882**	0.098211	-1.101462	0.32439	0.713675
C-X-C motif chemokine 16	-1.357239	**0.001041**	0.096991	-1.094968	0.234345	0.709305
Plasma serine protease inhibitor	-1.361434	**0.000312**	0.096991	-1.364023	0.122371	0.709305
SLAM family member 7	-1.364797	**0.02415**	0.108271	-1.073689	0.366703	0.738499
Pappalysin-1	-1.369629	**0.000242**	0.096991	-1.247284	**0.001451**	0.378768
Immunoglobulin D	-1.369634	**0.003733**	0.098123	-1.168379	**0.024881**	0.709305
Inosine-5'-monophosphate dehydrogenase 1	-1.375205	**0.020372**	0.106459	-1.028196	0.754235	0.916459
Cystatin-SA	-1.376404	**0.014787**	0.099154	-1.232491	0.167781	0.709305
Cerebral dopamine neurotrophic factor	-1.378627	**0.007226**	0.098123	-1.157528	0.113996	0.709305
Histone H2A.z	-1.379213	**0.003359**	0.098123	1.056023	0.772672	0.923142
Leukemia inhibitory factor receptor	-1.381113	**0.005041**	0.098123	-1.159528	0.147252	0.709305
Interleukin-1 receptor type 1	-1.382573	**0.014828**	0.099154	-1.106612	0.29401	0.713675
Endoplasmic reticulum aminopeptidase 1	-1.38925	**0.008859**	0.098211	-1.027054	0.74085	0.911224
Vitronectin	-1.389474	**0.002576**	0.098123	-1.205593	**0.018477**	0.709305
Secreted frizzled-related protein 1	-1.394194	**0.024211**	0.108271	-1.237489	0.068234	0.709305
Erythropoietin	-1.394513	**0.038615**	0.126298	1.005134	0.966333	0.988962
Interleukin-18 receptor 1	-1.395774	**0.005806**	0.098123	-1.08368	0.336004	0.716341
C-C motif chemokine 23	-1.399791	**0.004017**	0.098123	-1.24965	**0.025372**	0.709305
Trefoil factor 1	-1.400331	**0.009965**	0.098211	-1.128995	0.244964	0.709305
C-X-C motif chemokine 13	-1.403693	**0.0034**	0.098123	-1.165802	0.182448	0.709305
Cystatin-D	-1.407571	**0.00323**	0.098123	-1.242668	0.150492	0.709305
Apolipoprotein B	-1.412979	**0.011965**	0.098211	-1.047765	0.614288	0.849515
Semaphorin-5A	-1.41408	**0.0043**	0.098123	-1.118042	0.20137	0.709305
Tumor necrosis factor-inducible gene 6 protein	-1.414408	**0.000353**	0.096991	-1.202339	**0.020134**	0.709305
Matrix metalloproteinase-9	-1.414641	0.082175	0.176089	1.714254	**0.044366**	0.709305
Troponin I, fast skeletal muscle	-1.433633	**0.043562**	0.130987	-1.110158	0.416483	0.767268
High affinity cGMP-specific 3',5'-cyclic phosphodiesterase 9A	-1.435265	**0.001004**	0.096991	-1.209579	**0.021789**	0.709305
Sclerostin	-1.438671	**0.000769**	0.096991	-1.208468	0.061327	0.709305
Chitinase-3-like protein 1	-1.452757	**0.001857**	0.098123	-1.07686	0.406062	0.766876
Osteopontin	-1.505412	**0.006756**	0.098123	-1.129217	0.375448	0.744153
Interleukin-18-binding protein	-1.507299	**0.002062**	0.098123	-1.245892	**0.02422**	0.709305
Bone sialoprotein 2	-1.507565	**0.001413**	0.098123	-1.3455	**0.027697**	0.709305
Lactadherin	-1.545652	**0.049887**	0.141173	-1.137779	0.606015	0.849515
Ephrin type-A receptor 1	-1.571233	**0.006852**	0.098123	-1.229806	0.165112	0.709305
N-terminal pro-BNP	-1.749812	**0.009632**	0.098211	1.062166	0.709534	0.898101
Complement C3b	-1.786915	**0.010555**	0.098211	-1.125492	0.468044	0.794636
Hepcidin	-1.828162	**0.018904**	0.103223	-2.079924	**0.000206**	0.269027
Ck-beta-8-1	-1.8451	**0.00054**	0.096991	-1.21713	0.060116	0.709305
Histone H2B type 2-E	-2.123864	**0.005148**	0.098123	-1.100443	0.696015	0.893497
Haptoglobin	-3.233298	0.055211	0.146945	-1.307002	**0.031627**	0.709305
Olfactomedin-4	-3.89608	**0.016972**	0.101247	1.566602	0.298026	0.713675

Supplementary Figure 8. Plasma proteins with significant fold change following control night and acute sleep deprivation, side by side comparison. Proteins are organized by descending value of fold change in control samples. Tables are a continuation from left to right. Bolded in red are the significant values p<0.05. Of note none of the protein fold change had a significant adjusted p-value.SlpD=sleep deprived. T24=Hour 24, T0= Hour 0.

Protein	Control T24 vs T0 Fold Change	Control T24 vs T0 p-value	Control T24 vs T0 adj. p-value	SlpD T24 vs T0 Fold Change	SlpD T24 vs T0 p-value	SlpD T24 vs T0 adj. p-value
Hemoglobin	5.445114	**0.004877**	0.098123	1.446468	0.181927	0.709305
Carbonic anhydrase 3	2.427809	**0.013614**	0.099154	1.350113	0.165627	0.709305
NSFL1 cofactor p47	2.309426	**0.010738**	0.098211	1.362604	0.11378	0.709305
Ubiquitin	2.255690	**0.003656**	0.098123	-1.976762	**0.021223**	0.709305
Protein 4.1	2.238844	**0.01216**	0.098211	1.434799	0.129798	0.709305
Malate dehydrogenase, cytoplasmic	2.205248	**0.012347**	0.098211	1.342183	0.067137	0.709305
Rab GDP dissociation inhibitor beta	2.194540	**0.023876**	0.108271	1.362748	0.123128	0.709305
Serine/threonine-protein kinase WNK3	2.088700	**0.021077**	0.107437	1.368448	0.196629	0.709305
Aflatoxin B1 aldehyde reductase member 2	2.063057	**0.04164**	0.129073	1.242280	0.472793	0.796123
Fibroblast growth factor 8 isoform A	1.988496	**0.028502**	0.114408	1.260311	0.233698	0.709305
Heterogeneous nuclear ribonucleoprotein A/B	1.965525	**0.022776**	0.108271	1.262478	0.297451	0.713675
SUMO-conjugating enzyme UBC9	1.952166	**0.037528**	0.1256	1.290152	0.252124	0.709305
Interferon lambda-1	1.932702	**0.026794**	0.112071	1.233411	0.101151	0.709305
Desert hedgehog protein N-product	1.913464	**0.02758**	0.113137	1.258356	0.136943	0.709305
CD40 ligand	1.899174	**0.016128**	0.101247	1.178389	0.118898	0.709305
Proteasome subunit alpha type-6	1.883612	**0.04335**	0.130649	1.266797	0.164953	0.709305
DnaJ homolog subfamily B member 1	1.880522	**0.042602**	0.13	1.387991	0.079771	0.709305
Proteasome activator complex subunit 1	1.862498	**0.039688**	0.127353	1.178540	0.243535	0.709305
Fibroblast growth factor 6	1.855325	**0.027077**	0.112489	1.188767	0.156597	0.709305
Alcohol dehydrogenase [NADP(+)]	1.839176	**0.014726**	0.099154	1.288616	**0.04753**	0.709305
Ubiquitin-like protein ISG15	1.830138	**0.028668**	0.114408	1.194721	0.127738	0.709305
AT-rich interactive domain-containing protein 3A	1.829495	**0.035673**	0.122509	1.250914	0.140076	0.709305
Small ubiquitin-related modifier 3	1.812233	**0.04731**	0.137505	1.233458	0.178046	0.709305
Ubiquitin-fold modifier 1	1.810410	**0.022682**	0.108271	1.321884	0.188356	0.709305
Kremen protein 2	1.799080	**0.016864**	0.101247	1.168322	0.130803	0.709305
S-formylglutathione hydrolase	1.797249	**0.022839**	0.108271	1.237084	0.173098	0.709305
Dual specificity mitogen-activated protein kinase kinase 3	1.796040	**0.039651**	0.127353	1.174606	0.176571	0.709305
Apoptosis regulator Bcl-2	1.787859	**0.045651**	0.134479	1.254415	0.113108	0.709305
Glucocorticoid receptor	1.785956	**0.011334**	0.098211	1.030864	0.815447	0.933932
Eukaryotic initiation factor 4A-III	1.781463	**0.005838**	0.098123	1.088673	0.307732	0.713675
Angiotensin-converting enzyme 2	1.774739	**0.013266**	0.099136	1.126636	0.183145	0.709305
Ribosomal protein S6 kinase alpha-3	1.756809	**0.017197**	0.101247	1.268653	0.193805	0.709305
Vasoactive Intestinal Peptide	1.754076	**0.032559**	0.120297	1.214302	0.081634	0.709305
Casein kinase II 2-alpha':2-beta heterotetramer	1.751835	**0.013074**	0.098619	1.187842	0.145286	0.709305
Calcium/calmodulin-dependent protein kinase kinase 1	1.709649	**0.015994**	0.101247	1.057423	0.537682	0.83399
cGMP-dependent 3',5'-cyclic phosphodiesterase	1.699727	**0.006433**	0.098123	1.011299	0.863424	0.948903
Leukocyte surface antigen CD47	1.698734	**0.025914**	0.11038	1.180791	0.071812	0.709305
Fibroblast growth factor 7	1.694894	**0.024128**	0.108271	1.106530	0.325871	0.713675
Alpha-enolase	1.693679	**0.04842**	0.139489	1.180834	0.414721	0.767268
Peptidyl-prolyl cis-trans isomerase A	1.690459	**0.045233**	0.13355	1.285175	0.235877	0.709305
Heat shock protein HSP 90-alpha/beta	1.687027	**0.011424**	0.098211	1.180583	0.256205	0.709305
Peptidyl-prolyl cis-trans isomerase E	1.665334	**0.04773**	0.13811	1.204564	0.228276	0.709305
Arginase-1	1.661083	**0.007795**	0.098123	1.093947	0.245262	0.709305
Adapter molecule crk	1.660790	**0.008245**	0.098123	1.276064	**0.049399**	0.709305
Mesothelin	1.655710	**0.007389**	0.098123	1.129831	0.104171	0.709305
Heat shock protein beta-1	1.642786	**0.033655**	0.120328	1.207016	0.3926	0.759027
NKG2D ligand 3	1.641930	**0.029548**	0.116142	1.154202	0.142931	0.709305
14-3-3 protein epsilon	1.634393	**0.015458**	0.100862	1.033320	0.831699	0.941467
Receptor-type tyrosine-protein kinase FLT3	1.632408	**0.012542**	0.098211	1.166038	0.146424	0.709305
Mitogen-activated protein kinase 1	1.622529	**0.036474**	0.123749	1.171222	0.412202	0.767268
Tyrosine-protein phosphatase non-receptor type 11	1.622304	**0.033534**	0.120328	1.204084	0.194082	0.709305
Azurocidin	1.617306	**0.024226**	0.108271	1.119336	0.277944	0.711716
Vascular endothelial growth factor C	1.614752	**0.028633**	0.114408	1.139361	0.1062	0.709305
Pyridoxal kinase	1.611492	**0.003232**	0.098123	1.190557	**0.045891**	0.709305
Galectin-4	1.608304	**0.009265**	0.098211	1.112232	0.085653	0.709305
Histone acetyltransferase type B catalytic subunit	1.594279	**0.0334**	0.120328	1.117552	0.277402	0.711716
Methyl-CpG-binding domain protein 4	1.591293	**0.011372**	0.098211	1.107297	0.192016	0.709305
Glucose-6-phosphate isomerase	1.589937	**0.007889**	0.098123	1.128915	0.412087	0.767268
Leucine-rich repeat transmembrane protein FLRT1	1.588822	**0.04297**	0.130105	1.125978	0.18917	0.709305
Heat shock protein HSP 90-beta	1.588802	**0.010236**	0.098211	1.165514	0.232524	0.709305
Keratin, type I cytoskeletal 18	1.584276	**0.013559**	0.099154	1.118438	0.101478	0.709305
Ribosomal protein S6 kinase alpha-5	1.578419	**0.040941**	0.127697	1.107205	0.325003	0.713675
N-acetyl-D-glucosamine kinase	1.576850	**0.005909**	0.098123	1.161893	0.120747	0.709305
Calpain I	1.573110	**0.013915**	0.099154	1.232713	0.193779	0.709305
Macrophage migration inhibitory factor	1.561510	**0.025557**	0.11038	1.034912	0.602401	0.849393
L-lactate dehydrogenase B chain	1.558891	**0.008107**	0.098123	1.135386	**0.017295**	0.709305
Cytotoxic and regulatory T-cell molecule	1.557695	**0.022404**	0.108271	1.111079	0.14696	0.709305
Cyclin-dependent kinase 1:G2/mitotic-specific cyclin-B1 complex	1.554883	**0.014415**	0.099154	1.103420	0.089612	0.709305
Erythropoietin receptor	1.551583	**0.017798**	0.10228	1.088009	0.10598	0.709305
Kynureninase	1.539486	**0.003282**	0.098123	1.119747	**0.015803**	0.709305
Calcium/calmodulin-dependent 3',5'-cyclic nucleotide phosphodiesterase 1A	1.531621	**0.040799**	0.127697	1.134032	0.146024	0.709305
Fructose-bisphosphate aldolase A	1.530237	**0.007927**	0.098123	1.205241	0.093775	0.709305
Tumor-associated calcium signal transducer 2	1.525770	**0.012483**	0.098211	1.056701	0.229699	0.709305
Chloride intracellular channel protein 1	1.511716	**0.032268**	0.120297	1.187251	0.431423	0.777634
40S ribosomal protein S3a	1.510099	**0.045046**	0.1333	1.104266	0.525826	0.828815
Growth/differentiation factor 11/8	1.509886	**0.00015**	0.096991	1.140399	**0.024818**	0.709305
Xaa-Pro aminopeptidase 1	1.501625	**0.023259**	0.108271	1.198040	0.194341	0.709305
Thymic stromal lymphopoietin	1.493445	**0.021693**	0.108197	1.121224	0.099945	0.709305
DNA topoisomerase 1	1.490343	**0.044965**	0.1333	1.204414	0.479402	0.802076
Proteasome subunit alpha type-1	1.468599	**0.027325**	0.112489	1.055977	0.278709	0.711716
40S ribosomal protein S7	1.467145	**0.042439**	0.13	1.213432	0.44721	0.783794
Fibroblast growth factor 4	1.457990	**0.006399**	0.098123	1.088983	0.17731	0.709305
CD63 antigen	1.450920	**0.009393**	0.098211	1.030653	0.593038	0.847662
Cathepsin D	1.291786	0.395739	0.498975	-1.885102	**0.001245**	0.378768
Netrin receptor UNC5D	1.022354	0.894137	0.919503	1.968520	**0.000591**	0.307242
Thyroid Stimulating Hormone	-1.187705	0.184881	0.293112	-1.524207	**0.005781**	0.628677
Matrix metalloproteinase-9	-1.414641	0.082175	0.176089	1.714254	**0.044366**	0.709305
Chitinase-3-like protein 1	-1.452757	**0.001857**	0.098123	-1.076860	0.406062	0.766876
Osteopontin	-1.505412	**0.006756**	0.098123	-1.129217	0.375448	0.744153
Interleukin-18-binding protein	-1.507299	**0.002062**	0.098123	-1.245892	**0.02422**	0.709305
Bone sialoprotein 2	-1.507565	**0.001413**	0.098123	-1.345500	**0.027697**	0.709305
Lactadherin	-1.545652	**0.049887**	0.141173	-1.137779	0.606015	0.849515
Ephrin type-A receptor 1	-1.571233	**0.006852**	0.098123	-1.229806	0.165112	0.709305
N-terminal pro-BNP	-1.749812	**0.009632**	0.098211	1.062166	0.709534	0.898101
Complement C3b	-1.786915	**0.010555**	0.098211	-1.125492	0.468044	0.794636
Hepcidin	-1.828162	**0.018904**	0.103223	-2.079924	**0.000206**	0.269027
Ck-beta-8-1	-1.845100	**0.00054**	0.096991	-1.217130	0.060116	0.709305
Histone H2B type 2-E	-2.123864	**0.005148**	0.098123	-1.100443	0.696015	0.893497
Olfactomedin-4	-3.896080	**0.016972**	0.101247	1.566602	0.298026	0.713675

Fold Change: Up — 1.5 — -1.5 — Down

Supplementary Figure 9. Plasma proteins with fold change ≥1.5 following control night and acute sleep deprivation, side by side comparison. Proteins are organized by descending value of fold change in control samples. Protein fold change is colored by directionality of 1.5 fold change as follows: blue=upregulated, red=downregulated, and white= less than 1.5 fold change on either direction or no significant change. The color gradient represents magnitude of fold change. Bolded in red are the significant values p<0.05. Of note none of the protein fold change had a significant adjusted p-value. SlpD=sleep deprived. T24=Hour 24, T0= Hour 0.

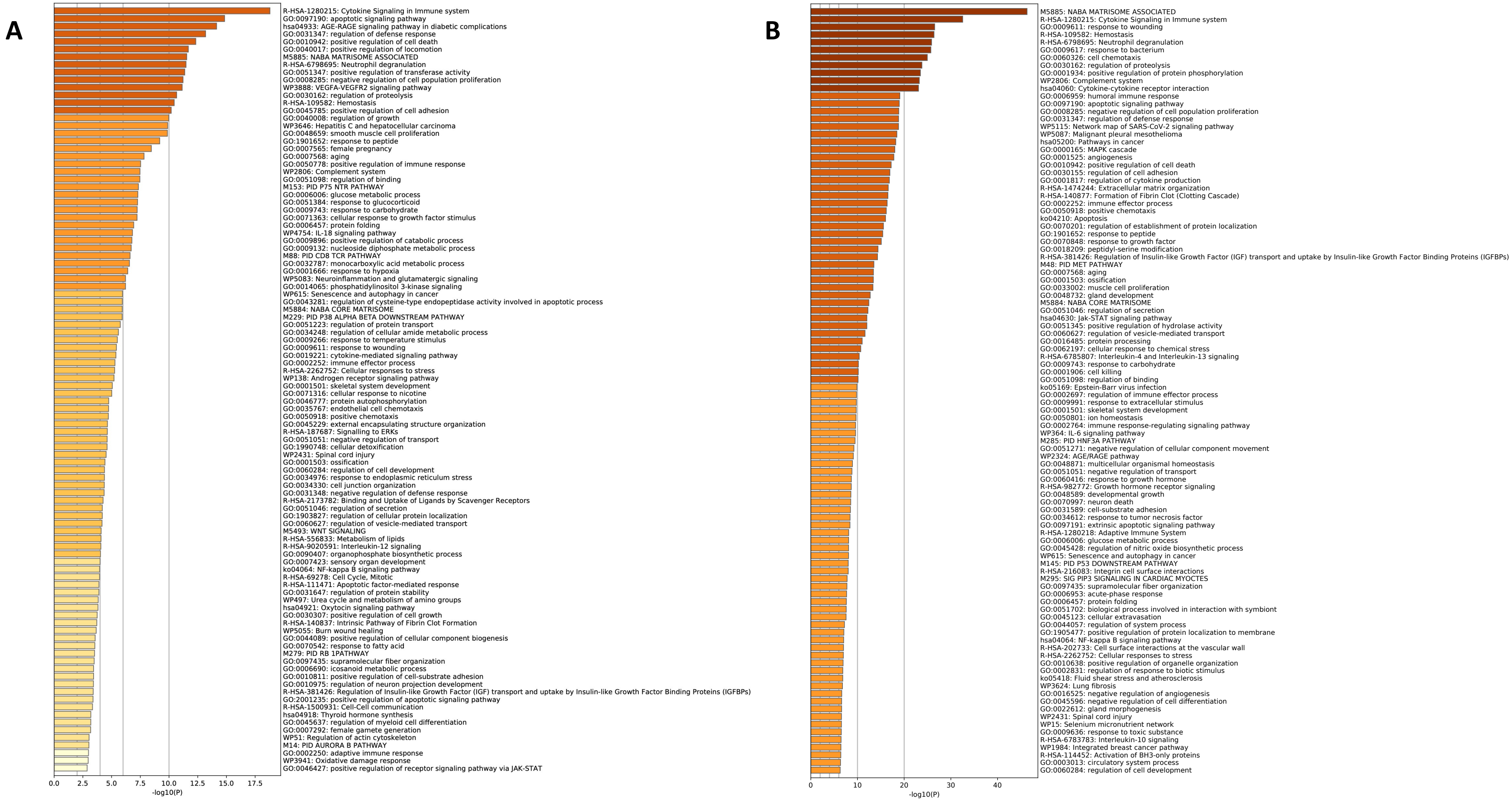

Supplementary Figure 10. Bar graph of top 100 upregulated enriched terms across input gene list in CSF samples of control (A) and acutely sleep deprived subjects (B) at hour 24 vs hour 0. Colored by p-value.

A

GO	Category	Description	Count	%	Log10(P)	Log10(q)
R-HSA-1280215	Reactome Gene Sets	Cytokine Signaling in Immune system	30	20.13	-18.79	-14.43
GO:0097190	GO Biological Processes	apoptotic signaling pathway	24	16.11	-14.84	-10.78
hsa04933	KEGG Pathway	AGE-RAGE signaling pathway in diabetic complications	13	8.72	-14.14	-10.26
GO:0031347	GO Biological Processes	regulation of defense response	23	15.44	-13.18	-9.43
GO:0010942	GO Biological Processes	positive regulation of cell death	22	14.77	-12.33	-8.88
GO:0040017	GO Biological Processes	positive regulation of locomotion	21	14.09	-11.68	-8.44
M5885	Canonical Pathways	NABA MATRISOME ASSOCIATED	23	15.44	-11.54	-8.39
R-HSA-6798695	Reactome Gene Sets	Neutrophil degranulation	19	12.75	-11.48	-8.35
GO:0051347	GO Biological Processes	positive regulation of transferase activity	20	13.42	-11.37	-8.27
GO:0008285	GO Biological Processes	negative regulation of cell population proliferation	23	15.44	-11.22	-8.17
WP3888	WikiPathways	VEGFA-VEGFR2 signaling pathway	18	12.08	-11.15	-8.13
GO:0030162	GO Biological Processes	regulation of proteolysis	22	14.77	-10.66	-7.75
R-HSA-109582	Reactome Gene Sets	Hemostasis	20	13.42	-10.45	-7.57
GO:0045785	GO Biological Processes	positive regulation of cell adhesion	17	11.41	-10.19	-7.41
GO:0040008	GO Biological Processes	regulation of growth	20	13.42	-9.98	-7.24
WP3646	WikiPathways	Hepatitis C and hepatocellular carcinoma	8	5.37	-9.89	-7.17
GO:0048659	GO Biological Processes	smooth muscle cell proliferation	12	8.05	-9.86	-7.15
GO:1901652	GO Biological Processes	response to peptide	17	11.41	-9.19	-6.58
GO:0007565	GO Biological Processes	female pregnancy	11	7.38	-8.46	-5.98
GO:0007568	GO Biological Processes	aging	13	8.72	-7.83	-5.48

B

Color	MCODE	GO	Description	Log10(P)
	MCODE_1	WP195	IL-1 signaling pathway	-11.0
	MCODE_1	WP3888	VEGFA-VEGFR2 signaling pathway	-10.4
	MCODE_1	WP69	T-cell receptor (TCR) signaling pathway	-10.0
	MCODE_2	M164	PID ERBB1 DOWNSTREAM PATHWAY	-7.4
	MCODE_2	GO:0052548	regulation of endopeptidase activity	-6.6
	MCODE_2	GO:0052547	regulation of peptidase activity	-6.4
	MCODE_3	R-HSA-76002	Platelet activation, signaling and aggregation	-10.5
	MCODE_3	R-HSA-114608	Platelet degranulation	-9.8
	MCODE_3	R-HSA-76005	Response to elevated platelet cytosolic Ca2+	-9.7
	MCODE_5	M50	PID PTP1B PATHWAY	-8.3
	MCODE_5	R-HSA-1280215	Cytokine Signaling in Immune system	-4.9

Supplementary Figure 11. Metascape analyses of CSF samples from control subjects hour 24 vs hour 0 showing upregulated biological pathways and protein interaction networks. Top 20 clusters with their representative enriched terms (A). MCODE components identified in the gene lists of the protein-protein interaction networks (B). See supplementary figure 3 caption for explanation of “count” “%”, “Log10(P)”, and “Log10(q)".

A

GO	Category	Description	Count	%	Log10(P)	Log10(q)
M5885	Canonical Pathways	NABA MATRISOME ASSOCIATED	69	23.71	-46.33	-41.97
R-HSA-1280215	Reactome Gene Sets	Cytokine Signaling in Immune system	55	18.90	-32.54	-28.48
GO:0009611	GO Biological Processes	response to wounding	44	15.12	-26.54	-22.65
R-HSA-109582	Reactome Gene Sets	Hemostasis	46	15.81	-26.39	-22.64
R-HSA-6798695	Reactome Gene Sets	Neutrophil degranulation	41	14.09	-25.87	-22.22
GO:0009617	GO Biological Processes	response to bacterium	49	16.84	-25.72	-22.20
GO:0060326	GO Biological Processes	cell chemotaxis	34	11.68	-24.96	-21.56
GO:0030162	GO Biological Processes	regulation of proteolysis	47	16.15	-23.80	-20.59
GO:0001934	GO Biological Processes	positive regulation of protein phosphorylation	46	15.81	-23.48	-20.33
WP2806	WikiPathways	Complement system	22	7.56	-23.25	-20.16
hsa04060	KEGG Pathway	Cytokine-cytokine receptor interaction	33	11.34	-23.07	-20.03
GO:0006959	GO Biological Processes	humoral immune response	29	9.97	-19.08	-16.21
GO:0097190	GO Biological Processes	apoptotic signaling pathway	37	12.71	-18.95	-16.10
GO:0008285	GO Biological Processes	negative regulation of cell population proliferation	42	14.43	-18.85	-16.02
GO:0031347	GO Biological Processes	regulation of defense response	38	13.06	-18.80	-15.99
WP5115	WikiPathways	Network map of SARS-CoV-2 signaling pathway	25	8.59	-18.79	-15.99
WP5087	WikiPathways	Malignant pleural mesothelioma	32	11.00	-18.44	-15.66
hsa05200	KEGG Pathway	Pathways in cancer	36	12.37	-18.20	-15.43
GO:0000165	GO Biological Processes	MAPK cascade	41	14.09	-18.00	-15.28
GO:0001525	GO Biological Processes	angiogenesis	36	12.37	-17.79	-15.09

B

Color	MCODE	GO	Description	Log10(P)
	MCODE_1	R-HSA-6798695	Neutrophil degranulation	-22.4
	MCODE_1	M5885	NABA MATRISOME ASSOCIATED	-9.1
	MCODE_1	R-HSA-418594	G alpha (i) signalling events	-8.9
	MCODE_2	GO:0002697	regulation of immune effector process	-7.7
	MCODE_2	GO:0032655	regulation of interleukin-12 production	-7.4
	MCODE_2	GO:0032615	interleukin-12 production	-7.4
	MCODE_3	hsa05200	Pathways in cancer	-11.5
	MCODE_3	R-HSA-5663202	Diseases of signal transduction by growth factor receptors and second messengers	-8.9
	MCODE_3	R-HSA-442742	CREB1 phosphorylation through NMDA receptor-mediated activation of RAS signaling	-8.9
	MCODE_4	R-HSA-114608	Platelet degranulation	-35.9
	MCODE_4	R-HSA-76005	Response to elevated platelet cytosolic Ca2+	-35.7
	MCODE_4	R-HSA-76002	Platelet activation, signaling and aggregation	-31.1
	MCODE_5	R-HSA-1280215	Cytokine Signaling in Immune system	-13.3
	MCODE_5	ko04151	PI3K-Akt signaling pathway	-12.2
	MCODE_5	hsa04151	PI3K-Akt signaling pathway	-11.8
	MCODE_6	R-HSA-8957275	Post-translational protein phosphorylation	-17.2
	MCODE_6	R-HSA-381426	Regulation of Insulin-like Growth Factor (IGF) transport and uptake by Insulin-like Growth Factor Binding Proteins (IGFBPs)	-16.8
	MCODE_6	GO:0030510	regulation of BMP signaling pathway	-8.5
	MCODE_7	GO:0033157	regulation of intracellular protein transport	-5.8
	MCODE_7	GO:0032386	regulation of intracellular transport	-5.3
	MCODE_7	GO:0019221	cytokine-mediated signaling pathway	-4.8
	MCODE_8	R-HSA-6785807	Interleukin-4 and Interleukin-13 signaling	-7.4
	MCODE_8	R-HSA-449147	Signaling by Interleukins	-5.5
	MCODE_8	R-HSA-1280215	Cytokine Signaling in Immune system	-4.9

Supplementary Figure 12. Metascape analyses of CSF samples from sleep deprived subjects hour 24 vs hour 0 showing upregulated biological pathways and protein interaction networks. Top 20 clusters with their representative enriched terms (A). MCODE components identified in the gene lists of the protein-protein interaction networks (B). See supplementary figure 3 caption for explanation of “count” “%”, “Log10(P)”, and “Log10(q)".

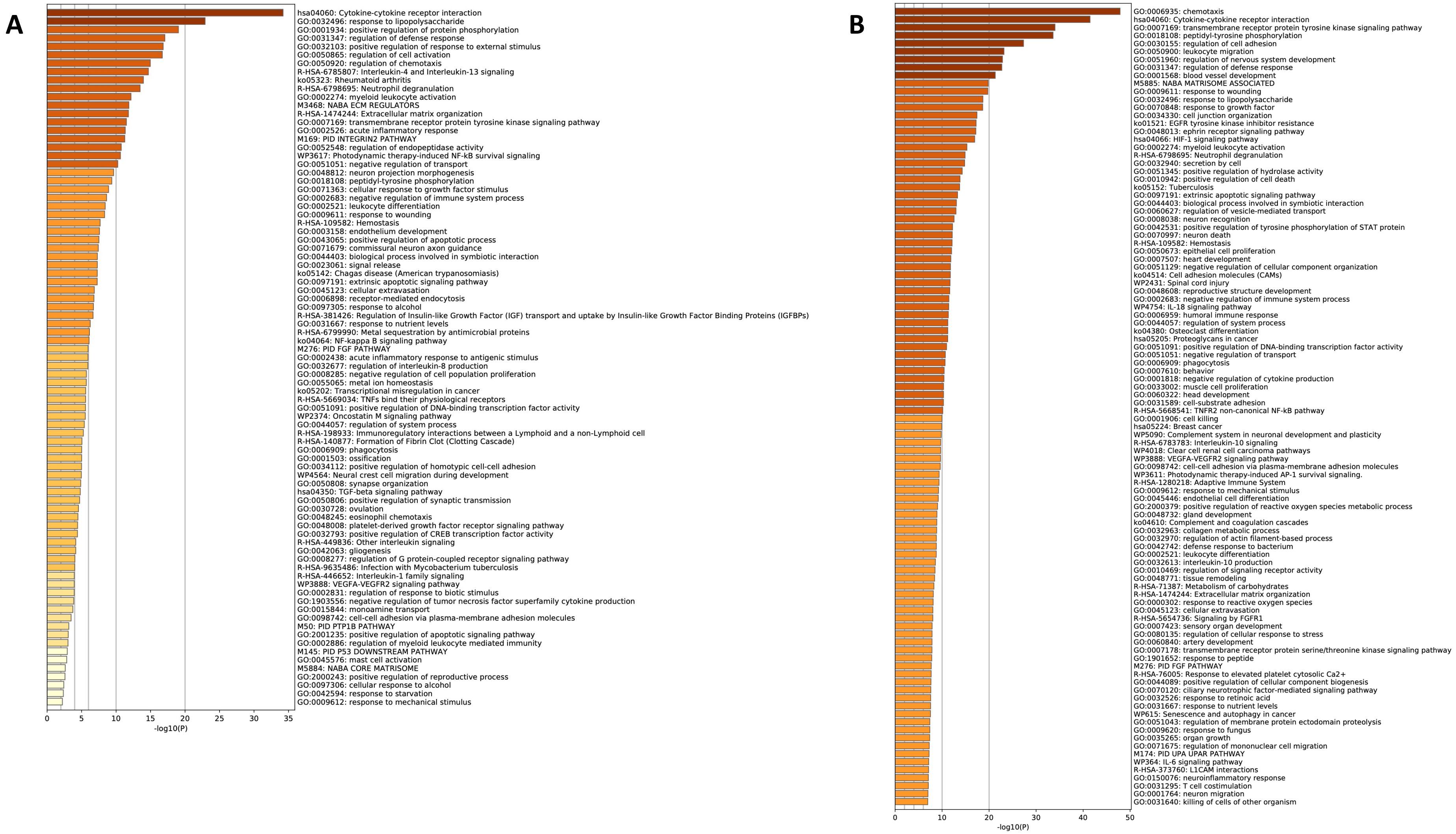

Supplementary Figure 13 . Bar graph of top 100 downregulated enriched terms across input gene list in CSF samples of control (A) and acutely sleep deprived subjects (B) at hour 24 vs hour 0. Colored by p-value.

A

GO	Category	Description	Count	%	Log10(P)	Log10(q)
hsa04060	KEGG Pathway	Cytokine-cytokine receptor interaction	30	29.70	-34.20	-29.84
GO:0032496	GO Biological Processes	response to lipopolysaccharide	23	22.77	-22.90	-19.62
GO:0001934	GO Biological Processes	positive regulation of protein phosphorylation	26	25.74	-19.03	-15.95
GO:0031347	GO Biological Processes	regulation of defense response	23	22.77	-17.07	-14.17
GO:0032103	GO Biological Processes	positive regulation of response to external stimulus	20	19.80	-16.83	-13.97
GO:0050865	GO Biological Processes	regulation of cell activation	23	22.77	-16.70	-13.85
GO:0050920	GO Biological Processes	regulation of chemotaxis	15	14.85	-14.96	-12.22
R-HSA-6785807	Reactome Gene Sets	Interleukin-4 and Interleukin-13 signaling	12	11.88	-14.68	-11.96
ko05323	KEGG Pathway	Rheumatoid arthritis	11	10.89	-13.96	-11.27
R-HSA-6798695	Reactome Gene Sets	Neutrophil degranulation	18	17.82	-13.48	-10.85
GO:0002274	GO Biological Processes	myeloid leukocyte activation	13	12.87	-12.16	-9.62
M3468	Canonical Pathways	NABA ECM REGULATORS	13	12.87	-11.85	-9.34
R-HSA-1474244	Reactome Gene Sets	Extracellular matrix organization	14	13.86	-11.79	-9.29
GO:0007169	GO Biological Processes	transmembrane receptor protein tyrosine kinase signaling pathway	18	17.82	-11.49	-9.01
GO:0002526	GO Biological Processes	acute inflammatory response	10	9.90	-11.33	-8.87
M169	Canonical Pathways	PID INTEGRIN2 PATHWAY	7	6.93	-11.26	-8.80
GO:0052548	GO Biological Processes	regulation of endopeptidase activity	15	14.85	-10.75	-8.35
WP3617	WikiPathways	Photodynamic therapy-induced NF-kB survival signaling	7	6.93	-10.63	-8.23
GO:0051051	GO Biological Processes	negative regulation of transport	15	14.85	-10.24	-7.88
GO:0048812	GO Biological Processes	neuron projection morphogenesis	16	15.84	-9.64	-7.33

B

Color	MCODE	GO	Description	Log10(P)
(red)	MCODE_1	R-HSA-6798695	Neutrophil degranulation	-23.5
(red)	MCODE_1	R-HSA-6803157	Antimicrobial peptides	-14.4
(red)	MCODE_1	GO:0009617	response to bacterium	-11.7
(blue)	MCODE_2	ko04610	Complement and coagulation cascades	-7.8
(blue)	MCODE_2	hsa04610	Complement and coagulation cascades	-7.6
(blue)	MCODE_2	R-HSA-114608	Platelet degranulation	-7.1

Supplementary Figure 14. Metascape analyses of CSF samples from control subjects hour 24 vs hour 0 showing downregulated biological pathways and protein interaction networks. Top 20 clusters with their representative enriched terms (A). MCODE components identified in the gene lists of the protein-protein interaction networks (B). See supplementary figure 3 caption for explanation of “count” “%”, “Log10(P)”, and “Log10(q)".

A

	Category	Description	Count	%	Log10(P)	Log10(q)
GO:0006935	GO Biological Processes	chemotaxis	62	24.22	-47.86	-43.67
hsa04060	KEGG Pathway	Cytokine-cytokine receptor interaction	46	17.97	-41.47	-37.59
GO:0007169	GO Biological Processes	transmembrane receptor protein tyrosine kinase signaling pathway	51	19.92	-34.02	-30.35
GO:0018108	GO Biological Processes	peptidyl-tyrosine phosphorylation	42	16.41	-33.61	-30.03
GO:0030155	GO Biological Processes	regulation of cell adhesion	48	18.75	-27.32	-24.36
GO:0050900	GO Biological Processes	leukocyte migration	33	12.89	-23.16	-20.35
GO:0051960	GO Biological Processes	regulation of nervous system development	35	13.67	-22.84	-20.06
GO:0031347	GO Biological Processes	regulation of defense response	40	15.62	-22.70	-19.93
GO:0001568	GO Biological Processes	blood vessel development	42	16.41	-21.31	-18.62
M5885	Canonical Pathways	NABA MATRISOME ASSOCIATED	40	15.62	-19.81	-17.19
GO:0009611	GO Biological Processes	response to wounding	35	13.67	-19.72	-17.11
GO:0032496	GO Biological Processes	response to lipopolysaccharide	28	10.94	-18.68	-16.12
GO:0070848	GO Biological Processes	response to growth factor	38	14.84	-18.64	-16.09
GO:0034330	GO Biological Processes	cell junction organization	36	14.06	-17.41	-14.94
ko01521	KEGG Pathway	EGFR tyrosine kinase inhibitor resistance	16	6.25	-17.24	-14.79
GO:0048013	GO Biological Processes	ephrin receptor signaling pathway	14	5.47	-17.18	-14.74
hsa04066	KEGG Pathway	HIF-1 signaling pathway	18	7.03	-16.93	-14.50
GO:0002274	GO Biological Processes	myeloid leukocyte activation	21	8.20	-15.27	-12.97
R-HSA-6798695	Reactome Gene Sets	Neutrophil degranulation	28	10.94	-14.89	-12.62
GO:0032940	GO Biological Processes	secretion by cell	35	13.67	-14.79	-12.53

B

Color	MCODE	GO	Description	Log10(P)
(red)	MCODE_1	M181	PID BMP PATHWAY	-9.6
(red)	MCODE_1	hsa04350	TGF-beta signaling pathway	-7.8
(red)	MCODE_1	GO:0030509	BMP signaling pathway	-6.7
(blue)	MCODE_2	hsa05200	Pathways in cancer	-21.8
(blue)	MCODE_2	WP4538	Regulatory circuits of the STAT3 signaling pathway	-17.3
(blue)	MCODE_2	ko04630	Jak-STAT signaling pathway	-16.7
(green)	MCODE_3	GO:0009611	response to wounding	-13.1
(green)	MCODE_3	GO:0018108	peptidyl-tyrosine phosphorylation	-13.1
(green)	MCODE_3	GO:0018212	peptidyl-tyrosine modification	-13.1
(purple)	MCODE_4	R-HSA-3928665	EPH-ephrin mediated repulsion of cells	-28.1
(purple)	MCODE_4	GO:0048013	ephrin receptor signaling pathway	-28.1
(purple)	MCODE_4	R-HSA-2682334	EPH-Ephrin signaling	-25.4
(orange)	MCODE_5	R-HSA-6798695	Neutrophil degranulation	-7.3
(orange)	MCODE_5	WP5055	Burn wound healing	-5.6
(orange)	MCODE_5	GO:0001938	positive regulation of endothelial cell proliferation	-5.6
(yellow)	MCODE_6	GO:0048812	neuron projection morphogenesis	-6.8
(yellow)	MCODE_6	GO:0120039	plasma membrane bounded cell projection morphogenesis	-6.7
(yellow)	MCODE_6	GO:0048858	cell projection morphogenesis	-6.7
(brown)	MCODE_7	R-HSA-1474228	Degradation of the extracellular matrix	-7.0
(brown)	MCODE_7	M3468	NABA ECM REGULATORS	-6.3
(brown)	MCODE_7	R-HSA-1474244	Extracellular matrix organization	-6.0
(pink)	MCODE_8	R-HSA-975634	Retinoid metabolism and transport	-8.5
(pink)	MCODE_8	R-HSA-6806667	Metabolism of fat-soluble vitamins	-8.4
(pink)	MCODE_8	R-HSA-2187338	Visual phototransduction	-7.4

Supplementary Figure 15. Metascape analyses of CSF samples from sleep deprived subjects hour 24 vs hour 0 showing downregulated biological pathways and protein interaction networks. Top 20 clusters with their representative enriched terms (A). MCODE components identified in the gene lists of the protein-protein interaction networks (B). See supplementary figure 3 caption for explanation of “count” “%”, “Log10(P)”, and “Log10(q)".

	Control T24 vs T0			SlpD T24 vs T0		
Protein	Fold Change	p-value	adj. p-value	Fold Change	p-value	adj. p-value
Protein-glutamine gamma-glutamyltransferase E	2.8	**0.02584031**	0.1573649	1.4	0.2814911	0.3907935
Pulmonary surfactant-associated protein D	2.3	**0.009180347**	0.09215656	2.6	**0.01018319**	**0.03451705**
Creatine kinase M-type:Creatine kinase B-type heterodimer	2.2	**0.01157484**	0.101377	1.9	**0.008683787**	**0.03113281**
Integrin alpha-I: beta-1 complex	2.1	**0.02559865**	0.1573649	2.2	0.05810809	0.1296257
ATP synthase subunit O, mitochondrial	2.0	**0.01759954**	0.1279455	1.8	**0.04621486**	0.1087387
Phosphoglycerate mutase 1	2.0	**0.008176638**	0.08605252	1.2	0.468224	0.5836029
Heterogeneous nuclear ribonucleoprotein A/B	1.8	**0.01738077**	0.1274264	1.9	**0.02342029**	0.06475314
Heterogeneous nuclear ribonucleoproteins A2/B1	1.8	**0.02359107**	0.1508924	2.1	**0.02054609**	0.05879965
Pyridoxal kinase	1.8	**0.0022183**	**0.04020669**	1.7	**0.005613548**	**0.0218677**
Mitogen-activated protein kinase 3	1.8	**0.006002103**	0.07138826	1.7	**0.002259928**	**0.0110045**
Pyruvate kinase PKM	1.8	**0.0107818**	0.09637157	2.0	**0.000879555**	**0.00519375**
Tyrosine-protein kinase CSK	1.7	**0.0135307**	0.1124686	1.7	**0.007821048**	**0.02907826**
Fibroblast growth factor 2	1.7	**0.02582264**	0.1573649	1.5	**0.03841014**	0.0947547
Aflatoxin B1 aldehyde reductase member 2	1.7	**0.015618**	0.11919	2.0	**0.000786741**	**0.00470962**
GTP-binding nuclear protein Ran	1.6	**0.0238462**	0.1510645	2.1	**0.000268115**	**0.00205892**
Mothers against decapentaplegic homolog 3	1.6	**0.00081674**	**0.02220511**	1.0	0.9635176	0.9754775
Inorganic pyrophosphatase	1.6	**0.02911335**	0.1681103	1.5	**0.009634974**	**0.03335183**
Protein kinase C alpha type	1.6	**0.01352033**	0.1124686	1.4	**0.03000819**	0.07847832
Creatine kinase M-type	1.6	**0.00963115**	0.09354731	1.3	0.215607	0.3277889
Calcium/calmodulin-dependent protein kinase type II subunit delta	1.6	**0.04731421**	0.220518	1.4	**0.01024188**	**0.03462606**
Non-histone chromosomal protein HMG-14	1.6	**0.007009917**	0.07687346	1.4	**0.009181525**	**0.03212303**
SUMO-conjugating enzyme UBC9	1.6	**0.006402489**	0.07329165	1.6	**0.000510206**	**0.00343206**
N-acetyl-D-glucosamine kinase	1.6	**0.009677308**	0.09354731	1.9	**0.001121042**	**0.00625196**
X-ray repair cross-complementing protein 6	1.6	**0.000284048**	**0.0119575**	1.4	**0.003457303**	**0.01489036**
Tumor necrosis factor ligand superfamily member 12	1.6	**0.01119448**	0.09870808	-1.0	0.8836709	0.9196097
Alcohol dehydrogenase [NADP(+)]	1.6	**0.008697287**	0.08946939	1.4	**0.002274181**	**0.01103274**
Peroxiredoxin-1	1.5	**0.0138137**	0.112668	1.6	**0.005034345**	**0.02002994**
Cyclin-dependent kinase inhibitor 1B	1.5	**3.48143E-06**	**0.000504807**	1.5	**3.95177E-05**	**0.00046882**
Ras-related C3 botulinum toxin substrate 1	1.5	**0.03609412**	0.1894481	1.3	**0.03348133**	0.08467663
Eukaryotic translation initiation factor 4H	1.5	**0.01373826**	0.112668	1.7	**0.001493676**	**0.00789169**
NAD-dependent protein deacetylase sirtuin-2	1.5	**0.001834002**	**0.03626322**	1.2	0.2218054	0.3342448
Ribosome maturation protein SBDS	1.5	**0.00059637**	**0.01853008**	1.5	**1.82699E-05**	**0.00025637**
Phosphoglycerate kinase 1	1.5	**0.000482807**	**0.01615546**	1.6	**0.000247729**	**0.00195931**
Nucleoside diphosphate kinase B	1.5	**0.02740358**	0.1618175	1.5	**0.005840032**	**0.02268227**
Tropomyosin alpha-4 chain	1.5	0.1247793	0.3535328	1.7	**0.01658962**	0.05058284
Sorting nexin-4	1.5	**0.009843724**	0.09445633	1.3	**0.01201069**	**0.03988284**
40S ribosomal protein S7	1.5	**0.01045614**	0.09610876	1.4	**0.001720087**	**0.00887239**
Signal transducer and activator of transcription 6	1.5	**0.03770245**	0.1937643	1.3	0.05324931	0.1216994
ATP-dependent RNA helicase DDX19B	1.5	**0.002405204**	**0.04246425**	1.3	**0.03239029**	0.08304387
Eukaryotic translation initiation factor 5A-1	1.5	0.06038873	0.2443495	1.5	**0.006342163**	**0.02422621**
40S ribosomal protein S3a	1.5	**0.03727269**	0.1930193	1.1	0.2060831	0.3190255
Adenylate kinase isoenzyme 1	1.5	0.08462361	0.2968651	1.4	**0.001813991**	**0.00925732**
Ferritin	1.4	**0.000363629**	**0.01395694**	1.3	**0.01711572**	0.05170375
COMM domain-containing protein 7	1.4	**0.000807054**	**0.02220511**	1.3	**0.003531752**	**0.01511127**
Eukaryotic translation initiation factor 5	1.4	**0.04494577**	0.2117481	1.2	0.2581356	0.3685621
Cadherin-15	1.4	**0.036919**	0.1927172	1.1	0.726351	0.7978856
Aminoacylase-1	1.4	**0.0005213**	**0.01700742**	1.5	**3.87523E-05**	**0.00046396**
RAC-alpha/beta/gamma serine/threonine-protein kinase	1.4	**0.0220432**	0.1460222	1.1	0.3442578	0.451514
Calcium/calmodulin-dependent protein kinase type II subunit beta	1.4	**0.02237705**	0.1474851	1.4	**0.002029073**	**0.01006821**
Bcl2-associated agonist of cell death	1.4	**0.001668017**	**0.03510906**	1.7	**2.63994E-05**	**0.00034799**
Signal transducer and activator of transcription 3	1.4	0.05542197	0.2326007	1.3	**0.04573552**	0.1081247
Glutathione S-transferase P	1.4	**0.000898533**	**0.02318205**	1.3	**0.00297497**	**0.01320522**
3-hydroxyacyl-CoA dehydrogenase type-2	1.4	**0.009076646**	0.09182189	1.4	**0.005349762**	**0.02096528**
Cathepsin L2	1.4	**0.003860866**	0.05360031	1.5	**0.001129163**	**0.00627046**
High mobility group protein B1	1.4	**0.003218335**	**0.04883636**	1.2	0.1059312	0.2009305
Mothers against decapentaplegic homolog 2	1.4	**0.01287336**	0.1095303	1.1	0.3872579	0.4988861
Proteasome activator complex subunit 1	1.4	0.08122461	0.2888232	1.6	**0.000175704**	**0.00157051**
Proliferation-associated protein 2G4	1.3	0.1476629	0.3881101	1.5	**0.006335291**	**0.02422621**
Transcription factor AP-1	1.3	**0.008108964**	0.08603413	-1.0	0.9633164	0.9754775
Galectin-3	1.3	**0.001725391**	**0.03537775**	1.3	**5.98437E-07**	**2.4611E-05**
AH receptor-interacting protein	1.3	**2.24167E-05**	**0.001950251**	1.5	**3.2404E-07**	**2.1389E-05**
3-hydroxyanthranilate 3,4-dioxygenase	1.3	**0.001069789**	**0.02620595**	1.6	**9.65111E-06**	**0.00016793**
Serine/threonine-protein kinase WNK3	1.3	0.1844866	0.4314605	1.6	**0.003222071**	**0.01407188**
Peptidyl-prolyl cis-trans isomerase D	1.3	**0.02033364**	0.1382537	1.2	**0.03882553**	0.09541868
cAMP-dependent protein kinase catalytic subunit alpha	1.3	0.06662826	0.2593784	1.5	**0.002146684**	**0.01061145**
Ribosomal protein S6 kinase alpha-3	1.3	**0.02331203**	0.1507135	1.4	**0.002382557**	**0.01138915**
Heat shock protein beta-1	1.3	**0.03067081**	0.1740235	1.2	0.1157832	0.2122149
Protein kinase C iota type	1.3	**0.0188114**	0.1299289	1.4	**0.002233736**	**0.0109177**
Importin subunit beta-1	1.3	0.2488156	0.5016866	1.7	**0.004655595**	**0.0189861**
Copine-1	1.3	**0.01113808**	0.09870808	1.1	0.5996212	0.6962635
Histone deacetylase 8	1.3	**5.50876E-06**	**0.000637664**	-1.1	0.5018718	0.6106399
E3 ubiquitin-protein ligase CHIP	1.3	**0.01641444**	0.1224048	1.1	0.3454337	0.4526014
SH2 domain-containing protein 1A	1.3	**0.000186826**	**0.008964528**	1.5	**0.000188547**	**0.00166253**
Histidine triad nucleotide-binding protein 1	1.3	**0.01627995**	0.1220996	1.4	**0.000755448**	**0.00460682**
Insulin-like growth factor I	1.3	**0.002647116**	**0.04486345**	1.4	**0.000155384**	**0.0014484**
Tyrosine-protein kinase ZAP-70	1.3	**0.001909123**	**0.03718515**	-1.2	0.275385	0.385598
Cathepsin F	1.3	**0.02190946**	0.1458767	1.1	0.270493	0.3799713
Intercellular adhesion molecule 2	1.3	0.1481064	0.3881101	1.4	**0.001588809**	**0.00829358**
N-acetylglucosamine-6-sulfatase	1.3	**0.004026218**	0.05476303	1.4	**0.00022791**	**0.00184425**
14-3-3 protein epsilon	1.3	0.0799384	0.2858071	1.2	**0.04145824**	0.1001591
Glycylpeptide N-tetradecanoyltransferase 1	1.3	**0.00476061**	0.05973651	-1.0	0.9908185	0.9961755
Diablo homolog, mitochondrial	1.3	**0.02034078**	0.1382537	1.2	**0.0309178**	0.08014649
Peroxiredoxin-6	1.3	0.1612314	0.4035042	1.4	**0.008809092**	**0.03145648**
Serine protease 27	1.3	**0.04326659**	0.2107563	-1.2	0.4160175	0.5281156
Glucose-6-phosphate isomerase	1.3	0.05175879	0.2289668	1.3	**0.01587402**	**0.04932285**
Xaa-Pro aminopeptidase 1	1.3	**0.0094383**	0.09331046	1.2	0.1112822	0.2080563
Peptidyl-prolyl cis-trans isomerase A	1.3	**0.04534917**	0.2128801	1.2	**0.04971649**	0.1150355
Heat shock 70 kDa protein 1A	1.3	0.09187074	0.3066274	1.3	**0.03608819**	0.09022047
'Inosine-5"-monophosphate dehydrogenase 2'	1.3	**0.02882618**	0.1671918	1.2	0.2068259	0.3195678
Endoplasmic reticulum resident protein 29	1.3	**0.01041376**	0.09610876	1.3	**0.000199311**	**0.00174564**
'Calcium/calmodulin-dependent 3",5"-cyclic nucleotide phosphodiesterase 1A'	1.3	**0.01874857**	0.1299289	1.2	0.2262715	0.3382408
Plasma serine protease inhibitor	1.2	**0.002466217**	**0.04291217**	1.4	**5.0521E-06**	**0.00011773**
Acid sphingomyelinase-like phosphodiesterase 3a	1.2	**0.003486073**	0.05066447	1.1	0.1279844	0.2275471
Testican-2	1.2	**0.002099874**	**0.03909605**	1.1	0.07054709	0.1504313
Placenta growth factor	1.2	0.06178514	0.2473301	1.3	**0.000655539**	**0.00415655**
Non-receptor tyrosine-protein kinase TYK2	1.2	**0.006560894**	0.07386947	1.1	0.7118067	0.7852136
Dynein light chain roadblock-type 1	1.2	**0.002727098**	**0.04562644**	1.2	**0.000627806**	**0.0040359**
C-type lectin domain family 4 member M	1.2	**0.001032487**	**0.02591144**	1.2	**0.02405562**	0.06608966
WNT1-inducible-signaling pathway protein 1	1.2	**0.008246777**	0.08609635	1.4	**9.83726E-05**	**0.00098658**
Fatty acid-binding protein, heart	1.2	**0.04471772**	0.2117481	1.0	0.8767869	0.9162872
Melanoma-derived growth regulatory protein	1.2	0.05516367	0.2326007	2.0	**1.14134E-07**	**1.5389E-05**
Tyrosine-protein kinase Fer	1.2	0.06264719	0.2477412	1.2	**0.01459886**	**0.04646711**
CD177 antigen	1.2	**0.03146766**	0.1756538	2.0	**1.26902E-08**	**5.4982E-06**
Apolipoprotein D	1.2	**0.02697065**	0.159985	-1.3	0.1349585	0.2354444
Kallikrein-11	1.2	**0.03149654**	0.1756538	1.3	**0.0014606**	**0.00774831**
Protein S100-A4	1.2	0.2428764	0.4959224	1.4	**0.007078425**	**0.02662059**
Phosphatidylethanolamine-binding protein 1	1.2	**0.01604494**	0.1210326	-1.0	0.4768413	0.5903965
Calcium/calmodulin-dependent protein kinase type II subunit alpha	1.2	**0.04392775**	0.2107563	1.2	**0.02031066**	0.05838198
Tyrosine-protein phosphatase non-receptor type 1	1.2	**5.86358E-06**	**0.000637664**	1.1	0.4885419	0.5997622
Kallikrein-12	1.2	**0.002812279**	**0.04587529**	-1.1	0.608796	0.7055762
Prolactin	1.2	**0.01804681**	0.1282551	1.6	**6.6082E-05**	**0.00073082**
15-hydroxyprostaglandin dehydrogenase [NAD(+)]	1.2	**0.000589819**	**0.01853008**	1.2	**0.02845297**	0.07562348
Kremen protein 2	1.2	**0.03426453**	0.1835256	-1.0	0.7664284	0.8314123
Desmoglein-1	1.2	**0.004028545**	0.05476303	-1.1	0.2857317	0.3945819
Vacuolar protein sorting-associated protein VTA1 homolog	1.2	0.2673964	0.5287157	1.5	**0.001096803**	**0.00622317**
Cytoskeleton-associated protein 2	1.2	**0.01055419**	0.09610876	1.1	0.2774778	0.3872819
Cytokine receptor common subunit gamma	1.2	0.07774708	0.2802761	1.3	**0.002765537**	**0.01251697**
Angiogenin	1.2	**0.01429093**	0.1157688	1.4	**3.81533E-06**	**9.575E-05**
Inhibitor of growth protein 1	1.2	**0.003292517**	**0.04927867**	1.4	**0.001024686**	**0.00591688**
Regenerating islet-derived protein 4	1.2	0.0502098	0.2254552	1.6	**3.76249E-06**	**9.575E-05**
Leucine carboxyl methyltransferase 1	1.2	**2.61897E-05**	**0.002136095**	1.2	**1.55585E-05**	**0.00023204**
Glycerol-3-phosphate dehydrogenase [NAD(+)], cytoplasmic	1.2	**0.008010646**	0.08568765	-1.1	0.4814995	0.5939101
Complement factor D	1.2	**0.01369575**	0.112668	1.4	**5.78168E-07**	**2.4611E-05**
Ubiquitin-fold modifier-conjugating enzyme 1	1.2	**0.01049048**	0.09610876	1.1	0.3415743	0.4496686
Interleukin-37	1.2	**0.0144617**	0.1157688	-1.1	0.4268779	0.5382374
Seizure 6-like protein 2	1.2	0.05603874	0.2343928	1.2	**0.01005698**	**0.03435696**
Heterogeneous nuclear ribonucleoprotein Q	1.2	**0.005340381**	0.0663733	-1.2	0.2921918	0.3996963
SPARC	1.2	**0.001625874**	**0.03478304**	1.2	**0.000489444**	**0.00334411**
Polymeric immunoglobulin receptor	1.2	0.1266374	0.3554019	1.3	**0.000623876**	**0.00403048**
RAC-beta serine/threonine-protein kinase	1.2	0.06683279	0.2593784	1.3	**0.004650224**	**0.0189861**
Alpha-enolase	1.2	**0.03978228**	0.1995106	1.1	0.1956006	0.3079117
Choline/ethanolamine kinase	1.2	**0.000804869**	**0.02220511**	1.1	0.4101264	0.521652
Small ubiquitin-related modifier 3	1.2	**0.02179146**	0.1458351	-1.1	**0.01820057**	0.05435182
'Dual 3",5"-cyclic-AMP and -GMP phosphodiesterase 11A'	1.2	**0.00633521**	0.07329165	1.1	0.09244505	0.1841844
Protein FAM107A	1.2	**0.002998996**	**0.04715288**	1.0	0.7526039	0.8198231
Cathepsin H	1.2	**0.04429401**	0.2117351	1.1	0.2397746	0.3523715
Ubiquitin-conjugating enzyme E2 L3	1.2	**0.04390272**	0.2107563	1.2	**0.01269592**	**0.04162859**
Serum albumin	1.2	0.1194934	0.3506601	1.5	**9.0032E-05**	**0.00093247**
Alpha-L-iduronidase	1.2	**0.02062632**	0.1394681	-1.0	0.9631553	0.9754775
Interleukin-16	1.2	**0.002972632**	**0.04715288**	1.2	**0.001574659**	**0.00826617**
Secreted frizzled-related protein 3	1.2	**0.01863059**	0.1299289	1.2	**0.000180071**	**0.00159859**
Cytosolic non-specific dipeptidase	1.2	**0.03527971**	0.1871546	-1.1	0.6901844	0.7678522
C-C motif chemokine 15	1.2	0.1419308	0.3811015	1.5	**4.62563E-08**	**9.5034E-06**
Glypican-3	1.2	0.06705786	0.2593784	1.9	**7.30314E-06**	**0.00013812**
Protein disulfide-isomerase A3	1.1	**0.001756404**	**0.03537775**	1.1	0.2862225	0.3948418
14-3-3 protein family	1.1	0.1303061	0.3635127	-1.2	**0.01690115**	0.05117401
Matrilin-2	1.1	**0.000328669**	**0.01299734**	1.3	**6.56676E-07**	**2.5205E-05**
Elongation factor 1-beta	1.1	**0.03713617**	0.1930193	1.1	0.1489318	0.2518073
Peptidyl-prolyl cis-trans isomerase E	1.1	**0.03804636**	0.1947078	1.2	**0.001815996**	**0.00925732**
14-3-3 protein theta	1.1	0.139315	0.3798549	1.2	**0.04235657**	0.1014226
Decorin	1.1	0.2113888	0.4651979	1.4	**0.00146024**	**0.00774831**
High affinity nerve growth factor receptor	1.1	**0.02675416**	0.1594255	1.1	0.06378556	0.1391976
Neurexophilin-1	1.1	0.06501376	0.255496	1.2	**0.009437314**	**0.03292966**
Thrombopoietin	1.1	**0.01795569**	0.1282551	1.6	**0.01925186**	0.05671526
Cadherin-1	1.1	0.08738557	0.3016933	1.6	**6.06043E-07**	**2.4611E-05**
Platelet glycoprotein 4	1.1	0.1530893	0.3940463	1.3	**0.003288094**	**0.01430321**
Interleukin-10 receptor subunit alpha	1.1	**0.04903273**	0.2237332	-1.0	0.7048987	0.7802313
Casein kinase II 2-alpha:2-beta heterotetramer	1.1	0.2317255	0.4886696	1.4	**0.00102456**	**0.00591688**
Chorionic somatomammotropin hormone	1.1	**0.00142151**	**0.03144187**	1.1	**0.01494306**	**0.04721718**
Kallikrein-7	1.1	**0.02874498**	0.1671918	-1.1	0.2794129	0.3883214
Dynactin subunit 2	1.1	**0.000716092**	**0.02123865**	1.2	**0.002849144**	**0.01277709**
Galectin-9	1.1	**0.003841196**	0.05360031	1.0	0.920196	0.9448118
Transmembrane glycoprotein NMB	1.1	0.1399141	0.3798549	1.2	**0.01916838**	0.05671526
Parathyroid hormone	1.1	0.2302445	0.4869838	-1.3	**0.04884261**	0.1138207
'Inosine-5"-monophosphate dehydrogenase 1'	1.1	0.2554967	0.5113852	1.2	**0.04227069**	0.101403
Peptidyl-prolyl cis-trans isomerase F, mitochondrial	1.1	**0.002022754**	**0.03825643**	1.1	0.1034229	0.1987471
Bcl-2-related protein A1	1.1	**0.03275792**	0.179618	1.3	**0.000202489**	**0.00176166**
Tumor necrosis factor receptor superfamily member 17	1.1	0.05203977	0.2292903	1.6	**7.56283E-09**	**4.9347E-06**
Small glutamine-rich tetratricopeptide repeat-containing protein alpha	1.1	0.2937921	0.5613621	1.3	**0.003712479**	**0.01583263**
Alpha-2-HS-glycoprotein	1.1	0.1379463	0.3777948	1.5	**3.75456E-05**	**0.00045368**
Dual specificity mitogen-activated protein kinase kinase 1	1.1	0.09671268	0.3163215	1.2	**0.0222867**	0.06227869
Fibrinogen gamma chain	1.1	0.7774092	0.8993962	4.1	**0.000476288**	**0.00327134**
S-phase kinase-associated protein 1	1.1	0.07306225	0.2693396	1.2	**0.02072921**	0.05919391
Transcription factor IIIB 90 kDa subunit	1.1	**0.01068179**	0.09613607	-1.1	0.332124	0.4413665
Dermatopontin	1.1	0.198197	0.4474863	1.7	**2.10659E-08**	**5.4982E-06**
Mitogen-activated protein kinase 1	1.1	**0.04069037**	0.2019047	1.2	**0.000169834**	**0.00153912**
Ectodysplasin-A, secreted form	1.1	0.05880082	0.2405488	1.2	**9.1669E-06**	**0.00016387**
Interleukin-3 receptor subunit alpha	1.1	0.1085466	0.3348779	1.4	**0.002307509**	**0.0111118**
Annexin A6	1.1	0.1177896	0.3493531	1.4	**0.00228997**	**0.01106819**
Clusterin	1.1	0.06815775	0.2600756	1.1	**0.002816289**	**0.0126733**
EGF-containing fibulin-like extracellular matrix protein 1	1.1	**0.02606775**	0.1573649	1.1	0.05244862	0.1205025
Interleukin-17D	1.1	0.2006593	0.4522633	1.4	**0.000794084**	**0.00472005**
Triosephosphate isomerase	1.1	0.1484684	0.3882791	-1.3	**0.002023074**	**0.01006821**
Beta-2-microglobulin	1.1	**0.01515638**	0.1163475	1.1	**0.03025776**	0.07897276
C-C motif chemokine 16	1.1	0.1287391	0.3605248	1.7	**5.26247E-07**	**2.4611E-05**
Mitochondrial import inner membrane translocase subunit TIM14	1.1	**0.00623094**	0.07325565	1.2	**0.004617631**	**0.01894971**
MAP kinase-activated protein kinase 2	1.1	**0.029915**	0.1719783	1.1	0.1451725	0.2482964
C5a anaphylatoxin	1.1	0.4964117	0.7306024	1.5	**0.00853678**	**0.03077486**
Drebrin-like protein	1.1	0.07733804	0.2795738	1.2	**0.000171084**	**0.00153975**
Latent-transforming growth factor beta-binding protein 4	1.1	0.0526849	0.2292903	1.2	**0.000498336**	**0.00337077**
Cardiotrophin-1	1.1	**0.004517095**	0.05779224	1.1	**0.002167427**	**0.01067356**
Coiled-coil domain-containing protein 80	1.1	**0.01292542**	0.1095303	1.1	**0.02017653**	0.05838198
CD97 antigen	1.1	**0.004378638**	0.05723078	-1.2	0.2155457	0.3277889
Tumor necrosis factor receptor superfamily member 3	1.1	**0.02348793**	0.1508924	-1.2	0.2280172	0.3404605
Carboxypeptidase B2	1.1	0.09184355	0.3066274	1.3	**0.000767847**	**0.00463908**
Trypsin-2	1.1	0.3147636	0.5777306	1.6	**0.000217524**	**0.00180808**
Hepatocyte growth factor-like protein	1.1	0.340398	0.5994864	1.4	**0.000413964**	**0.00292013**
Insulin-like growth factor-binding protein 2	1.1	**0.006632926**	0.07386947	1.1	0.06049599	0.1342641
Dipeptidyl peptidase 2	1.1	**0.04390265**	0.2107563	-1.1	0.2341739	0.3468751
Alpha-2-antiplasmin	1.1	0.1743939	0.4183531	1.5	**8.31268E-05**	**0.00087484**
Vitamin K-dependent protein S	1.1	**0.03565747**	0.1883927	1.3	**1.60979E-05**	**0.00023342**
Kinesin-like protein KIF23	1.1	**0.009607079**	0.09354731	1.1	0.1322488	0.231037
Prostaglandin G/H synthase 2	1.1	**0.01764766**	0.1279455	1.0	0.4536448	0.5675996
Complement component C8	1.1	**0.01820282**	0.1284036	-1.2	0.2093441	0.3217834
Kunitz-type protease inhibitor 2	1.1	0.1256777	0.3535328	1.2	**0.000775331**	**0.0046627**
Arylsulfatase A	1.1	0.221972	0.4798203	1.2	**0.03095313**	0.08014649
Coagulation factor IXab	1.1	0.1234948	0.3535328	1.3	**2.08655E-05**	**0.00028072**
Glucagon	1.1	0.2890722	0.5577438	1.2	**0.004738968**	**0.01911897**
Osteomodulin	1.1	**0.0256301**	0.1573649	1.2	**2.00776E-06**	**5.6959E-05**
Tumor necrosis factor receptor superfamily member 13C	1.1	0.06221514	0.2477412	1.1	**0.04671318**	0.1094447
Collectin-11	1.1	0.3247573	0.5906069	1.2	**0.02028088**	0.05838198
Alpha-1-antitrypsin	1.1	0.3617698	0.6246006	1.6	**0.000213263**	**0.00180719**
Mitogen-activated protein kinase 8	1.1	**0.01474004**	0.1157688	1.1	0.07902578	0.1642608
Prefoldin subunit 5	1.1	0.3797748	0.6386676	1.3	**0.004999328**	**0.01995145**
Casein kinase II subunit alpha	1.1	**0.01499228**	0.1157688	1.2	**0.000660149**	**0.00416181**
Ubiquitin-conjugating enzyme E2 G2	1.1	**0.0106051**	0.09610876	-1.0	0.5034855	0.6106399
Apoptosis regulator Bcl-2	1.1	0.08722195	0.3016933	1.1	**0.01597342**	**0.04951382**
Matrilysin	1.1	0.3519306	0.6131769	1.9	**1.35826E-06**	**4.656E-05**
N-acylethanolamine-hydrolyzing acid amidase	1.1	0.3359584	0.5976597	1.3	**0.001169659**	**0.00641727**
Ribonuclease H1	1.1	0.1225381	0.3535328	1.2	**0.01427234**	**0.04598864**
Calcium/calmodulin-dependent protein kinase kinase 1	1.1	0.4475343	0.6879061	1.4	**0.001354653**	**0.00727499**
Cyclin-dependent kinase 8:Cyclin-C complex	1.1	**0.01464246**	0.1157688	-1.2	0.1794862	0.2902471
Trypsin-1	1.1	0.3794078	0.6386676	1.6	**0.000498512**	**0.00337077**
Thyroid peroxidase	1.1	**0.008706983**	0.08946939	-1.2	0.1736661	0.2825864
Cathepsin D	1.1	0.05501196	0.2326007	1.1	**0.01292471**	**0.04227254**
Lumican	1.1	0.4291152	0.6771407	1.4	**1.33711E-05**	**0.00020773**
Methionine aminopeptidase 1	1.1	**0.006017401**	0.07138826	1.0	0.1832156	0.2944536
Lithostathine-1-alpha	1.1	0.4796519	0.7170054	1.7	**2.832E-07**	**2.1389E-05**
Thioredoxin domain-containing protein 12	1.1	0.1145443	0.3442935	1.2	**6.76509E-05**	**0.00074189**
40S ribosomal protein SA	1.1	0.1973957	0.4472245	1.2	**0.003476978**	**0.01492584**
Caspase-10	1.1	0.1800882	0.4273002	1.1	**0.02828504**	0.07548462
Metalloproteinase inhibitor 2	1.1	**0.01462125**	0.1157688	1.1	**0.001285978**	**0.00696349**
Coagulation factor IX	1.1	0.1870631	0.4343726	1.3	**0.000332975**	**0.00246893**
Integrin alpha-V: beta-5 complex	1.1	0.4789882	0.7170054	1.3	**0.000298333**	**0.00225043**
Olfactomedin-4	1.1	**0.04993791**	0.2254552	1.1	0.3735538	0.484099
L-Selectin	1.1	0.1447277	0.3854483	1.2	**0.00011641**	**0.00113369**
Testican-1	1.1	0.3353693	0.5976597	-1.1	**0.02660176**	0.07157792
Microtubule-associated protein tau	1.1	**0.04808082**	0.2217154	-1.0	0.6195001	0.7116616
Neutral ceramidase	1.1	0.3276624	0.5906069	1.2	**0.001170353**	**0.00641727**
Fibroblast growth factor 7	1.1	0.2569491	0.5127196	1.6	**0.000215177**	**0.00180808**
Fibroblast growth factor 8 isoform A	1.1	0.2294772	0.486149	1.1	**0.03900748**	0.09555497
Matrilin-3	1.1	**0.0449326**	0.2117481	-1.2	0.1352089	0.2354444
Cystatin-C	1.1	**0.01498126**	0.1157688	1.0	0.3383283	0.446881
Histidine-rich glycoprotein	1.1	0.364413	0.6262711	1.4	**0.000679268**	**0.00426175**
Tyrosine-protein kinase JAK2	1.1	0.1562671	0.3967481	1.1	**0.03322199**	0.08451209
Ficolin-2	1.1	**0.03431437**	0.1835256	-1.0	0.4899752	0.6009564
Oncostatin-M	1.1	**0.02628777**	0.1573649	-1.1	0.2929617	0.4003299
Angiostatin	1.1	0.6300779	0.8197778	1.6	**1.60326E-05**	**0.00023342**
Hepatocyte growth factor activator	1.1	0.4425454	0.6850791	1.3	**0.002615692**	**0.01203345**
Hepatoma-derived growth factor-related protein 2	1.1	0.05386268	0.2316801	1.1	**0.008572442**	**0.03081828**
CD109 antigen	1.1	0.4627086	0.7021333	1.1	**0.04034181**	0.09822027
Endostatin	1.1	0.2047898	0.457621	1.3	**1.76367E-06**	**5.2309E-05**
Cytochrome c	1.1	0.6654209	0.8382502	-1.3	**0.01017754**	**0.03451705**
Advanced glycosylation end product-specific receptor, soluble	1.1	0.05710591	0.2380933	1.1	**0.006348938**	**0.02422621**
Insulin-like growth factor-binding protein 5	1.1	0.06525569	0.255496	1.1	**0.007169865**	**0.02688699**
Tenascin	1.1	0.3745671	0.6353684	-1.1	**0.0297508**	0.07811829
TATA-box-binding protein	1.1	0.2317904	0.4886696	1.2	**0.001106029**	**0.00624835**
Proteasome subunit alpha type-6	1.1	0.1706002	0.413147	1.1	**0.002718496**	**0.01240433**
Allograft inflammatory factor 1	1.1	0.6903315	0.8521464	1.2	**0.02415734**	0.06622969
Programmed cell death 1 ligand 2	1.1	0.440784	0.684981	1.2	**0.003224132**	**0.01407188**
Fibroblast growth factor 5	1.1	0.2920565	0.5596677	-1.2	**0.03253669**	0.08309271
Thymidine kinase, cytosolic	1.1	0.2782594	0.542793	1.1	**0.02036844**	0.05841937
C-C motif chemokine 28	1.1	0.5535259	0.769987	1.3	**0.003191108**	**0.0140689**
Coagulation factor Xa	1.1	0.3131737	0.5772482	1.1	**0.003340589**	**0.01448329**
Coagulation factor VII	1.0	0.4054231	0.6584679	1.4	**0.000396047**	**0.00283821**
Semaphorin-3E	1.0	0.4724826	0.7119974	-1.2	**0.002544515**	**0.01185926**
Insulin-like growth factor-binding protein 6	1.0	0.0998215	0.3204265	1.1	**0.001717674**	**0.00887239**
Eotaxin	1.0	0.5802718	0.7877817	1.3	**0.000744305**	**0.00456018**
Thyroxine-binding globulin	1.0	0.1510561	0.391905	1.1	**0.000895214**	**0.0052624**
MAP kinase-activated protein kinase 5	1.0	0.1115047	0.3384547	1.1	**0.005984805**	**0.02317558**
Cell adhesion molecule-related/down-regulated by oncogenes	1.0	0.3857641	0.642481	-1.2	**0.002771958**	**0.01251697**
C-reactive protein	1.0	0.6751014	0.8438768	1.6	**3.3101E-05**	**0.00041654**
26S proteasome non-ATPase regulatory subunit 7	1.0	0.4689861	0.7091853	1.1	**0.03418623**	0.08595959
Biglycan	1.0	0.4528002	0.6919253	1.2	**0.002411665**	**0.01148622**
Iduronate 2-sulfatase	1.0	0.753874	0.8863301	-1.4	**5.63266E-06**	**0.00012397**
Fibroblast growth factor 6	1.0	0.225503	0.4800676	1.1	**0.01338068**	**0.04365447**
Stem Cell Growth Factor-beta	1.0	0.4649581	0.7047274	1.1	**0.01630422**	0.05006404
Coagulation Factor XI	1.0	0.6057505	0.805197	1.3	**0.002578105**	**0.01193059**
Protein kinase C theta type	1.0	0.4149636	0.6669058	-1.2	**0.03409193**	0.08595959
AT-rich interactive domain-containing protein 3A	1.0	0.1866119	0.4343726	1.2	**0.01879997**	0.055886
Cytokine receptor-like factor 2	1.0	0.224817	0.4800676	1.1	**0.01441008**	**0.04620431**
Interleukin-1 receptor type 2	1.0	0.6292087	0.8195503	1.5	**5.33172E-05**	**0.00059982**
Insulin-degrading enzyme	1.0	0.4085051	0.6589607	-1.2	**0.04171529**	0.1004399
Serine/threonine-protein kinase receptor R3	1.0	0.1031264	0.325236	1.1	**0.008053136**	**0.02967517**
Stromal cell-derived factor 1	1.0	0.57981	0.7877817	1.4	**0.00195518**	**0.0098135**
Transketolase	1.0	0.6284536	0.8195503	-1.2	**0.01234562**	**0.04078744**
Dual specificity mitogen-activated protein kinase kinase 4	1.0	0.5332934	0.7597685	1.2	**0.003930951**	**0.01660159**
Vascular endothelial growth factor A	1.0	0.6928329	0.8529688	-1.1	**0.02592729**	0.07011054
Fms-related tyrosine kinase 3 ligand	1.0	0.3685982	0.6314054	-1.3	**0.03681508**	0.09151176
Interferon lambda-1	1.0	0.3832844	0.6420875	1.2	**0.02717746**	0.07286461
Hexokinase-1	1.0	0.2835226	0.5508612	-1.3	**0.01095307**	**0.03674488**
R-spondin-2	1.0	0.1622257	0.4047889	1.1	**0.03655848**	0.09122146
Fibroblast growth factor receptor 2	1.0	0.5095396	0.7380125	1.1	**0.02889586**	0.07633421
Leukocyte surface antigen CD47	1.0	0.3398077	0.5994864	1.2	**0.000208383**	**0.00177738**
Fibroblast growth factor receptor 4	1.0	0.445527	0.6864376	1.1	**0.03046296**	0.07934963
Chordin-like protein 1	1.0	0.5861984	0.7902778	1.3	**8.95076E-06**	**0.00016223**
Calreticulin	1.0	0.7370809	0.8792418	-1.3	**0.0286796**	0.07591659
D-dimer	1.0	0.9471549	0.9810619	4.6	**0.000426014**	**0.00295717**
Granulins	1.0	0.5057098	0.7358639	1.2	**0.001158863**	**0.00640812**
Formimidoyltransferase-cyclodeaminase	1.0	0.5261616	0.7547258	1.1	**0.01811515**	0.0542208
Kallikrein-8	1.0	0.7207919	0.8710393	1.2	**6.44011E-06**	**0.00012966**
Ankyrin-2	1.0	0.4070551	0.6584679	-1.1	**0.01373787**	**0.04459682**
Estrogen receptor	1.0	0.3122204	0.5763051	1.1	**0.01248331**	**0.04108847**
Complement factor H-related protein 5	1.0	0.7264653	0.8721593	1.2	**0.02227334**	0.06227869
Brain natriuretic peptide 32	1.0	0.4814313	0.7188419	-1.2	**0.0462657**	0.1087387
SLAM family member 6	1.0	0.6650494	0.8382502	-1.2	**0.01985456**	0.05796465
R-spondin-3	1.0	0.7221951	0.8710393	-1.3	**0.000418822**	**0.00293851**
Stem cell growth factor-alpha	1.0	0.6866611	0.8521464	1.1	**0.009040942**	**0.03180229**
Endothelin-converting enzyme 1	1.0	0.803492	0.9094164	1.3	**0.000565817**	**0.00370989**
Alpha-1-antichymotrypsin complex	1.0	0.6201892	0.8150523	1.2	**0.002618774**	**0.01203345**
Small nuclear ribonucleoprotein F	1.0	0.663192	0.8382502	1.2	**0.008516636**	**0.03077486**
Ectonucleoside triphosphate diphosphohydrolase 3	1.0	0.5306556	0.7581623	-1.2	**0.008917913**	**0.03162466**
Afamin	1.0	0.6925882	0.8529688	1.3	**7.02646E-05**	**0.00075781**
Lipopolysaccharide-binding protein	1.0	0.76356	0.889191	1.3	**0.004587609**	**0.0189431**
Interleukin-13 receptor subunit alpha-1	1.0	0.6189779	0.8142804	1.1	**0.01998309**	0.05808002
Interleukin-1 receptor type 1	1.0	0.8117179	0.9116109	1.3	**0.007756182**	**0.02891948**
SLIT and NTRK-like protein 1	1.0	0.7593861	0.8863301	-1.3	**0.02985136**	0.07822495
CD59 glycoprotein	1.0	0.7472319	0.8848799	-1.1	**0.009143336**	**0.03207541**
Heparin cofactor 2	1.0	0.709669	0.8639161	1.2	**0.01558219**	**0.04864774**
Peroxiredoxin-5, mitochondrial	1.0	0.3859809	0.642481	-1.1	**0.01079324**	**0.036302**
Somatotropin	1.0	0.4289796	0.6771407	-1.1	**0.006822711**	**0.02580765**
Glucocorticoid receptor	1.0	0.5662012	0.7793225	1.2	**0.0293331**	0.07722101
Immunoglobulin A	1.0	0.853976	0.9311213	1.6	**0.000250495**	**0.00196925**
Interleukin-4	1.0	0.6313231	0.8197778	1.1	**0.0342661**	0.08599474
Carbonic anhydrase 7	1.0	0.7034312	0.8595297	1.2	**0.0213741**	0.06037488
Methyl-CpG-binding domain protein 4	1.0	0.5829512	0.7877817	1.1	**0.02842869**	0.07562348
Sex hormone-binding globulin	1.0	0.8157703	0.913204	1.3	**0.004175803**	**0.01752226**
Semaphorin-6B	1.0	0.8377759	0.9241738	-1.2	**0.04965232**	0.1150355
Fibroblast growth factor 10	1.0	0.7822111	0.9018151	1.2	**0.03177505**	0.08194948
Complement C2	1.0	0.7151585	0.8665668	1.1	**0.03472307**	0.0869743
Proteasome activator complex subunit 3	1.0	0.5416474	0.7616916	-1.2	**0.000568566**	**0.00370989**
Transforming growth factor beta-2	1.0	0.868564	0.942533	1.4	**0.01748978**	0.05259024
Interleukin-5	1.0	0.6173342	0.8142313	-1.1	**0.04960951**	0.1150355
A disintegrin and metalloproteinase with thrombospondin motifs 15	1.0	0.8300523	0.9202614	1.2	**0.04632851**	0.1087387
Ubiquitin	1.0	0.6892306	0.8521464	-1.1	**0.01521876**	**0.04785658**
Myc proto-oncogene protein	1.0	0.6894324	0.8521464	-1.1	**0.04110303**	0.09970158
Tyrosine-protein phosphatase non-receptor type 2	1.0	0.964498	0.9869082	-1.1	**0.0334029**	0.08464231
Interleukin-24	1.0	0.9852678	0.9909386	1.2	**0.005574011**	**0.02177869**
Follistatin-related protein 3	1.0	0.9895838	0.9926263	1.3	**9.50825E-07**	**3.5452E-05**
Histone-lysine N-methyltransferase EHMT2	1.0	0.9871419	0.9909386	-1.2	**0.006793614**	**0.02577228**
Interleukin-7 receptor subunit alpha	-1.0	0.9735301	0.9887843	-1.2	**0.02022924**	0.05838198
Lysozyme C	-1.0	0.9869694	0.9909386	1.4	**5.11429E-07**	**2.4611E-05**
3-hydroxyisobutyrate dehydrogenase, mitochondrial	-1.0	0.937415	0.9778666	-1.1	**0.03204881**	0.08249249
Toll-like receptor 2	-1.0	0.9614115	0.9869082	1.1	**0.03662874**	0.09122235
Alanine aminotransferase 1	-1.0	0.9724086	0.9887843	-1.3	**0.000524074**	**0.00346952**
Scavenger receptor class F member 2	-1.0	0.9296433	0.9728071	-1.2	**0.03955661**	0.09666924
Fibroblast growth factor 19	-1.0	0.9581084	0.9860658	1.2	**0.04346499**	0.1035069
L-lactate dehydrogenase B chain	-1.0	0.9271501	0.9728071	-1.2	**0.0293499**	0.07722101
Aspartate aminotransferase, cytoplasmic	-1.0	0.9275355	0.9728071	-1.3	**0.001043444**	**0.00596888**
C-C motif chemokine 5	-1.0	0.9813216	0.9909386	1.6	**8.19396E-05**	**0.00086936**
Adiponectin	-1.0	0.9303167	0.9728071	1.4	**0.002507346**	**0.0117633**
Heme oxygenase 2	-1.0	0.7600026	0.8863301	1.1	**0.03149024**	0.08137576
CCAAT/enhancer-binding protein beta	-1.0	0.8965363	0.9550856	-1.4	**0.03412916**	0.08595959
Complement component C6	-1.0	0.876808	0.9486224	1.2	**0.009625028**	**0.03335183**
Disintegrin and metalloproteinase domain-containing protein 9	-1.0	0.8907221	0.953403	-1.2	**0.004694928**	**0.01902758**
Phospholipase A2	-1.0	0.8125325	0.9117411	1.1	**0.01183743**	**0.03940777**
Serine/threonine-protein kinase pim-1	-1.0	0.7365894	0.8792418	-1.2	**0.01935084**	0.05674798
Growth/differentiation factor 9	-1.0	0.8313801	0.9202614	-1.2	**0.02303029**	0.06384367
Follistatin-related protein 1	-1.0	0.7383665	0.8799711	1.2	**0.002514912**	**0.0117633**
Kallistatin	-1.0	0.8027042	0.9094164	1.1	**0.003885962**	**0.01646487**
SPARC-related modular calcium-binding protein 1	-1.0	0.8045532	0.9098284	-1.2	**0.002461567**	**0.01168125**
Neuronal growth regulator 1	-1.0	0.7984157	0.9094164	-1.2	**0.002696952**	**0.0123492**
Tumor necrosis factor receptor superfamily member 10A	-1.0	0.8896503	0.953403	-1.4	**0.008822277**	**0.03145648**
Antileukoproteinase	-1.0	0.8842891	0.953403	1.6	**3.44195E-07**	**2.1389E-05**
Carbohydrate sulfotransferase 6	-1.0	0.6497027	0.83206	1.1	**0.03712975**	0.0921185
Bcl-2-like protein 1	-1.0	0.5730485	0.7855339	-1.2	**0.01382165**	**0.04475744**
Growth/differentiation factor 11/8	-1.0	0.8474385	0.9280969	-1.1	**0.008164454**	**0.02987929**
Ephrin-A2	-1.0	0.7215269	0.8710393	-1.2	**9.15828E-05**	**0.00093457**
DnaJ homolog subfamily B member 1	-1.0	0.7403832	0.8815694	1.1	**0.006961385**	**0.02625609**
Fibroblast growth factor receptor 1	-1.0	0.656972	0.8357828	-1.1	**0.008173876**	**0.02987929**
Cadherin-6	-1.0	0.6873044	0.8521464	-1.2	**0.007928911**	**0.02931226**
Cystatin-S	-1.0	0.6682895	0.8385747	-1.1	**0.02104368**	0.05969999
Heterogeneous nuclear ribonucleoprotein K	-1.0	0.43324	0.67892	-1.1	**0.01479258**	**0.04685513**
Granulysin	-1.0	0.7860484	0.903193	1.4	**0.000405893**	**0.00287875**
'cAMP-specific 3",5"-cyclic phosphodiesterase 4D'	-1.0	0.7456823	0.8846265	-1.2	**0.00825082**	**0.02999196**
Tissue factor pathway inhibitor	-1.0	0.6873073	0.8521464	1.2	**0.00065613**	**0.00415655**
Complement C4b	-1.0	0.8328189	0.9202614	-1.2	**0.01582482**	**0.04928731**
Protein SET	-1.0	0.7544689	0.8863301	1.2	**0.002222985**	**0.010906**
Estradiol 17-beta-dehydrogenase 1	-1.0	0.8393428	0.9243116	-1.5	**0.01615215**	**0.04983109**
Complement factor B	-1.0	0.6554376	0.8352989	1.2	**9.90355E-05**	**0.00098658**
G2/mitotic-specific cyclin-B1	-1.0	0.5123259	0.7412254	-1.2	**0.008072555**	**0.02967517**
Superoxide dismutase [Mn], mitochondrial	-1.0	0.6470926	0.831257	-1.1	**0.01630438**	0.05006404
Caspase-3	-1.0	0.7574153	0.8863301	1.2	**0.03332814**	0.08461716
Apolipoprotein E	-1.0	0.4916662	0.7266414	-1.2	**0.000217375**	**0.00180808**
Quinone oxidoreductase-like protein 1	-1.0	0.6951192	0.8541719	-1.3	**0.006227106**	**0.0239716**
Interferon alpha-7	-1.0	0.2042953	0.457621	-1.1	**0.0251958**	0.06878768
Platelet receptor Gi24	-1.0	0.669253	0.8389771	-1.1	**0.04893118**	0.1138239
NKG2-D type II integral membrane protein	-1.0	0.5597121	0.774575	-1.1	**0.01454834**	**0.04641953**
Tyrosine-protein phosphatase non-receptor type substrate 1	-1.0	0.6934897	0.8529727	-1.3	**3.71564E-05**	**0.00045317**
Ephrin type-A receptor 2	-1.0	0.722172	0.8710393	1.2	**0.004746779**	**0.01911897**
Coagulation Factor X	-1.0	0.482431	0.7195114	1.1	**0.009556319**	**0.03325599**
'High affinity cGMP-specific 3",5"-cyclic phosphodiesterase 9A'	-1.0	0.3299925	0.5915387	-1.1	**0.02460575**	0.0673176
Neural cell adhesion molecule L1-like protein	-1.0	0.6236018	0.8164852	-1.4	**5.79463E-06**	**0.00012397**
Roundabout homolog 2	-1.0	0.6111552	0.8080623	-1.2	**6.95959E-05**	**0.00075686**
CD48 antigen	-1.0	0.5826615	0.7877817	1.3	**0.002939409**	**0.01309191**
Junctional adhesion molecule C	-1.0	0.6419476	0.8302691	-1.3	**0.000630914**	**0.004036**
Growth hormone receptor	-1.0	0.6457943	0.831257	1.3	**0.01681919**	0.05104429
Protein FAM107B	-1.0	0.336155	0.5976597	-1.1	**0.01855823**	0.05529336
Ectonucleoside triphosphate diphosphohydrolase 1	-1.0	0.681442	0.8494949	-1.2	**0.004974588**	**0.01991361**
Roundabout homolog 3	-1.0	0.2509961	0.5046995	-1.2	**0.002485381**	**0.01170911**
Tyrosine-protein kinase Tec	-1.0	0.751756	0.8863301	-1.3	**0.0255714**	0.06937769
Neuroligin-4, X-linked	-1.0	0.645703	0.831257	-1.3	**0.000526409**	**0.00346952**
Cation-independent mannose-6-phosphate receptor	-1.0	0.5183828	0.7466771	-1.2	**0.001408557**	**0.00753347**
Phosphoglucomutase-1	-1.0	0.2972477	0.5646409	-1.2	**0.003760459**	**0.01598501**
Netrin receptor UNC5C	-1.0	0.4864122	0.7221478	-1.3	**2.61116E-06**	**7.0991E-05**
Transforming growth factor-beta-induced protein ig-h3	-1.0	0.5046831	0.7358639	1.2	**0.01955667**	0.057223
Bone morphogenetic protein 1	-1.0	0.4318587	0.67892	1.2	**0.004679648**	**0.01902474**
Tumor necrosis factor receptor superfamily member 27	-1.0	0.6632302	0.8382502	-1.1	**0.04590439**	0.1083277
Fructose-bisphosphate aldolase A	-1.0	0.5440342	0.7632215	-1.4	**1.11547E-05**	**0.00019001**
Trefoil factor 2	-1.0	0.7232304	0.8712726	1.3	**0.000900011**	**0.00526688**
Carbonic anhydrase-related protein 10	-1.0	0.688778	0.8521464	-1.2	**0.01363867**	**0.04438521**
Thrombospondin-2	-1.0	0.4988251	0.7306024	1.2	**0.008214314**	**0.02994324**
Somatostatin-28	-1.0	0.6570982	0.8357828	-1.4	**0.000519086**	**0.00345944**
Mannose-binding protein C	-1.0	0.1952233	0.4449263	1.1	**0.04121681**	0.0997921
Interleukin-10 receptor subunit beta	-1.0	0.4025897	0.6583703	-1.2	**0.007265845**	**0.02716885**
C-C motif chemokine 14	-1.0	0.6530747	0.834638	1.6	**1.12114E-05**	**0.00019001**
Interleukin-2 receptor subunit alpha	-1.0	0.4411675	0.684981	-1.3	**0.009008611**	**0.03180229**
Sonic hedgehog protein	-1.0	0.7237237	0.8712726	2.0	**0.0002297**	**0.00184425**
NudC domain-containing protein 3	-1.0	0.4060341	0.6584679	-1.2	**0.0261036**	0.07038265
Low affinity immunoglobulin gamma Fc region receptor III-B	-1.0	0.2241612	0.4800676	-1.1	**0.02719162**	0.07286461
Epidermal growth factor receptor	-1.0	0.5013479	0.7327434	-1.3	**0.000421414**	**0.00294088**
Repulsive guidance molecule A	-1.0	0.3389816	0.5994186	-1.2	**0.000373584**	**0.00273891**
Gastrin-releasing peptide	-1.0	0.5014099	0.7327434	-1.1	**0.01453142**	**0.04641953**
Complement factor I	-1.0	0.2068849	0.4594022	1.1	**0.01016168**	**0.03451705**
Natural killer cell receptor 2B4	-1.0	0.0237034	0.1508924	1.0	0.7662492	0.8314123
Troponin I, cardiac muscle	-1.0	0.3647249	0.6262711	-1.1	**0.01643495**	0.05027223
Coactosin-like protein	-1.0	0.603558	0.8050113	-1.3	**0.01546332**	**0.04839241**
Plexin-C1	-1.0	0.5063646	0.7358639	-1.4	**0.000724832**	**0.00446182**
Beta-Ala-His dipeptidase	-1.0	0.2209896	0.4790555	-1.1	**0.005208313**	**0.02053428**
Transforming growth factor beta receptor type 3	-1.0	0.4072122	0.6584679	-1.1	**0.03860244**	0.09504942
Dickkopf-related protein 3	-1.1	0.1201668	0.3506601	-1.2	**0.000149832**	**0.00140669**
Leucine-rich repeat transmembrane protein FLRT2	-1.1	0.457597	0.6968075	-1.2	**0.008273643**	**0.02999196**
Interleukin-6 receptor subunit beta	-1.1	0.3962953	0.6509934	-1.3	**5.90686E-06**	**0.00012433**
Complement C5	-1.1	0.5261395	0.7547258	1.3	**0.004819048**	**0.01935033**
Immunoglobulin M	-1.1	0.5890985	0.7917338	1.4	**0.001031592**	**0.00593052**
Cell adhesion molecule 1	-1.1	0.6060993	0.805197	-1.3	**0.000383355**	**0.00279485**
C-type mannose receptor 2	-1.1	0.342454	0.6012966	-1.2	**0.000394392**	**0.00283821**
Wnt inhibitory factor 1	-1.1	0.5394864	0.7604613	-1.4	**0.000710443**	**0.00439397**
Bone morphogenetic protein 6	-1.1	0.2836619	0.5508612	-1.1	**0.03063568**	0.07964055
Carbonic anhydrase 4	-1.1	0.5672477	0.7793225	-1.4	**1.22162E-05**	**0.00019682**
Cryptic protein	-1.1	0.3938288	0.6489225	-1.2	**0.006758001**	**0.02571193**
Membrane frizzled-related protein	-1.1	0.350134	0.6108621	-1.2	**0.01918825**	0.05671526
Macrophage colony-stimulating factor 1 receptor	-1.1	0.1031783	0.325236	-1.2	**0.001047413**	**0.00596888**
gp41 C34 peptide, HIV	-1.1	0.506187	0.7358639	-1.2	**0.002332656**	**0.0111916**
Neuronal cell adhesion molecule	-1.1	0.1223919	0.3535328	-1.3	**0.000222062**	**0.00182259**
Metalloproteinase inhibitor 1	-1.1	0.206995	0.4594022	1.2	**0.000398002**	**0.00283821**
Limbic system-associated membrane protein	-1.1	0.3265193	0.5906069	-1.3	**1.9483E-05**	**0.00026903**
Vascular endothelial growth factor D	-1.1	0.4419571	0.684981	-1.3	**0.001843593**	**0.00932515**
BMP-binding endothelial regulator protein	-1.1	**0.03317655**	0.1796489	-1.0	0.713485	0.7864003
Cadherin-2	-1.1	0.445521	0.6864376	-1.4	**0.01517622**	**0.04783808**
Bone morphogenetic protein 10	-1.1	0.1775863	0.4229016	-1.2	**0.02403838**	0.06608966
Insulin-like growth factor-binding protein 4	-1.1	**0.04761867**	0.2211472	-1.1	**0.004314114**	**0.01798696**
Complement component C7	-1.1	0.1376882	0.3777948	-1.1	**0.04278851**	0.1022692
PolyUbiquitin K48-linked	-1.1	0.531585	0.7581623	-1.2	**0.01538681**	**0.04826872**
Receptor tyrosine-protein kinase erbB-3	-1.1	0.1928052	0.4421981	-1.4	**4.65617E-05**	**0.00053773**
Ephrin-B2	-1.1	0.2897361	0.5577438	-1.4	**1.71587E-05**	**0.00024339**
C-C motif chemokine 1	-1.1	0.2435989	0.4959385	-1.3	**0.04152188**	0.1001591
Cathepsin E	-1.1	**0.02613927**	0.1573649	1.0	0.7081678	0.782041
Glypican-5	-1.1	0.3741693	0.6353684	-1.2	**0.003206032**	**0.01407188**
Reticulon-4 receptor	-1.1	0.1410952	0.3804323	-1.4	**6.69882E-06**	**0.00013048**
Neurogenic locus notch homolog protein 1	-1.1	0.2102505	0.4642587	-1.3	**3.25554E-06**	**8.6029E-05**
Insulin-like growth factor-binding protein 3	-1.1	0.1725523	0.4154332	-1.1	0.2627342	0.3724952
Tumor necrosis factor ligand superfamily member 8	-1.1	0.3639957	0.6262711	-1.3	**0.01925276**	0.05671526
Toll-like receptor 4:Lymphocyte antigen 96 complex	-1.1	0.2334552	0.4895773	-1.3	**4.33163E-05**	**0.00050871**
Mast/stem cell growth factor receptor Kit	-1.1	0.3284131	0.5909924	-1.3	**0.001330002**	**0.00717212**
PILR alpha-associated neural protein	-1.1	0.1461769	0.3877252	-1.5	**8.47011E-06**	**0.00015791**
Fibroblast growth factor 1	-1.1	0.6740569	0.8438768	2.3	**0.02092926**	0.05950476
Hepatitis A virus cellular receptor 2	-1.1	0.2375836	0.4895773	-1.3	**3.62843E-07**	**2.1523E-05**
Leucine-rich repeat transmembrane protein FLRT1	-1.1	0.2714573	0.5351235	-1.2	**0.02007926**	0.05822984
Interleukin-17B	-1.1	0.1184957	0.3497591	1.4	**0.004557098**	**0.01887941**
Calcineurin	-1.1	0.2454305	0.4981132	-1.4	**1.56474E-05**	**0.00023204**
Protein kinase C gamma type	-1.1	0.3415536	0.6007108	-1.4	**0.000246305**	**0.00195931**
Interferon alpha/beta receptor 1	-1.1	0.2411226	0.4932052	-1.2	**0.002901019**	**0.01296517**
Thymic stromal lymphopoietin	-1.1	**0.001452408**	**0.03158988**	-1.0	0.2897397	0.3979906
Protein kinase C delta type	-1.1	0.2869456	0.55641	-1.3	**0.003419564**	**0.01477659**
Growth factor receptor-bound protein 2	-1.1	0.2137166	0.4687399	-1.4	**7.74825E-05**	**0.00082881**
Serum amyloid P-component	-1.1	0.4131375	0.6647897	1.2	**0.04187519**	0.1006393
Down syndrome cell adhesion molecule	-1.1	0.2107504	0.4645765	-1.3	**0.000104539**	**0.00102574**
Hepatocyte growth factor	-1.1	0.07899001	0.2831922	1.2	**0.01790866**	0.05372599
Ephrin-A3	-1.1	0.154523	0.3961738	-1.6	**1.39616E-06**	**4.656E-05**
Extracellular matrix protein 1	-1.1	**0.03614758**	0.1894481	-1.2	**0.00020384**	**0.00176167**
Keratin, type I cytoskeletal 18	-1.1	0.2220778	0.4798203	-1.3	**0.02304243**	0.06384367
OX-2 membrane glycoprotein	-1.1	0.1637533	0.4055959	-1.6	**1.19119E-05**	**0.00019682**
Serine protease HTRA2, mitochondrial	-1.1	0.2406554	0.4930224	-1.3	**6.40668E-05**	**0.00071459**
Tumor necrosis factor receptor superfamily member 21	-1.1	0.1001793	0.3204265	-1.4	**1.4604E-05**	**0.00022422**
Interleukin-17 receptor A	-1.1	0.2047465	0.457621	-1.3	**0.002556618**	**0.01187326**
CMRF35-like molecule 6	-1.1	**0.04842925**	0.2225358	-1.2	**0.001838448**	**0.00932515**
Hemopexin	-1.1	**0.04933776**	0.2243407	1.2	**0.04380895**	0.1039467
Ephrin-B1	-1.1	0.1074497	0.3330684	-1.3	**0.000519579**	**0.00345944**
Phospholipase A2, membrane associated	-1.1	0.3371707	0.5978367	1.2	**0.02864312**	0.07591659
Lymphocyte activation gene 3 protein	-1.1	0.295814	0.5632318	1.2	**0.009912138**	**0.03404037**
Ubiquitin+1, truncated mutation for UbB	-1.1	0.1201585	0.3506601	-1.6	**3.39773E-07**	**2.1389E-05**
Complement decay-accelerating factor	-1.1	0.08832861	0.3018031	-1.3	**0.000268212**	**0.00205892**
BH3-interacting domain death agonist	-1.1	0.1196074	0.3506601	-1.2	**0.03282314**	0.08366055
Complement component C1q receptor	-1.1	0.06738369	0.2593784	-1.2	**0.000764983**	**0.00463908**
Neurogenic locus notch homolog protein 3	-1.1	0.05110817	0.2276319	-1.2	**0.000102376**	**0.00101213**
Neuropilin-1	-1.1	0.0527691	0.2292903	-1.3	**1.70663E-05**	**0.00024339**
Growth/differentiation factor 11	-1.1	0.2245726	0.4800676	-1.3	**0.000270214**	**0.00206216**
Junctional adhesion molecule B	-1.1	0.114491	0.3442935	-1.4	**1.54625E-05**	**0.00023204**
RGM domain family member B	-1.1	0.06258492	0.2477412	-1.5	**1.76605E-08**	**5.4982E-06**
Tumor necrosis factor ligand superfamily member 14	-1.1	**0.003166683**	**0.04861791**	-1.1	0.1611005	0.2671714
Glutathione S-transferase A3	-1.1	0.1416856	0.3811105	-1.6	**0.009041109**	**0.03180229**
Leukocyte immunoglobulin-like receptor subfamily B member 2	-1.1	**0.04486317**	0.2117481	1.1	0.1777128	0.287736
Serine/threonine-protein kinase PAK 6	-1.1	0.3007833	0.5664101	-1.2	**0.02785595**	0.07449184
Epidermal growth factor	-1.1	0.1844635	0.4314605	-1.4	**0.000312781**	**0.00234586**
CD166 antigen	-1.1	0.08903115	0.3018031	-1.5	**1.95846E-05**	**0.00026903**
Malate dehydrogenase, cytoplasmic	-1.1	0.1187305	0.3497591	-1.5	**1.43193E-06**	**4.656E-05**
Ciliary neurotrophic factor receptor subunit alpha	-1.1	0.212238	0.4662804	-1.4	**0.000161984**	**0.00148865**
Tumor necrosis factor receptor superfamily member 14	-1.1	0.08784939	0.3016933	-1.2	**0.01671483**	0.05084581
Contactin-4	-1.1	0.1524908	0.3932815	-1.6	**1.73919E-06**	**5.2309E-05**
Oxidized low-density lipoprotein receptor 1	-1.1	0.14059	0.3798549	-1.4	**4.36592E-05**	**0.00050871**
Ephrin-A5	-1.1	0.07825883	0.2813437	-1.4	**5.18478E-05**	**0.00058836**
Endothelial cell-selective adhesion molecule	-1.1	0.1630067	0.4055959	-1.5	**3.9786E-06**	**9.6611E-05**
E-selectin	-1.1	0.1703873	0.413147	1.2	**0.03246689**	0.08307705
C3a anaphylatoxin des Arginine	-1.1	**0.000199212**	**0.008964528**	-1.1	**0.02183967**	0.06142408
Teratocarcinoma-derived growth factor 1	-1.1	0.1429245	0.3829907	-1.3	**0.001597766**	**0.00830711**
Low affinity immunoglobulin epsilon Fc receptor	-1.1	0.1516951	0.3920041	-1.7	**1.29646E-07**	**1.5389E-05**
BDNF/NT-3 growth factors receptor	-1.1	0.1437159	0.3843221	-1.7	**4.14487E-06**	**9.8346E-05**
Neural cell adhesion molecule L1	-1.1	0.09949054	0.3204265	-1.5	**6.45821E-06**	**0.00012966**
Layilin	-1.1	0.1614017	0.4035042	-1.6	**5.84054E-07**	**2.4611E-05**
Intercellular adhesion molecule 1	-1.1	**0.01724956**	0.127179	1.1	0.1611218	0.2671714
NT-3 growth factor receptor	-1.1	0.1467836	0.3877584	-1.5	**5.76206E-06**	**0.00012397**
SLAM family member 5	-1.1	**0.03226165**	0.1791551	-1.3	**0.000219145**	**0.00181002**
Gamma-enolase	-1.1	0.1374553	0.3777948	-1.4	**3.29612E-06**	**8.6029E-05**
Vasoactive Intestinal Peptide	-1.2	**0.04364024**	0.2107563	-1.2	0.1353129	0.2354444
Fibroblast growth factor 18	-1.2	**0.002531544**	**0.04346928**	-1.0	0.4922192	0.6027323
Apolipoprotein L1	-1.2	0.191267	0.4417759	1.3	**0.01432545**	**0.0460461**
Platelet-derived growth factor receptor alpha	-1.2	0.1039228	0.3260077	-1.5	**3.489E-05**	**0.00043363**
Delta-like protein 1	-1.2	0.06777627	0.2593784	-1.5	**1.22141E-05**	**0.00019682**
Programmed cell death 1 ligand 1	-1.2	**0.004077366**	0.05485529	-1.2	**0.02212258**	0.06208596
Carbohydrate sulfotransferase 15	-1.2	0.1679131	0.4109396	-1.2	**0.01153384**	**0.03849531**
Ephrin type-B receptor 2	-1.2	0.07163521	0.2658596	-1.5	**6.57297E-06**	**0.00012997**
Serum amyloid A-1 protein	-1.2	**0.04990567**	0.2254552	-1.1	0.2053105	0.3185853
Protein jagged-1	-1.2	0.1173847	0.3489454	-1.3	**0.001990276**	**0.00995138**
CD209 antigen	-1.2	0.06040758	0.2443495	-1.3	**0.000157753**	**0.00146006**
Acidic leucine-rich nuclear phosphoprotein 32 family member B	-1.2	0.09400987	0.3104552	-1.6	**0.000169828**	**0.00153912**
Interleukin-27 receptor subunit alpha	-1.2	0.1097688	0.3370548	-1.4	**3.61592E-05**	**0.00044517**
C-C motif chemokine 3-like 1	-1.2	**0.02875618**	0.1671918	-1.1	0.0553751	0.1254592
Basigin	-1.2	0.08883082	0.3018031	-1.5	**9.57347E-06**	**0.00016793**
Tumor necrosis factor receptor superfamily member 19L	-1.2	0.1004979	0.3206597	-1.4	**3.31958E-05**	**0.00041654**
Tyrosine-protein kinase receptor Tie-1, soluble	-1.2	0.06998431	0.2650982	-1.4	**8.77691E-06**	**0.00016132**
Interleukin-6 receptor subunit alpha	-1.2	0.06727346	0.2593784	-1.4	**0.000230355**	**0.00184425**
Tumor necrosis factor receptor superfamily member 6	-1.2	**0.02159251**	0.1452486	-1.2	**0.008907333**	**0.03162466**
Leukocyte immunoglobulin-like receptor subfamily B member 1	-1.2	**0.02459431**	0.1540972	-1.2	**0.002733943**	**0.01243134**
Amphoterin-induced protein 2	-1.2	0.1706408	0.413147	-1.5	**0.000224542**	**0.00183142**
Lymphatic vessel endothelial hyaluronic acid receptor 1	-1.2	**0.008810962**	0.08983051	-1.4	**4.02389E-07**	**2.188E-05**
Neural cell adhesion molecule 1, 120 kDa isoform	-1.2	**0.04278731**	0.2107563	-1.6	**3.37335E-07**	**2.1389E-05**
Carbonic anhydrase 6	-1.2	**0.003323006**	**0.04927867**	1.1	0.258274	0.3685621
A disintegrin and metalloproteinase with thrombospondin motifs 13	-1.2	**0.0343125**	0.1835256	1.1	0.4289481	0.5403256
Scavenger receptor class F member 1	-1.2	**0.02479016**	0.1540972	-1.4	**5.42396E-06**	**0.00012204**
Ck-beta-8-1	-1.2	**0.000462098**	**0.01615546**	-1.1	0.2418941	0.3542893
Complement C3b, inactivated	-1.2	**0.01719929**	0.127179	-1.1	0.2961796	0.4034597
Resistin	-1.2	0.06539132	0.255496	-1.3	**0.001266367**	**0.00688587**
Cathepsin S	-1.2	0.07086433	0.2653279	1.2	**0.01249971**	**0.04108847**
Low-density lipoprotein receptor-related protein 8	-1.2	**0.04898063**	0.2237332	-1.7	**6.92269E-08**	**1.1293E-05**
Cystatin-F	-1.2	**0.007807015**	0.08419963	-1.0	0.2412925	0.3538053
Growth/differentiation factor 15	-1.2	**0.002988645**	**0.04715288**	1.2	**0.000358554**	**0.00264358**
Low affinity immunoglobulin gamma Fc region receptor II-a	-1.2	**0.01904987**	0.1308425	-1.2	**0.001577223**	**0.00826617**
Lactadherin	-1.2	0.3257451	0.5906069	-1.5	**9.29157E-05**	**0.00093996**
Tyrosine-protein kinase receptor TYRO3	-1.2	0.08419627	0.2961621	-1.5	**1.2125E-05**	**0.00019682**
Ephrin type-A receptor 10	-1.2	0.09405412	0.3104552	-1.9	**1.62878E-06**	**5.0609E-05**
Interleukin-2	-1.2	**0.03771351**	0.1937643	-1.2	**0.004601504**	**0.0189431**
Kin of IRRE-like protein 3	-1.2	**0.04372713**	0.2107563	-1.5	**0.00111714**	**0.00625196**
Alpha-2-macroglobulin	-1.2	**0.002407934**	**0.04246425**	-1.3	**0.005082813**	**0.02016131**
Intercellular adhesion molecule 5	-1.2	**0.04004014**	0.1995106	-2.0	**1.90531E-07**	**2.072E-05**
Fractalkine	-1.2	0.1147645	0.3442935	-1.1	**0.006162239**	**0.02379208**
Platelet glycoprotein VI	-1.2	**0.03148431**	0.1756538	1.1	0.1134589	0.2105156
Scavenger receptor cysteine-rich type 1 protein M130	-1.2	**0.006679385**	0.07386947	-1.3	**0.000683871**	**0.00427011**
Urokinase plasminogen activator surface receptor	-1.2	**0.0148204**	0.1157688	-1.5	**1.46282E-06**	**4.656E-05**
Bone morphogenetic protein receptor type-2	-1.2	**0.04343506**	0.2107563	-1.5	**0.000695938**	**0.00432476**
Interleukin-11	-1.2	**0.01006697**	0.09558431	1.0	0.5468187	0.651688
Ephrin type-A receptor 5	-1.3	**0.03936932**	0.1995106	-1.9	**6.22349E-07**	**2.4611E-05**
Insulin-like growth factor-binding protein 1	-1.3	**0.01010777**	0.09558431	1.1	0.5833102	0.6854319
Fibrinogen	-1.3	0.5741308	0.7861917	2.8	**0.001741208**	**0.00894597**
Vascular endothelial growth factor A, isoform 121	-1.3	**0.009326369**	0.09290772	-1.2	**3.11345E-05**	**0.00039834**
Syntaxin-1A	-1.3	**0.03240654**	0.1791972	-1.5	**0.000980896**	**0.0057146**
Contactin-5	-1.3	**0.04801626**	0.2217154	-1.6	**0.001225188**	**0.00668984**
Tumor necrosis factor receptor superfamily member 1B	-1.3	**1.91403E-06**	**0.000416302**	-1.2	**0.000132915**	**0.00126842**
Secreted and transmembrane protein 1	-1.3	**0.006609171**	0.07386947	-1.0	0.6718902	0.7532789
CD5 antigen-like	-1.3	**0.03501802**	0.1865245	1.2	0.07904659	0.1642608
Inhibin beta A chain:Inhibin beta B chain heterodimer	-1.3	**0.01881729**	0.1299289	1.0	0.9040789	0.934411
GDNF family receptor alpha-2	-1.3	**0.0303249**	0.1728122	-1.9	**3.0802E-07**	**2.1389E-05**
Vascular cell adhesion protein 1	-1.3	**0.001084384**	**0.02620595**	-1.0	0.3547817	0.4629901
C-C motif chemokine 23	-1.3	**0.005472239**	0.06737049	1.2	0.100294	0.1944779
Tumor necrosis factor ligand superfamily member 15	-1.3	**0.000162151**	**0.00846428**	1.0	0.7707287	0.8342643
Netrin receptor UNC5D	-1.3	**0.02460597**	0.1540972	-2.3	**6.66045E-09**	**4.9347E-06**
Ficolin-3	-1.3	**0.03824361**	0.1949528	1.2	0.1711674	0.2802678
Elafin	-1.3	0.0578717	0.2397542	1.3	**2.07546E-05**	**0.00028072**
Interleukin-18-binding protein	-1.3	**0.001138014**	**0.02651979**	-1.3	**0.000262587**	**0.00203974**
SLIT and NTRK-like protein 5	-1.3	**0.02290965**	0.1494855	-1.9	**4.41579E-07**	**2.305E-05**
Immunoglobulin superfamily containing leucine-rich repeat protein 2	-1.3	**0.02479726**	0.1540972	-2.2	**2.49234E-07**	**2.1389E-05**
Platelet factor 4	-1.4	0.08775205	0.3016933	1.6	**0.000573392**	**0.00372277**
ICOS ligand	-1.4	**0.000905965**	**0.02318205**	-1.5	**1.87269E-06**	**5.4308E-05**
Hemojuvelin	-1.4	**0.01332732**	0.1122074	-2.0	**5.99913E-07**	**2.4611E-05**
Neutrophil-activating peptide 2	-1.4	0.2354816	0.4895773	2.2	**1.33626E-05**	**0.00020773**
Connective tissue-activating peptide III	-1.4	0.2372106	0.4895773	2.0	**2.19388E-05**	**0.00029214**
Tissue-type plasminogen activator	-1.4	**0.000125724**	**0.007457736**	1.1	0.5070802	0.6135089
Ephrin type-B receptor 6	-1.4	**0.01455854**	0.1157688	-2.2	**2.47599E-07**	**2.1389E-05**
Complement C3d fragment	-1.4	**0.0043855**	0.05723078	-1.1	0.5952	0.6941341
Metalloproteinase inhibitor 3	-1.4	**0.03316236**	0.1796489	-1.6	**5.09763E-08**	**9.5034E-06**
Stromelysin-2	-1.4	**5.82691E-07**	**0.000190103**	-1.1	**0.02397563**	0.06608966
Lactotransferrin	-1.5	**0.04573701**	0.2139312	-1.2	0.3426533	0.4498617
Neurexin-1-beta	-1.5	**0.01190935**	0.1029252	-2.3	**3.14495E-07**	**2.1389E-05**
Histone H1.2	-1.5	0.1238768	0.3535328	-1.6	**0.01027816**	**0.03465892**
Protein S100-A9	-1.5	**0.000149471**	**0.00846428**	-1.3	**0.03801896**	0.09414562
Peptidoglycan recognition protein 1	-1.5	**0.000473273**	**0.01615546**	-1.0	0.6946357	0.771489
C-C motif chemokine 19	-1.6	**0.004176945**	0.05562157	-1.2	0.06985815	0.1494506
Neuroblastoma suppressor of tumorigenicity 1	-1.6	**0.003709797**	0.05262266	-2.5	**3.82831E-07**	**2.1721E-05**
Interstitial collagenase	-1.6	**2.52413E-06**	**0.000454754**	-1.2	0.2028137	0.3165606
Neutrophil gelatinase-associated lipocalin	-1.6	**0.003052739**	**0.04742648**	1.0	0.9527663	0.9691037
Complement C1r subcomponent	-1.6	0.1558698	0.3967481	-2.6	**0.01465771**	**0.04654091**
Cathepsin G	-1.6	**0.01190246**	0.1029252	-1.3	0.1318517	0.2307908
C-X-C motif chemokine 13	-1.7	**0.0397383**	0.1995106	-1.3	**0.04777114**	0.111523
Stanniocalcin-1	-1.7	**9.52099E-05**	**0.006212448**	1.1	0.4692365	0.5843069
Interleukin-1 receptor antagonist protein	-1.8	**0.01023405**	0.09608225	-1.2	0.2026755	0.3165606
Endothelial cell-specific molecule 1	-1.8	**4.56486E-05**	**0.003309524**	-1.2	0.06146679	0.1354969
Macrophage colony-stimulating factor 1	-1.8	**0.005990576**	0.07138826	-1.2	0.07157245	0.1516267
Neutrophil collagenase	-1.9	**0.00176211**	**0.03537775**	-1.8	**0.00247993**	**0.01170911**
C-C motif chemokine 22	-2.0	**0.001210459**	**0.02771313**	-1.2	0.2136068	0.326356
C3a anaphylatoxin	-2.0	**0.007488924**	0.08144205	-1.6	0.06589259	0.1428403
Tumor necrosis factor-inducible gene 6 protein	-2.0	**0.000901867**	**0.02318205**	-1.1	0.2490737	0.3611569
Hepcidin	-2.1	**1.57337E-07**	**0.000102662**	-1.0	0.9990991	0.9999265
Activated Protein C	-2.4	**0.000655014**	**0.01987892**	-3.2	**9.16668E-05**	**0.00093457**
Protein S100-A12	-2.5	**3.95704E-05**	**0.003037613**	-2.3	**0.000390664**	**0.00283231**
C-C motif chemokine 4-like	-2.8	**0.000113743**	**0.00706831**	-1.5	0.08135641	0.1677253
Plasminogen activator inhibitor 1	-2.8	**5.64918E-05**	**0.003880093**	-1.4	**0.04369913**	0.103875
C-C motif chemokine 2	-3.0	**0.000318576**	**0.01299193**	-1.0	0.8802179	0.9186173
Matrix metalloproteinase-9	-3.4	**2.78776E-06**	**0.000454754**	-2.7	**0.000132425**	**0.00126842**
Bactericidal permeability-increasing protein	-3.8	**0.000273718**	**0.01190674**	-2.6	**0.003946388**	**0.01661302**
Myeloblastin	-3.8	**1.20749E-05**	**0.001125549**	-2.6	**0.004340137**	**0.01803783**
C-C motif chemokine 7	-4.1	**0.004499379**	0.05779224	-1.4	0.05275771	0.1207874
C-X-C motif chemokine 6	-4.3	**0.000184498**	**0.008964528**	-1.5	0.1112449	0.2080563
C-X-C motif chemokine 10	-4.3	**0.01589382**	0.1205897	1.3	0.07129948	0.151294
Interleukin-1 receptor-like 1	-5.1	**4.78343E-07**	**0.000190103**	-2.3	**0.000207118**	**0.00177738**
Neutrophil elastase	-5.6	**0.000195828**	**0.008964528**	-2.9	**0.01145663**	**0.03833566**
C-X-C motif chemokine 11	-6.1	**0.01263921**	0.1085142	-1.1	0.8049845	0.8589572
Growth-regulated alpha protein	-6.8	**4.26718E-06**	**0.000556867**	-2.8	**0.000147502**	**0.00139485**
Myeloperoxidase	-6.9	**8.94952E-06**	**0.000898394**	-4.3	**0.000795717**	**0.00472005**
Interleukin-6	-9.0	**6.38441E-08**	**8.33165E-05**	-8.0	**6.14731E-06**	**0.00012734**
Interleukin-8	-11.4	**1.35757E-06**	**0.000354326**	-2.8	**5.17091E-05**	**0.00058836**

Supplementary Figure 16. CSF proteins with significant fold change following control night and acute sleep deprivation, side by side comparison. Proteins are organized by descending value of fold change in control samples. Tables are a continuation from left to right. Bolded in red are the significant values p<0.05. Of note some of the protein fold changes did not have a significant adjusted p-value. SlpD=sleep deprived. T24=Hour 24, T0= Hour 0.

Protein	Control T24 vs T0 Fold Change	Control T24 vs T0 p-value	Control T24 vs T0 adj. p-value	SlpD T24 vs T0 Fold Change	SlpD T24 vs T0 p-value	SlpD T24 vs T0 adj. p-value
Protein-glutamine gamma-glutamyltransferase E	2.847963	**0.02584031**	0.1573649	1.431121	0.2814911	0.3907935
Pulmonary surfactant-associated protein D	2.344185	**0.009180347**	0.09215656	2.618119	**0.01018319**	**0.03451705**
Creatine kinase M-type:Creatine kinase B-type heterodimer	2.186322	**0.01157484**	0.101377	1.922789	**0.008683787**	**0.03113281**
Integrin alpha-I: beta-1 complex	2.143920	**0.02559865**	0.1573649	2.164108	0.05810809	0.1296257
ATP synthase subunit O, mitochondrial	2.003917	**0.01759954**	0.1279455	1.789587	**0.04621486**	0.1087387
Phosphoglycerate mutase 1	1.976495	**0.008176638**	0.08605252	1.197363	0.468224	0.5836029
Heterogeneous nuclear ribonucleoprotein A/B	1.843170	**0.01738077**	0.1274264	1.949881	**0.02342029**	0.06475314
Heterogeneous nuclear ribonucleoproteins A2/B1	1.831196	**0.02359107**	0.1508924	2.149287	**0.02054609**	0.05879965
Pyridoxal kinase	1.805475	**0.0022183**	**0.04020669**	1.668965	**0.005613548**	**0.0218677**
Mitogen-activated protein kinase 3	1.779191	**0.006002103**	0.07138826	1.714734	**0.002259928**	**0.0110045**
Pyruvate kinase PKM	1.776453	**0.0107818**	0.09637157	1.998396	**0.000879555**	**0.00519375**
Tyrosine-protein kinase CSK	1.724273	**0.0135307**	0.1124686	1.733274	**0.007821048**	**0.02907826**
Fibroblast growth factor 2	1.715514	**0.02582264**	0.1573649	1.518908	**0.03841014**	0.0947547
Aflatoxin B1 aldehyde reductase member 2	1.672653	**0.015618**	0.11919	1.974716	**0.000786741**	**0.00470962**
GTP-binding nuclear protein Ran	1.638994	**0.0238462**	0.1510645	2.078548	**0.000268115**	**0.00205892**
Mothers against decapentaplegic homolog 3	1.625610	**0.00081674**	**0.02220511**	1.010430	0.9635176	0.9754775
Inorganic pyrophosphatase	1.623903	**0.02911335**	0.1681103	1.518742	**0.009634974**	**0.03335183**
Protein kinase C alpha type	1.620269	**0.01352033**	0.1124686	1.449668	**0.03000819**	0.07847832
Creatine kinase M-type	1.616519	**0.00963115**	0.09354731	1.268184	0.215607	0.3277889
Calcium/calmodulin-dependent protein kinase type II subunit delta	1.598948	**0.04731421**	0.220518	1.399833	**0.01024188**	**0.03462606**
Non-histone chromosomal protein HMG-14	1.597668	**0.007009917**	0.07687346	1.409083	**0.009181525**	**0.03212303**
SUMO-conjugating enzyme UBC9	1.596714	**0.006402489**	0.07329165	1.551006	**0.000510206**	**0.00343206**
N-acetyl-D-glucosamine kinase	1.586117	**0.009677308**	0.09354731	1.872845	**0.001121042**	**0.00625196**
X-ray repair cross-complementing protein 6	1.573302	**0.000284048**	**0.0119575**	1.385920	**0.003457303**	**0.01489036**
Tumor necrosis factor ligand superfamily member 12	1.560128	**0.01119448**	0.09870808	-1.015250	0.8836709	0.9196097
Alcohol dehydrogenase [NADP(+)]	1.556340	**0.008697287**	0.08946939	1.403217	**0.002274181**	**0.01103274**
Peroxiredoxin-1	1.528937	**0.0138137**	0.112668	1.552833	**0.005034345**	**0.02002994**
Cyclin-dependent kinase inhibitor 1B	1.525311	**3.48143E-06**	**0.000504807**	1.476403	**3.95177E-05**	**0.00046882**
Ras-related C3 botulinum toxin substrate 1	1.520147	**0.03609412**	0.1894481	1.280676	**0.03348133**	0.08467663
Eukaryotic translation initiation factor 4H	1.506724	**0.01373826**	0.112668	1.706500	**0.001493676**	**0.00789169**
NAD-dependent protein deacetylase sirtuin-2	1.505924	**0.001834002**	**0.03626322**	1.162565	0.2218054	0.3342448
Ribosome maturation protein SBDS	1.499978	**0.00059637**	**0.01853008**	1.541064	**1.82699E-05**	**0.00025637**
Phosphoglycerate kinase 1	1.499574	**0.000482807**	**0.01615546**	1.553304	**0.000247729**	**0.00195931**
Nucleoside diphosphate kinase B	1.479502	**0.02740358**	0.1618175	1.496598	**0.005840032**	**0.02268227**
Tropomyosin alpha-4 chain	1.463739	0.1247793	0.3535328	1.746552	**0.01658962**	0.05058284
Sorting nexin-4	1.462301	**0.009843724**	0.09445633	1.337360	**0.01201069**	**0.03988284**
40S ribosomal protein S7	1.458542	**0.01045614**	0.09610876	1.387462	**0.001720087**	**0.00887239**
Signal transducer and activator of transcription 6	1.454858	**0.03770245**	0.1937643	1.304519	0.05324931	0.1216994
ATP-dependent RNA helicase DDX19B	1.451227	**0.002405204**	**0.04246425**	1.264521	**0.03239029**	0.08304387
Eukaryotic translation initiation factor 5A-1	1.451212	0.06038873	0.2443495	1.464241	**0.006342163**	**0.02422621**
40S ribosomal protein S3a	1.450243	**0.03727269**	0.1930193	1.137718	0.2060831	0.3190255
Adenylate kinase isoenzyme 1	1.450172	0.08462361	0.2968651	1.427951	**0.001813991**	**0.00925732**
Aminoacylase-1	1.433809	**0.0005213**	**0.01700742**	1.545311	**3.87523E-05**	**0.00046396**
Bcl2-associated agonist of cell death	1.401543	**0.001668017**	**0.03510906**	1.676330	**2.63994E-05**	**0.00034799**
Cathepsin L2	1.370782	**0.003860866**	0.05360031	1.491658	**0.001129163**	**0.00627046**
Proteasome activator complex subunit 1	1.356307	0.08122461	0.2888232	1.625039	**0.000175704**	**0.00157051**
Proliferation-associated protein 2G4	1.336267	0.1476629	0.3881101	1.481324	**0.006335291**	**0.02422621**
AH receptor-interacting protein	1.332666	**2.24167E-05**	**0.001950251**	1.516267	**3.2404E-07**	**2.1389E-05**
3-hydroxyanthranilate 3,4-dioxygenase	1.328229	**0.001069789**	**0.02620595**	1.558635	**9.65111E-06**	**0.00016793**
Serine/threonine-protein kinase WNK3	1.325814	0.1844866	0.4314605	1.618687	**0.003222071**	**0.01407188**
cAMP-dependent protein kinase catalytic subunit alpha	1.318661	0.06662826	0.2593784	1.544743	**0.002146684**	**0.01061145**
Importin subunit beta-1	1.307448	0.2488156	0.5016866	1.690970	**0.004655595**	**0.0189861**
SH2 domain-containing protein 1A	1.299049	**0.000186826**	**0.008964528**	1.454296	**0.000188547**	**0.00166253**
Melanoma-derived growth regulatory protein	1.236286	0.05516367	0.2326007	2.034388	**1.14134E-07**	**1.5389E-05**
CD177 antigen	1.233430	**0.03146766**	0.1756538	2.006553	**1.26902E-08**	**5.4982E-06**
Prolactin	1.218659	**0.01804681**	0.1282551	1.597168	**6.6082E-05**	**0.00073082**
Protein NOV homolog	1.200327	**0.004691939**	0.05944641	1.663953	**1.29714E-07**	**1.5389E-05**
Vacuolar protein sorting-associated protein VTA1 homolog	1.196559	0.2673964	0.5287157	1.547681	**0.001096803**	**0.00622317**
Regenerating islet-derived protein 4	1.184702	0.0502098	0.2254552	1.572970	**3.76249E-06**	**9.575E-05**
Serum albumin	1.167601	0.1194934	0.3506601	1.527672	**9.0032E-05**	**0.00093247**
C-C motif chemokine 15	1.152623	0.1419308	0.3811105	1.527827	**4.62563E-08**	**9.5034E-06**
Glypican-3	1.150790	0.06705786	0.2593784	1.863807	**7.30314E-06**	**0.00013812**
Thrombopoietin	1.143517	**0.01795569**	0.1282551	1.618087	**0.01925186**	0.05671526
Cadherin-1	1.142319	0.08738557	0.3016933	1.562415	**6.06043E-07**	**2.4611E-05**
Tumor necrosis factor receptor superfamily member 17	1.128883	0.05203977	0.2292903	1.577107	**7.56283E-09**	**4.9347E-06**
Alpha-2-HS-glycoprotein	1.123228	0.1379463	0.3777948	1.478507	**3.75456E-05**	**0.00045368**
Fibrinogen gamma chain	1.119993	0.7774092	0.8993962	4.091564	**0.000476288**	**0.00327134**
Dermatopontin	1.116984	0.198197	0.4474863	1.685898	**2.10659E-08**	**5.4982E-06**
C-C motif chemokine 16	1.105631	0.1287391	0.3605248	1.652432	**5.26247E-07**	**2.4611E-05**
C5a anaphylatoxin	1.104545	0.4964117	0.7306024	1.471378	**0.00853678**	**0.03077486**
Trypsin-2	1.100445	0.3147636	0.5777306	1.616767	**0.000217524**	**0.00180808**
Alpha-2-antiplasmin	1.099130	0.1743939	0.4183531	1.468639	**8.31268E-05**	**0.00087484**
Alpha-1-antitrypsin	1.092274	0.3617698	0.6246006	1.591531	**0.000213263**	**0.00180719**
Matrilysin	1.086773	0.3519306	0.6131769	1.918662	**1.35826E-06**	**4.656E-05**
Trypsin-1	1.085692	0.3794078	0.6386676	1.610519	**0.000498512**	**0.00337077**
Cystatin-SA	1.083261	0.3402548	0.5994864	1.483116	**0.02594896**	0.07011054
Lithostathine-1-alpha	1.070834	0.4796519	0.7170054	1.660651	**2.832E-07**	**2.1389E-05**
Fibroblast growth factor 7	1.065179	0.2569491	0.5127196	1.590499	**0.000215177**	**0.00180808**
Angiostatin	1.061879	0.6300779	0.8197778	1.586988	**1.60326E-05**	**0.00023342**
C-reactive protein	1.044251	0.6751014	0.8438768	1.577503	**3.3101E-05**	**0.00041654**
Interleukin-1 receptor type 2	1.035952	0.6292087	0.8195503	1.467616	**5.33172E-05**	**0.00059982**
D-dimer	1.027896	0.9471549	0.9810619	4.635581	**0.000426014**	**0.00295717**
Immunoglobulin A	1.018024	0.853976	0.9311213	1.561147	**0.000250495**	**0.00196925**
Interleukin-22 receptor subunit alpha-1	1.003333	0.9751459	0.9887843	-1.675151	**0.02254726**	0.06273812
C-C motif chemokine 5	-1.007219	0.9813216	0.9909386	1.589510	**8.19396E-05**	**0.00086936**
Antileukoproteinase	-1.014045	0.8842891	0.953403	1.558895	**3.44195E-07**	**2.1389E-05**
Estradiol 17-beta-dehydrogenase 1	-1.022678	0.8393428	0.9243116	-1.469130	**0.01615215**	**0.04983109**
C-C motif chemokine 14	-1.043504	0.6530747	0.834638	1.583711	**1.12114E-05**	**0.00019001**
Sonic hedgehog protein	-1.043747	0.7237237	0.8712726	2.007332	**0.0002297**	**0.00184425**
Interferon gamma	-1.071474	0.4499975	0.6900667	-1.465507	**1.12036E-06**	**4.0613E-05**
PILR alpha-associated neural protein	-1.092025	0.1461769	0.3877252	-1.535675	**8.47011E-06**	**0.00015791**
Fibroblast growth factor 1	-1.093060	0.6740569	0.8438768	2.304650	**0.02092926**	0.05950476
Ephrin-A3	-1.110217	0.154523	0.3961738	-1.602100	**1.39616E-06**	**4.656E-05**
OX-2 membrane glycoprotein	-1.111801	0.1637533	0.4055959	-1.588239	**1.19119E-05**	**0.00019682**
Ubiquitin+1, truncated mutation for UbB	-1.117503	0.1201585	0.3506601	-1.636731	**3.39773E-07**	**2.1389E-05**
RGM domain family member B	-1.125503	0.06258492	0.2477412	-1.494987	**1.76605E-08**	**5.4982E-06**
Glutathione S-transferase A3	-1.127397	0.1416856	0.3811105	-1.631930	**0.009041109**	**0.03180229**
CD166 antigen	-1.129408	0.08903115	0.3018031	-1.530874	**1.95846E-05**	**0.00026903**
Malate dehydrogenase, cytoplasmic	-1.130299	0.1187305	0.3497591	-1.471102	**1.43193E-06**	**4.656E-05**
Contactin-4	-1.131209	0.1524908	0.3932815	-1.586403	**1.73919E-06**	**5.2309E-05**

Protein	Control T24 vs T0 Fold Change	Control T24 vs T0 p-value	Control T24 vs T0 adj. p-value	SlpD T24 vs T0 Fold Change	SlpD T24 vs T0 p-value	SlpD T24 vs T0 adj. p-value
Endothelial cell-selective adhesion molecule	-1.132182	0.1630067	0.4055959	-1.520300	**3.9786E-06**	**9.6611E-05**
Low affinity immunoglobulin epsilon Fc receptor	-1.136813	0.1516951	0.3920041	-1.655725	**1.29646E-07**	**1.5389E-05**
BDNF/NT-3 growth factors receptor	-1.140420	0.1437159	0.3843221	-1.660820	**4.14487E-06**	**9.8346E-05**
Neural cell adhesion molecule L1	-1.142277	0.09949054	0.3204265	-1.528831	**6.45821E-06**	**0.00012966**
Layilin	-1.143247	0.1614017	0.4035042	-1.594460	**5.84054E-07**	**2.4611E-05**
NT-3 growth factor receptor	-1.144614	0.1467836	0.3877584	-1.547391	**5.76206E-06**	**0.00012397**
Platelet-derived growth factor receptor alpha	-1.153614	0.1039228	0.3260077	-1.487146	**3.489E-05**	**0.00043363**
Delta-like protein 1	-1.155159	0.06777627	0.2593784	-1.474977	**1.22141E-05**	**0.00019682**
Ephrin type-B receptor 2	-1.157439	0.07163521	0.2658596	-1.528541	**6.57297E-06**	**0.00012997**
Acidic leucine-rich nuclear phosphoprotein 32 family member B	-1.162322	0.09400987	0.3104552	-1.645387	**0.000169828**	**0.00153912**
Basigin	-1.164975	0.08883082	0.3018031	-1.541431	**9.57347E-06**	**0.00016793**
Amphoterin-induced protein 2	-1.177851	0.1706408	0.413147	-1.544206	**0.000224542**	**0.00183142**
Neural cell adhesion molecule 1, 120 kDa isoform	-1.180829	**0.04278731**	0.2107563	-1.591679	**3.37335E-07**	**2.1389E-05**
Low-density lipoprotein receptor-related protein 8	-1.191057	**0.04898063**	0.2237332	-1.670986	**6.92269E-08**	**1.1293E-05**
Lactadherin	-1.197085	0.3257451	0.5906069	-1.496522	**9.29157E-05**	**0.00093996**
Tyrosine-protein kinase receptor TYRO3	-1.197821	0.08419627	0.2961621	-1.542172	**1.2125E-05**	**0.00019682**
Chromogranin-A	-1.211882	0.1606085	0.4035042	-1.724173	**5.28923E-06**	**0.0001211**
Hepatocyte growth factor receptor	-1.213802	**0.03291072**	0.1796489	-1.564093	**2.44603E-06**	**6.7916E-05**
Ephrin-B3	-1.225108	0.07457752	0.2726153	-1.767751	**1.26079E-06**	**4.4469E-05**
Ephrin type-A receptor 10	-1.227587	0.09405412	0.3104552	-1.861275	**1.62878E-06**	**5.0609E-05**
Kin of IRRE-like protein 3	-1.234326	**0.04372713**	0.2107563	-1.451620	**0.00111714**	**0.00625196**
Intercellular adhesion molecule 5	-1.238097	**0.04004014**	0.1995106	-1.985274	**1.90531E-07**	**2.072E-05**
Urokinase plasminogen activator surface receptor	-1.244578	**0.0148204**	0.1157688	-1.529156	**1.46282E-06**	**4.656E-05**
Bone morphogenetic protein receptor type-2	-1.244831	**0.04343506**	0.2107563	-1.508688	**0.000695938**	**0.00432476**
Ephrin type-A receptor 5	-1.252602	**0.03936932**	0.1995106	-1.874977	**6.22349E-07**	**2.4611E-05**
Fibrinogen	-1.256622	0.5741308	0.7861917	2.812167	**0.001741208**	**0.00894597**
Syntaxin-1A	-1.267543	**0.03240654**	0.1791972	-1.481446	**0.000980896**	**0.0057146**
Contactin-5	-1.282266	**0.04801626**	0.2217154	-1.558823	**0.001225188**	**0.00668984**
GDNF family receptor alpha-2	-1.302479	**0.0303249**	0.1728122	-1.926497	**3.0802E-07**	**2.1389E-05**
Netrin receptor UNC5D	-1.320899	**0.02460597**	0.1540972	-2.310046	**6.66045E-09**	**4.9347E-06**
SLIT and NTRK-like protein 5	-1.333705	**0.02290965**	0.1494855	-1.910978	**4.41579E-07**	**2.305E-05**
Immunoglobulin superfamily containing leucine-rich repeat protein 2	-1.343433	**0.02479726**	0.1540972	-2.203472	**2.49234E-07**	**2.1389E-05**
Platelet factor 4	-1.366305	0.08775205	0.3016933	1.602803	**0.000573392**	**0.00372277**
ICOS ligand	-1.369804	**0.000905965**	**0.02318205**	-1.542713	**1.87269E-06**	**5.4308E-05**
Hemojuvelin	-1.370969	**0.01332732**	0.1122074	-1.982324	**5.99913E-07**	**2.4611E-05**
Neutrophil-activating peptide 2	-1.372095	0.2354816	0.4895773	2.201515	**1.33626E-05**	**0.00020773**
Connective tissue-activating peptide III	-1.382915	0.2372106	0.4895773	2.008190	**2.19388E-05**	**0.00029214**
Ephrin type-B receptor 6	-1.389829	**0.01455854**	0.1157688	-2.193880	**2.47599E-07**	**2.1389E-05**
Metalloproteinase inhibitor 3	-1.398544	**0.03316236**	0.1796489	-1.631293	**5.09763E-08**	**9.5034E-06**
Lactotransferrin	-1.455677	**0.04573701**	0.2139312	-1.156866	0.3426533	0.4498617
Neurexin-1-beta	-1.460018	**0.01190935**	0.1029252	-2.346457	**3.14495E-07**	**2.1389E-05**
Histone H1.2	-1.460732	0.1238768	0.3535328	-1.638497	**0.01027816**	**0.03465892**
Protein S100-A9	-1.491034	**0.000149471**	**0.00846428**	-1.307918	**0.03801896**	0.09414562
Peptidoglycan recognition protein 1	-1.527423	**0.000473273**	**0.01615546**	-1.048025	0.6946357	0.771489
C-C motif chemokine 19	-1.552661	**0.004176945**	0.05562157	-1.238743	0.06985815	0.1494506
Neuroblastoma suppressor of tumorigenicity 1	-1.553102	**0.003709797**	0.05262266	-2.475061	**3.82831E-07**	**2.1721E-05**
Interstitial collagenase	-1.562648	**2.52413E-06**	**0.000454754**	-1.180653	0.2028137	0.3165606
Neutrophil gelatinase-associated lipocalin	-1.567261	**0.003052739**	**0.04742648**	1.005837	0.9527663	0.9691037
Complement C1r subcomponent	-1.606808	0.1558698	0.3967481	-2.636934	**0.01465771**	**0.04654091**
Cathepsin G	-1.609794	**0.01190246**	0.1029252	-1.286491	0.1318517	0.2307908
C-X-C motif chemokine 13	-1.685793	**0.0397383**	0.1995106	-1.340313	**0.04777114**	0.111523
Stanniocalcin-1	-1.717797	**9.52099E-05**	**0.006212448**	1.055758	0.4692365	0.5843069
Interleukin-1 receptor antagonist protein	-1.755324	**0.01023405**	0.09608225	-1.219914	0.2026755	0.3165606
Endothelial cell-specific molecule 1	-1.794750	**4.56486E-05**	**0.003309524**	-1.153998	0.06146679	0.1354969
Macrophage colony-stimulating factor 1	-1.834585	**0.005990576**	0.07138826	-1.201311	0.07157245	0.1516267
Neutrophil collagenase	-1.878701	**0.00176211**	**0.03537775**	-1.822236	**0.00247993**	**0.01170911**
C-C motif chemokine 22	-1.952447	**0.001210459**	**0.02771313**	-1.201742	0.2136068	0.326356
C3a anaphylatoxin	-2.003374	**0.007488924**	0.08144205	-1.586736	0.06589259	0.1428403
Tumor necrosis factor-inducible gene 6 protein	-2.023975	**0.000901867**	**0.02318205**	-1.125985	0.2490737	0.3611569
Hepcidin	-2.064608	**1.57337E-07**	**0.000102662**	-1.000161	0.9990991	0.9999265
Activated Protein C	-2.415783	**0.000655014**	**0.01987892**	-3.186877	**9.16668E-05**	**0.00093457**
Protein S100-A12	-2.503176	**3.95704E-05**	**0.003037613**	-2.264325	**0.000390664**	**0.00283231**
C-C motif chemokine 4-like	-2.788545	**0.000113743**	**0.00706831**	-1.470847	0.08135641	0.1677253
Plasminogen activator inhibitor 1	-2.803560	**5.64918E-05**	**0.003880093**	-1.370251	**0.04369913**	0.103875
C-C motif chemokine 2	-3.041445	**0.000318576**	**0.01299193**	-1.023771	0.8802179	0.9186173
Matrix metalloproteinase-9	-3.417954	**2.78776E-06**	**0.000454754**	-2.707313	**0.000132425**	**0.00126842**
Bactericidal permeability-increasing protein	-3.777211	**0.000273718**	**0.01190674**	-2.579768	**0.003946388**	**0.01661302**
Myeloblastin	-3.786105	**1.20749E-05**	**0.001125549**	-2.567718	**0.004340137**	**0.01803783**
C-C motif chemokine 7	-4.089323	**0.004499379**	0.05779224	-1.352108	0.05275771	0.1207874
C-X-C motif chemokine 6	-4.283902	**0.000184498**	**0.008964528**	-1.454786	0.1112449	0.2080563
C-X-C motif chemokine 10	-4.325095	**0.01589382**	0.1205897	1.325830	0.07129948	0.151294
Interleukin-1 receptor-like 1	-5.053614	**4.78343E-07**	**0.000190103**	-2.308034	**0.000207118**	**0.00177738**
Neutrophil elastase	-5.552423	**0.000195828**	**0.008964528**	-2.877038	**0.01145663**	**0.03833566**
C-X-C motif chemokine 11	-6.139843	**0.01263921**	0.1085142	-1.050483	0.8049845	0.8589572
Growth-regulated alpha protein	-6.845998	**4.26718E-06**	**0.000556867**	-2.845586	**0.000147502**	**0.00139485**
Myeloperoxidase	-6.862904	**8.94952E-06**	**0.000898394**	-4.320834	**0.000795717**	**0.00472005**
Interleukin-6	-9.046682	**6.38441E-08**	**8.33165E-05**	-7.991235	**6.14731E-06**	**0.00012734**
Interleukin-8	-11.379570	**1.35757E-06**	**0.000354326**	-2.757641	**5.17091E-05**	**0.00058836**

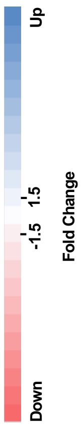

Supplementary Figure 17. CSF proteins with fold change >1.5 with significant p-value and adjusted p-value following control night and acute sleep deprivation, side by side comparison. Proteins are organized by descending value of fold change in control samples. Tables are a continuation from left to right. Protein fold change is colored by directionality of 1.5 fold change as follows: blue=upregulated, red=downregulated, and white= less than 1.5 fold change on either direction or no significant change. The color gradient represents magnitude of fold change. Bolded in red are the significant values p<0.05. Of note some of the proteins included have only a significant p-value. SlpD=sleep deprived. T24=Hour 24, T0= Hour 0.
